# A Fluctuation Equation of State for Prediction of High-Pressure Densities of Ionic Liquids

**DOI:** 10.1038/s41598-017-06225-9

**Published:** 2017-07-17

**Authors:** Mirosław Chorążewski, Eugene B. Postnikov, Bernadeta Jasiok, Yuriy V. Nedyalkov, Johan Jacquemin

**Affiliations:** 1 Institute of Chemistry, Department of Physical Chemistry, University of Silesia in Katowice, Szkolna 9, 40-006 Katowice, Poland; 2 Department of Theoretical Physics, Kursk State University, Radishcheva st., 33, 305000 Kursk, Russia; 3 School of Chemistry and Chemical Engineering, Queen’s University Belfast, Belfast, BT9 5AG UK; 4 Université François Rabelais, Laboratoire PCM2E, Parc de Grandmont, 37200 Tours, France

## Abstract

During this work, we demonstrate, for the first time, that the volumetric properties of pure ionic liquids could be truly predicted as a function of temperature from 219 K to 473 K and pressure up to 300 MPa. This has been achieved by using only density and isothermal compressibility data at atmospheric pressure through the Fluctuation Theory-based Tait-like Equation of State (FT-EoS). The experimental density data of 80 different ionic liquids, described in the literature by several research groups as a function of temperature and pressure, was then used to provide comparisons. Excellent predictive capability of FT-EoS was observed with an overall relative absolute average deviation close to 0.14% for the 15,298 data points examined during this work.

## Introduction

Ionic liquids (ILs) correspond to a large class of compounds with specific properties, such as high ionic conductivity, polarity, thermal and chemical stability, non-flammability and non-volatility^1^. Such unique profiles allow ILs to be good replacements for traditional organic solvents for various applications within the fields of catalysis^2,3^ and energy storage^4^, for example. A large number of research groups have then proved that applications could be then moved from laboratory scale to the industry^5^. To develop novel applications, it is vital to have access to their thermodynamic properties, such as the density, over a large range of temperature and pressure. Even if several groups have reported *p*-*ρ*-*T* data for several ILs^6–82^, these data are still lacking within respect to the possible number of IL combinations^5^. One solution to this problem is to develop novel models reliant on very few experimental data, based on a limited number of adjustable parameters and able to predict accurately ILs properties for a wide range of structures as a function of temperature and pressure. To date, different methods have been reported into the literature to correlate, evaluate and/or predict the volumetric properties of ILs^29,53,66,70,83–103^. These methods are mainly based on (*i*) the group contribution model (GCM)^53,83–91^, (*ii*) the equation of state (EoS)^29,66,70,92–96^, (*iii*) the quantitative structure-property relationship (QSPR)^97–100^, (*iv*) the artificial neural network (ANN)^101^, and (*v*) simple cross correlations between density and other physical properties^102,103^. Even if all proposed methods showed good predictive capability they are, in general, developed within the prior knowledge of density data over a wide range of temperature and pressure. As high-pressure density data are still lacking and less accessible than equivalent data at atmospheric pressure, the development of a novel and simple model requesting only the temperature dependence on volumetric properties of ILs at atmospheric pressure is vital. Recently, such an approach, so called the Fluctuation Theory-based Tait-like Equation of State (FT-EoS), has been proposed to predict density data over a wide range of temperature and pressure using solely volumetric properties at atmospheric pressure^104^. Its high accuracy was already assessed to a wide range of substances including halogenated and polar liquids^105–107^. During this work, we decided to further assess its predictive capability for the high-pressure density of 80 different ILs using experimental data available into the literature as the function of temperature and pressure (see Supplementary Table S1)^6–82^.

## Results

Herein, the density data of 80 different ionic liquids (ILs) were predicted and assessed using 15,298 data points from the literature (see Supplementary Table S1)^6–82^ using the Fluctuation Theory-based Tait-like Equation of State, which can be written along isotherms *T* = const as:

1 $$\rho ={\rho }_{0}+\frac{1}{k}\,\mathrm{log}[\frac{Mk}{\nu RT}(P-{P}_{0})+1].$$

The reference pressure *P*_0_ is assumed to be equal to atmospheric pressure; *M* and *R* are the molar mass and the gas constant, respectively. Two main control parameters in Eq. (1), *v* and *k*, are the functions of the density, *ρ*_0_ and the isothermal compressibility ${\kappa }_{T}^{0}$ defined for each temperature as

2 $$\nu =\frac{M}{RT}\frac{1}{{\rho }_{0}{\kappa }_{T}^{0}},k=-\frac{1}{{\rho }_{0}}-{(\frac{d{\rho }_{0}}{dT})}^{-1}[\frac{1}{T}+\frac{d\,\mathrm{log}\,{\kappa }_{T}^{0}}{dT}]$$

at this pressure below the boiling temperature *T*_*b*_. They can be easily determined from experimental data as a reference state: the direct measurements of density, which give also the isobaric expansion coefficient ${\alpha }_{P}=-{\rho }_{0}^{-1}{(\partial {\rho }_{0}/\partial T)}_{P={P}_{0}}$ for *T* < *T*_*b*_. The last one combined with the measured speed of sound *c*_0_ and the isobaric heat capacity ${c}_{P}^{0}$ provides the values of the isothermal compressibility

3 $${\kappa }_{T}^{0}=\frac{1}{{\rho }_{0}{c}_{0}^{2}}+\frac{T{\alpha }_{P}^{0}}{{\rho }_{0}{c}_{P}^{0}}.$$

Thus, an application of Eq. (1) does not require, in principle, any high-pressure measurement or empiric correlations to determine these parameters, such unique behavior is, in fact, the main advantage of the proposed method in comparison with those already available in the literature^29,53,66,70,83–103^.

**Figure 1:**
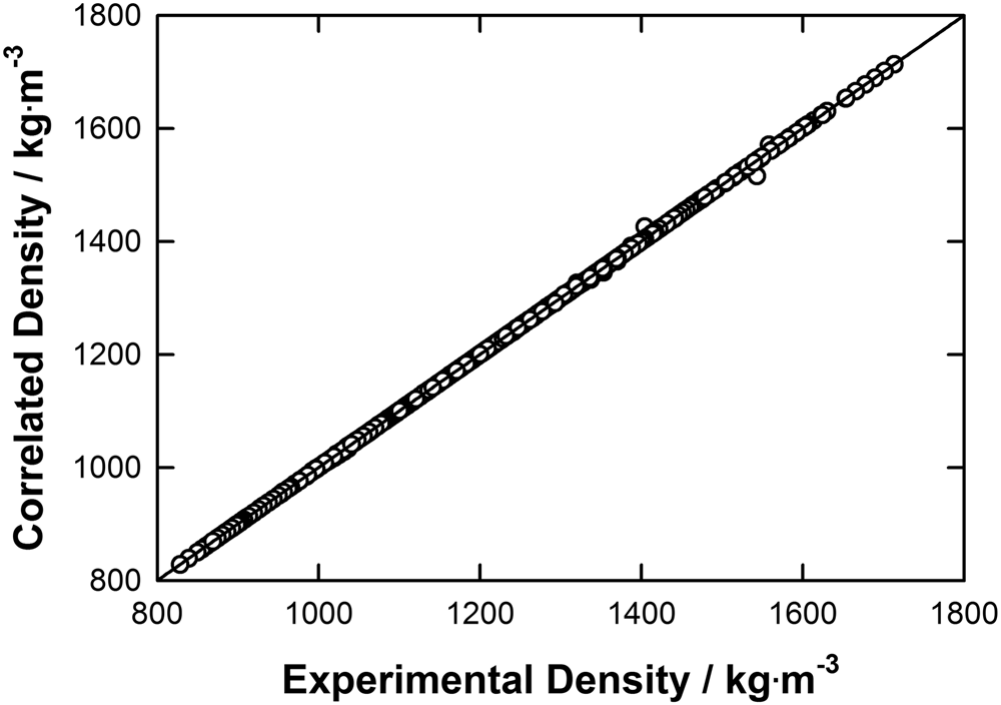
Comparison of experimental and correlated density data using Eq. (13) at atmospheric pressure for selected ionic liquids.

The practical application of the procedure described for an arbitrary *T* < *T*_*b*_, certainly requires introduction of some continual function fitting the discrete set of experimental data. During this work, various density and isothermal compressibility datasets at atmospheric pressure were used then to truly predicted the volumetric properties of 80 selected ILs as a function of temperature from 219 K to 473 K and pressure up to 300 MPa. As reported in the Supplementary Table S2, these atmospheric pressure input data, especially for the density, are mainly coming directly from the reported literature values. However, in some cases, no isothermal compressibility value was originally reported for a given IL structure studied solely by one research group, for example. In such a situation, this missing input has been determined herein at atmospheric pressure following this order of preference (*i*) by applying the Tait equation for reported density values; (*ii*) by using Eq. (3) if the speed of sound and the isobaric heat capacity were also reported in the reference paper; or alternatively, when (*i*) and (*ii*) were not applicable, by applying the GCM proposed by Jacquemin *et al*.^53,83^. As shown in Fig. 1 and depicted in the ESI for each investigated IL, by applying this data collection methodology, 1,377 data points were used to correlate the temperature dependence on the density at atmospheric pressure by cubic polynomials within an excellent accuracy close to 0.02%. However, as no density value was reported at atmospheric pressure for the ILs [C_6_mim][OTf]^14^, [C_4_mim][(C_2_F_5_)_3_PF_3_]^68^, [C_6_mim][(C_2_F_5_)_3_PF_3_]^68^, and [P_66614_][OAc]^65^; in these cases, requested data were evaluated by using the GCM developed by Jacquemin *et al*.^53,83^.

**Figure 2:**
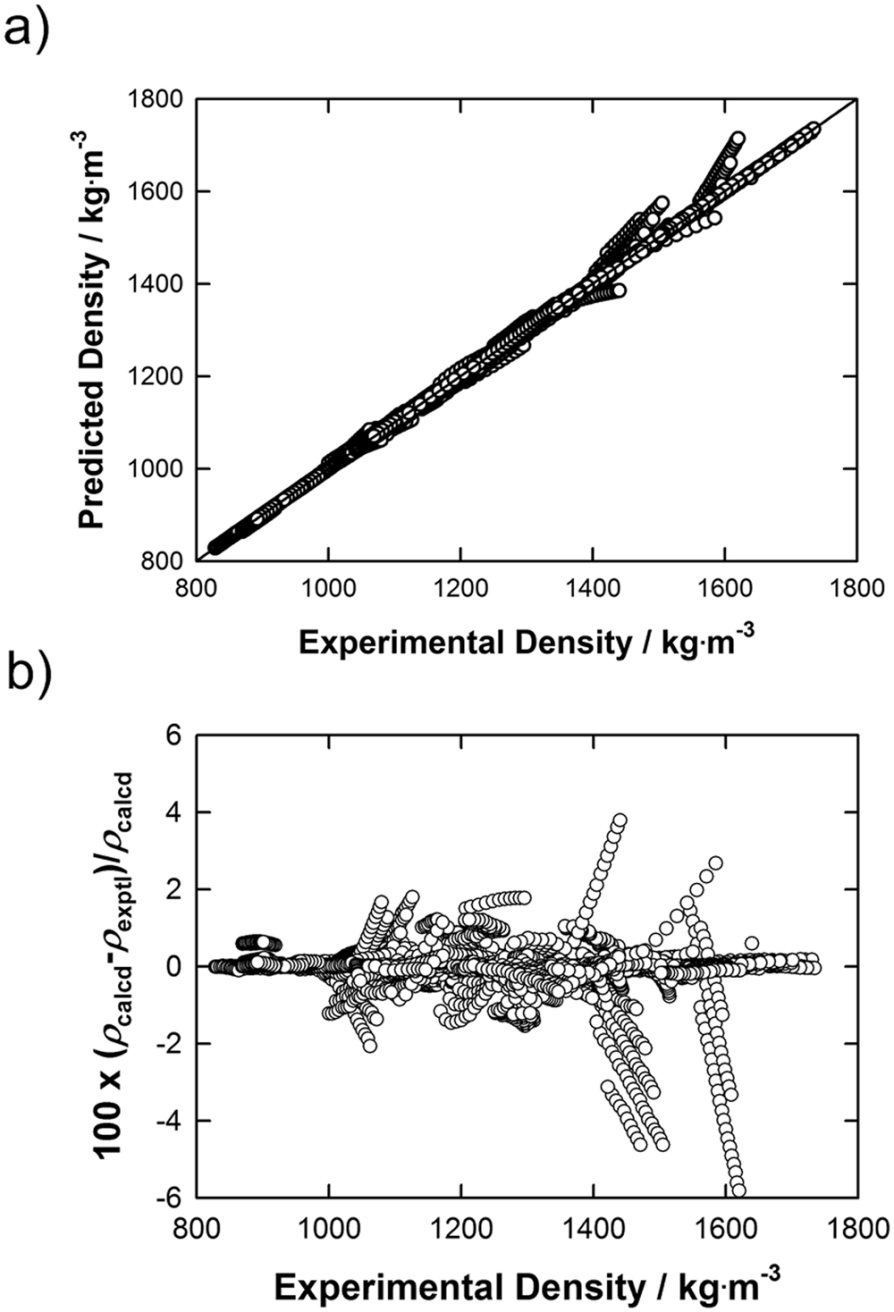
Plots of experimental versus: (**a**) predicted high-pressure density data for ILs; and (**b**) relative deviations between experimental (*ρ*_exptl_) and predicted (*ρ*_calcd_) data obtained by using the proposed FT-EoS.

Similarly, the temperature dependence on the ILs isothermal compressibility was correlated using data directly reported in the literature, or, alternatively, calculated using the approach stated above (*i*.*e*. using the Tait equation, or using thermodynamics formalism or using the GCM developed by Jacquemin *et al*.^53,83^) as reported in the Supplementary Table S2. By applying this FT-EoS approach for selected ILs, as shown in Fig. 2 and exemplified in the ESI for each IL, excellent agreement is observed between experimental^6–82^, and predicted high-pressure density data for all ILs investigated during this work.

This excellent predictive capability was attested by comparing 13,921 high-pressure density data points from the literature for the 80 ILs with those predicted by this method within an overall relative average absolute deviation (RAAD) close to 0.14%. Furthermore, this overall RAAD value also attests the higher ability of this FT-EoS to truly predict the volumetric properties of ILs over a wide range of temperature and pressure in comparison with other models reported in the literature^29,53,66,70,83–103^, to date. For example, in the case of the [C_4_mim][NTf_2_], *e*.*g*. one of the most studied IL, an overall RAAD close to 0.06% is observed between data reported in the literature^15,26,50,55,56,81,82^, with those determined herein using the FT-EoS equation, while RAADs close to 0.48%, 0.29%, 0.20%, 0,47%, 0.33% are observed using the methods reported by Jacquemin *et al*.^53^, Paduszyński *et al*.^90^, Gardas *et al*.^86^, Lazzuz^87^, Qiao *et al*.^88^, respectively. This is further attested by comparing also the number of adjustable parameters associated to these reported models, while only two input pairs of parameters (*e*.*g*. temperature dependences on the density and on the isothermal compressibility at atmospheric pressure) are requested for the proposed FT-EoS.

## Discussion

By depicting comparisons made within the literature, as shown in Supplementary Tables S1 and S2, it appears that larger errors are observed for: (*i*) ILs density data described by more than one research group; (*ii*) hydrophilic and/or water sensitive ILs and (*iii*) datasets predicted using GCM density data at atmospheric pressure. For example, the largest RAAD, which is close to 1.8%, was observed for the [C_2_mim][PF_6_] using extrapolated density data at atmospheric pressure reported by Taguchi *et al*.^12^. Similarly, using the high-pressure data reported by Klomfar *et al*.^14^ for the [C_2_mim][BF_4_] along with atmospheric input data reported by Gardas *et al*.^11^ a RAAD close to 1.3% was achieved, while excellent agreements were observed with other datasets published in the literature for this IL (0.02%^11^,0.03%^13^,and 0.13%^12^). In the case of the [C_4_mim][BF_4_], larger deviations were observed between predicted density values with datasets reported by Tekin *et al*.^25,28^. (RAAD = 0.99% for the two identical datasets published by this group) and by Harris *et al*.^27^. (RAAD = 0.88%) while RAAD better than 0.3% were observed for the other datasets^13,21–24,26,29–34^ available in the literature showing a clear discrepancy between the experimental data of this water sensitive IL^39^. Interestingly, it appears that the missing input data at atmospheric pressure could, *a priori*, be evaluated by the GCM developed by Jacquemin *et al*.^53,83^ and then used in the FT-EoS to predict accurately high-pressure density data as exemplified for the [C_6_mim][OTf] (RAAD = 1.04%^14^), [C_4_mim][(C_2_F_5_)_3_PF_3_] (RAAD = 0.02%^68^), [C_6_mim][(C_2_F_5_)_3_PF_3_] (RAAD = 0.16%^68^), and [P_66614_][OAc] (RAAD = 0.61%^65^), for example. Similarly, as demonstrated herein in the case of the [C_4_mim][PF_6_], high-pressure speeds of sound could be used to determine the ILs isothermal compressibility data at atmospheric pressure, which could be then used as the input data for the FT-EoS. For example, by using the calculated isothermal compressibility reported by Gomes de Azevedo *et al*.^22^, excellent agreement is observed between predicted and experimental [C_4_mim][PF_6_] high-pressure density data published by Jacquemin *et al*.^26^ (RAAD = 0.14%), Machida *et al*.^29^ (RAD = 0.19%) and Tomida *et al*.^79^ (RAAD = 0.06%). Interestingly, by comparing input data determined thanks to the thermodynamics formalism (Eq. (3))^22^ and those from the GCM developed by Jacquemin *et al*.^53,83^ RAAD close to 1.5% on their calculated isothermal compressibility data for the [C_4_mim][PF_6_] is observed (see Supplementary Table S3). These two approaches lead then to a RAAD between the high-pressure density data reported by Azevedo *et al*.^22^ and those calculated herein using the FT-EOS close to 0.02% or 0.01% using isothermal compressibility data from the Eq. (3) or the Jacquemin *et al*. GCM^53,83^, respectively (see Supplementary Figure S1). This furthermore highlights the possibility to determine missing isothermal compressibility input by following one of these two approaches with a great accuracy.

Finally, by comparing the performance of the proposed FT-EoS approach with other methods described in the literature, it appears that a better prediction of high-pressure density data is achieved by using the FT-EoS presented herein, compared to those reached using GCMs available in the literature as an overall RAAD close to 0.36%, 0.45% or better than 1.45% was reported by Jacquemin *et al*.^53^, Paduszyński *et al*.^90^ or Gardas *et al*.^86^, for example. Nevertheless, the main advantage of these GCMs is to be able to estimate the density of unknown ILs, which is not the case of the FT-EoS. However, as exemplified herein, it is possible to combine these two approaches by evaluating the requested input data at atmospheric pressure using a GCM to feed the FT-EoS for unknown IL structures demonstrating the great potential of the FT-EoS approach described in this work.

## Method

Briefly, the FT-EOS acts in the context of thermodynamic approaches to the derivation of an equation of state based on the consideration of the reduced elastic bulk modulus $K={\kappa }_{T}^{-1}$, where the isothermal compressibility is defined as:

4 $${\kappa }_{T}=-\frac{1}{V}{(\frac{\partial V}{\partial P})}_{T}=\frac{1}{\rho }{(\frac{\partial \rho }{\partial P})}_{T},$$

expressing the changes of specific volume *V* = *ρ* ^−1^ or density caused by the change of the external pressure applied to a fluid.

This approach generalizes the stress-strain relationship from the classic theory of elasticity^108^, where the small homogeneous compression gives in the linear approximation: *u*_*ii*_ = (3*K*_0_)^−1^ *σ*_*ii*_. Here, the sum of diagonal elements of the strain tensor *u*_*ii*_ describes the relative change in the volume while the stress tensor is equal to *σ*_*ik*_ = −*pδ*_*ik*_ (where *δ*_*ik*_ is the Kronecker symbol, and *p* = *dP* is the excess applied pressure). As a result, this elastic bulk modulus satisfies Hooke’s law:

5 $$-\frac{1}{{V}_{0}}{(\frac{\partial V}{\partial P})}_{T}=\frac{1}{{K}_{0}},$$

where *V*_0_ denotes the strainless volume, and *K*_0_ = const independent on the pressure for infinitesimal deformations.

By considering finite deformations of liquids, Tait^109^ proposed the following linear correction, which could be applied with the respect to the pressure change:

6 $$K(p)=-{V}_{0}{(\frac{{\rm{\Delta }}P}{{\rm{\Delta }}V})}_{T}={K}_{0}+K^{\prime} (P-{P}_{0}),$$

where ${P}_{0}$ is the reference ambient pressure at the uncompressed state.

The differential replacement of finite differences proposed by Tammann^110^

7 $$K(p)=-{V}_{0}{(\frac{\partial P}{\partial V})}_{T}={K}_{0}+K^{\prime} (P-{P}_{0}),$$

gives after integration the classic Tait equation, which can be written in terms of density *ρ* = *V* ^−1^ as follows:

8 $$\rho =\frac{{\rho }_{0}}{1-K{^{\prime} }^{-1}\,\mathrm{log}[{\kappa }_{T}^{0}K^{\prime} (P-{P}_{0})+1]}.$$

The Tait equation Eq. (8) relates to the isothermal compression to the initial reference state, where the parameter ${K}_{0}^{-1}={\kappa }_{T}^{0}$ is the isothermal compressibility under the reference pressure *P*_0_. One can highlight, however, that such an assumption results in the non-physical existence of the maximal pressure *P*_max_, which corresponds to *ρ* = ∞ (*V* = 0) and negative values of the density/volume for larger pressures, although these parameters are extremely high for reasonable practical applications. In addition, *K*′ remains, in Eq. (8), a purely empirical parameter^111^.

Note that there is an alternative to the choice of the reference volume, which allows for avoiding the mentioned negative densities, when the specific volume of the compressed medium is chosen instead of the initial one, as proposed by Murnaghan^112^ in the context of the compressibility of elastic solids under extremely high pressures, that also can been applied to liquids^111^. But such an approach keeps at last one purely empirical constant too and has a similar range of accuracy as the Tait equation for low elevated pressures.

Thus, Eq. (9), given below, could be then obtained by replacing the fraction of the original Tait equation (Eq. (8)) by the first terms of its Taylor’s expansion taking into account the fact that the subtrahend in the denominator is a small quantity for realistic *PρT* conditions of liquids.

9 $$\rho ={\rho }_{0}+{\rho }_{0}K{^{\prime} }^{-1}\,\mathrm{log}[{\kappa }_{T}^{0}K^{\prime} (P-{P}_{0})+1].$$

This form is free from non-physical negative density values and implies the exponential functional dependence along isotherms (∂*ρ*/∂*P*)_*T*_ ~exp(−*kρ*) with $k=K^{\prime} {\rho }_{0}^{-1}$.

Quantitatively, there is an experimental evidence for a large variety of classes of liquids^105,107,113,114^ that the accurate exponential dependence for different temperatures fulfils for the dimensionless complex:

10 $$\nu =\frac{M}{RT}\frac{1}{\rho {\kappa }_{T}},$$

where *T*, *M* and *R* are the temperature, the molar mass and the gas constant.

The original Eq. (1), used therein to predict the volumetric properties of ILs as a function of temperature and pressure, is obtained by expressing *K*′ and ${\kappa }_{T}^{0}$ through *k* and *v*.

Note also that Eq. (10) has a statistical meaning as an inverse ratio of the relative volume fluctuation to its value in the hypothetical case where the liquid acts as an ideal gas for the same temperature-volume parameters ${\nu }^{-1}=\langle {\rm{\Delta }}{V}^{2}\rangle /\langle {\rm{\Delta }}{V}_{ig}^{2}\rangle$ (see refs^104,113^ for details), or as a ratio of the corresponding elastic isothermal bulk moduli *v* = *K*/*K*_*ig*_, since *K*_*ig*_ = *M* ^−1^ *ρRT*.

Therefore, from the point of view of the linear expansion of the elastic bulk modulus with respect to the pressure, Eq. (9) leads to the following relation:

11 $$K(p)=-V{(\frac{\partial P}{\partial V})}_{T}=\frac{{V}_{0}}{V}[{K}_{0}+K^{\prime} (P-{P}_{0})].$$

The right-hand side of Eq. (11) can be considered as a weighted average of the solid state (Hooke’s equation) and an ideal gas moduli (*K*_*ig*_ = *M* ^−1^ *ρRT* = *P*, from the Clapeyron’s EoS) multiplied by the ratio of volumes, which provides a more accurate representation of the stress-strain conditions since both coefficients *K* and *K*′ are defined for uncompressed state, and *K*(*p*) for a compressed state. In fact, this ratio compensates for the difference between the force densities acting within these two states. This allows for interpreting of the origin of the pressure-dependent bulk modulus Eq. (11). As a bulk modulus of composite medium is formed by elastic dense molecular complexes separated by holes, *i*.*e*. an ideal gas of compressible clusters (see ref.^113^ for simulations of lattice liquid system in comparison with saturated liquefied noble gases behavior, and^115^ for some more abstract theory of similar systems. This conclusion is also supported by the experimental fact of violation of such exponential dependence at high pressures corresponding to the conditions of contact percolation transition^116^.

Since the parameter *k* can be determined as a local (pointwise) slope of the tangent to the function log*v* = *kρ*_0_ + *b*, which can be calculated from experimental data measured at atmospheric pressure for those temperatures, this approach requires some special improvements in the procedure of parameter determination for the FT-EoS Eq. (1).

The desired derivative was calculated using the following parametric differentiation:

12 $$k=\frac{d\,\mathrm{log}\,\nu }{d\rho }\equiv \frac{\frac{d\,\mathrm{log}\,\nu }{dT}}{\frac{d\rho }{dT}}=-\frac{1}{\rho }-{(\frac{d\rho }{dT})}^{-1}[\frac{1}{T}+\frac{d\,\mathrm{log}\,{\kappa }_{T}}{dT}].$$

One can see that Eq. (12) formally contains the derivative of dimensional isothermal compressibility, however, the selected *κ*_T_ unit does not influence completely the solution of Eq. (12) due to the presence of the logarithm function. To exemplify this point let us represent ${\kappa }_{T}={\tilde{\kappa }}_{T}(T)[{\kappa }_{T}]$, where ${\tilde{\kappa }}_{T}(T)$ and [*κ*_*T*_] denote the functional temperature dependence and the selected unit (arbitrary selected but the same unit must be used for all investigated temperature points) of *κ*_T_ data, respectively. Whence, $\frac{d\,\mathrm{log}\,{\kappa }_{T}}{dT}=\frac{d\,\mathrm{log}\,{\tilde{\kappa }}_{T}}{dT}+\frac{d\,\mathrm{log}\,[{\kappa }_{T}]}{dT}=\frac{d\,\mathrm{log}\,{\tilde{\kappa }}_{T}}{dT}$ since $\mathrm{log}({\tilde{\kappa }}_{T}(T)[{\kappa }_{T}])=$ $\mathrm{log}\,{\tilde{\kappa }}_{T}(T)+\,\mathrm{log}\,[{\kappa }_{T}]\,{\rm{and}}\,\mathrm{log}\,[{\kappa }_{T}]={\rm{const}}$. For the same reason, Eq. (12) does not contain the combination *M*/*R*, as expected by combining Eq. (10) into Eq. (12).

In practice, the density and the logarithm of the isothermal compressibility can be fitted with a high accuracy within appropriate temperature intervals by quadratic polynomials of the temperature:

13 $$\begin{array}{rcl}{\rho }_{{\rm{0}}} & = & {a}_{2}{T}^{2}+{a}_{1}T+{a}_{0},\\ \mathrm{log}\,{\kappa }_{{\rm{T}}} & = & a{^{\prime} }_{2}{T}^{2}+a{^{\prime} }_{1}T+a{^{\prime} }_{0}.\end{array}$$

Therefore, Eqs (10) and (12) take the forms, respectively

14 $$\nu =\frac{M}{RT}\frac{\exp [-(a{^{\prime} }_{2}{T}^{2}+a{^{\prime} }_{1}T+a{^{\prime} }_{0})]}{{a}_{2}{T}^{2}+{a}_{1}T+{a}_{0}},$$

and

15 $$k=-\frac{1}{{a}_{2}{T}^{2}+{a}_{1}T+{a}_{0}}-\frac{1+T(2a{^{\prime} }_{2}T+a{^{\prime} }_{1})}{T(2{a}_{2}T+{a}_{1})}.$$

Finally, the values given by Eqs (14) and (15) for each temperature should be substituted into Eq. (1), as well as, the density at atmospheric pressure to determine explicitly the density at a given temperature and pressure.

During this work, practical calculations were performed with a user-defined function written in VBA language for MS Excel (see Supplementary spreadsheet FT-EoS_calc_template.xlsm). The input variables are the pairs “temperature-density” and “temperature-isothermal compressibility” filled by experimental data measured at the normal atmospheric pressure (at least 4 data points are requested) processed to obtain the coefficients in Eq. (13) *via* the least-square polynomial fit using the build-in MS Excel procedure (Application.LinEst). They are used subsequently in Eqs (14) and (15), which are substituted into the final expression (Eq. (1)) applied for the predictive calculation of the density.

### Data availability

A summary of all the experimental data collected from the literature, data input used, methodology applied and obtained prediction results are presented in Supplementary Tables S1–S3 and illustrated in Figure S1 in the case of the [C_4_mim][PF_6_]. The supplementary MS Excel spreadsheet FT-EoS_calc_template.xlsm contains the build-in VB procedure (FTEOS) allows calculation of high-pressure density of liquids using fluctuation equation of state.

## Conclusion

In the light of this work, one can conclude that the proposed FT-EoS approach can be used to truly predict with a high accuracy the density of liquids under elevated pressures. This approach requests, solely, the prior knowledge of the temperature dependences on physical data (such as the speed of sound, the density, and the isobaric heat capacity) at normal ambient atmospheric pressure, which can be easily obtained experimentally. One can further note that this approach is not based on a pure correlation but on the general elastic properties of isothermal compression of elastic media. Finally, the validity of this method has been checked herein through the comparison between predicted and experimental high-pressure density data for an extensive set of data covering a large range of temperatures and pressures for 80 different ionic liquids within an accuracy close to 0.14%.

## Electronic supplementary material

Supporting Information

Supporting Information
