# Author Correction: A Fluctuation Equation of State for Prediction of High-Pressure Densities of Ionic Liquids

**DOI:** 10.1038/s41598-017-14855-2

**Published:** 2017-11-22

**Authors:** Mirosław Chorążewski, Eugene B. Postnikov, Bernadeta Jasiok, Yuriy V. Nedyalkov, Johan Jacquemin

**Affiliations:** 1 Institute of Chemistry, Department of Physical Chemistry, University of Silesia in Katowice, Szkolna 9, 40-006 Katowice, Poland; 2 Department of Theoretical Physics, Kursk State University, Radishcheva st., 33, 305000 Kursk, Russia; 3 School of Chemistry and Chemical Engineering, Queen’s University Belfast, Belfast, BT9 5AG UK; 4 Université François Rabelais, Laboratoire PCM2E, Parc de Grandmont, 37200 Tours, France

*Scientific Reports* **7**:5563; doi:10.1038/s41598-017-06225-9; Article published online 17 July 2017

One of the Supplementary Information files that accompany this study was inadvertently omitted in the original version of this Article. The link to this Supplementary Information file has now been added to the HTML version of the Article.

