# Supplementary material for: A Fluctuation Equation of State for Prediction of High-Pressure Densities of Ionic Liquids
Source: Sci Rep. 2017 Jul 17;7:5563. doi: 10.1038/s41598-017-06225-9 (PMC5514048; doi:10.1038/s41598-017-06225-9)
Supplement: Supplementary file 1 — Supporting Information [file 41598_2017_6225_MOESM1_ESM.doc]

A Fluctuation Equation of State for Prediction of High-Pressure Densities of Ionic Liquids

Mirosław Chorążewski^1*^, Eugene B. Postnikov^2*^,

Bernadeta Jasiok^1^, Yuriy V. Nedyalkov^2^, Johan Jacquemin^3,4^

^1^Institute of Chemistry, Department of Physical Chemistry, University of Silesia in Katowice, Szkolna 9, 40-006, Katowice, Poland.

^2^Department of Theoretical Physics, Kursk State University, Radishcheva st., 33, 305000, Kursk, Russia.

^3^School of Chemistry and Chemical Engineering, Queen's University Belfast, Belfast, BT9 5AG, U.K.

^4^Université François Rabelais, Laboratoire PCM2E, Parc de Grandmont 37200 Tours, France

* Corresponding author’s e-mail: [*miroslaw.chorazewski@us.edu.pl*](mailto:miroslaw.chorazewski@us.edu.pl), [*postnicov@gmail.com*](mailto:postnicov@gmail.com)

Supplementary Information

ABBREVIATIONS

| **Cations:** | | **Anions:** | |
| --- | --- | --- | --- |
| [C_1_mim]^+^ | 1,3-dimethylimidazolium | [B(CN)_4_]^-^ | tetracyanoborate |
| [C_2_mim]^+^ | 1-ethyl-3-methylimidazolium | [BF_4_]^-^ | tetrafluoroborate |
| [C_2_eim]^+^ | 1,3-diethylimidazolium | [C(CN)_3_]^-^ | tricyanomethanide |
| [C_3_mim]^+^ | 1-propyl-3-methylimidazolium | [NTf_2_]^-^ | bis(trifluoromethylsulfonyl)imide |
| [C_4_mim]^+^ | 1-butyl-3-methylimidazolium | [C_1_SO_4_]^-^ | methylsulfate |
| [C_5_mim]^+^ | 1-pentyl-3-methylimidazolium | [C_2_SO_4_]^-^ | ethylsulfate |
| [C_6_mim]^+^ | 1-hexyl-3-methylimidazolium | [C_6_SO_4_]^-^ | hexylsulfate |
| [C_7_mim]^+^ | 1-heptyl-3-methylimidazolium | [C_8_SO_4_]^-^ | octylsulfate |
| [C_8_mim]^+^ | 1-octyl-3-methylimidazolium | [C_1_SO_3_]^-^ | methanesulfonate |
| [C_10_mim]^+^ | 1-decyl-3-methylimidazolium | [PF_6_]^-^ | hexafluorophosphate |
| [C_4_C_1_mim]^+^ | 1-butyl-2,3-dimethylimidazolium | [DCA]^-^ | dicyanamide |
| [amim]^+^ | 1-allyl-3-methylimidazolium | [MP]^-^ | methylphosphate |
| [C_3_mpip]^+^ | 1-propyl-1-methylpiperidinium | [DMP]^-^ | dimethylphosphate |
| [C_3_py]^+^ | 1-propylpyridinium | [DEP]^-^ | diethylphosphate |
| [C_4_py]^+^ | 1-butylpyridinium | [Lactate]^-^ | L-lactate |
| [C_6_py]^+^ | 1-hexylpyridinium | [SCN]^-^ | thiocyanate |
| [C_8_py]^+^ | 1-octylpyridinium | [OAc]^-^ | acetate |
| [p3mpy]^+^ | 1-propyl-3-methylpyridinium | [C_2_COO]^-^ | propionate |
| [b2mpy]^+^ | 1-butyl-2-methylpyridinium | [C_3_COO]^-^ | butyrate |
| [b3mpy]^+^ | 1-butyl-3-methylpyridinium | [C_4_COO]^-^ | pentanoate |
| [b4mpy]^+^ | 1-butyl-4-methylpyridinium | [OTf]^-^ | triflate |
| [o3mpy]^+^ | 1-octyl-3-methylpyridinium | [(C_2_F_5_)_3_PF_3_]^-^ | tris(pentafluoroethyl)trifluorophosphate |
| [C_1_OC_2_mpyrro]^+^ | 1-(2-methoxyethyl)-1-methyl-pyrrolidinium | [Tos]^-^ | tosylate |
| [C_3_mpyrro]^+^ | 1-propyl-1-methylpyrrolidinium | Br^-^ | bromide |
| [C_4_mpyrro]^+^ | 1-butyl-1-methylpyrrolidinium | Cl^-^ | chloride |
| [N_1112OH_]^+^ | 2-hydroxyethyl-trimethylammonium |  |  |
| [N_1114_]^+^ | butyl-trimethyl-ammonium |  |  |
| [Aliquat]^+^ | methyl-trioctyl-ammonium |  |  |
| [C_2_OHC_1_NH_2_]^+^ | N-methyl-2-hydroxyethylammonium |  |  |
| [P_666 14_]^+^ | trihexyl(tetradecyl)phosphonium |  |  |

**Table S1. A summary of the literature data for ionic liquids used in the calculations.**

|  |  | **Atmospheric Pressure - Input Values** | | | **High-Pressure - Predicted Values** | | | |
| --- | --- | --- | --- | --- | --- | --- | --- | --- |
| **IL** | **Ref.** | **Numbers of Data** | **T_range_/ K** | **RAAD / %** | **Numbers of Data** | **T_range_ / K** | **P_range_/ MPa** | **RAAD / %** |
| [C_2_mim][SCN] | REF 6 | 5 | 298.15-338.15 | 0.0170 | 60 | 298.15-338.15 | 0.1-10 | 0.0177 |
| [C_2_mim][OAc] | REF 7 | 7 | 293.17-353.16 | 0.0083 | 56 | 293.14-353.2 | 0.1-25 | 0.1445 |
| [C_4_mim]Cl | REF 8 | 6 | 348.15-373.15 | 0.0023 | 120 | 352.1-452 | 0.1-200 | 0.3602 |
| [C_2_mim][DCA] | REF 10 | 18 | 278.15-363.15 | 0.0027 |  |  |  |  |
|  | REF 9 |  |  |  | 85 | 256.825-346.223 | 0.1-60.101 | 0.3216 |
| [N_1112OH_][Lactate] | REF 8 | 14 | 293.15-353.15 | 0.0031 | 100 | 312.4-392.4 | 0.1-200 | 0.0853 |
| [C_4_mim][SCN] | REF 6 | 5 | 298.15-338.15 | 0.0080 | 60 | 298.15-338.15 | 0.1-10 | 0.0058 |
| [C_2_mim][BF_4_] | REF 11 | 8 | 293.15-393.15 | 0.0085 | 88 | 293.15-393.15 | 0.1-30 | 0.0199 |
|  | REF 12 | 4 | 293.15-353.15 | 0.0016 | 180 | 312.2-472.4 | 0.1-200 | 0.1272 |
|  | REF 13 | 9 | 283.15-323.15 | 0.0010 | 108 | 283.15-323.15 | 0.1-60 | 0.0350 |
|  | REF 14 |  |  |  | 76 | 284.727-356.401 | 1.0554-61.191 | 1.2390 |
| [C_4_mim][OAc] | REF 7 | 7 | 298.12-353.06 | 0.0058 | 56 | 298.09-353.06 | 0.1-25 | 0.0185 |
|  | REF 82 | 9 | 293.15-373.15 | 0.0089 | 80 | 311.4-371.3 | 0.1-200 | 0.4301 |
| [C_2_mim][Lactate] | REF 8 | 13 | 293.15-353.15 | 0.0031 | 140 | 312.6-432.2 | 0.1-200 | 0.0610 |
| [C_4_mim][DCA] | REF 9 | 0 | 0 |  | 58 | 237.204-349.804 | 0.6766-60.158 | 0.1925 |
|  | REF 15 | 10 | 293.15-393.15 | 0.0026 | 160 | 293.15-393.15 | 0.1-60 | 0.0097 |
|  | REF 76 | 8 | 283.16-343.14 | 0.0029 | 96 | 283.13-393.17 | 0.1-99.916 | 0.0622 |
| [C_1_mim][C_1_SO_4_] | REF 16 | 4 | 318.15-333.15 | 0.0059 | 30 | 313.15-333.15 | 0.1-25 | 0.0566 |
|  | REF 17 | 12 | 318.15-428.15 | 0.0190 | 156 | 318.15-428.15 | 0.1-60 | 0.0756 |
| [b4mpy][SCN] | REF 6 | 5 | 298.15-338.15 | 0.0253 | 60 | 298.15-338.15 | 0.1-10 | 0.0242 |
| [C_2_mim][C_1_SO_4_] | REF 18 | 8 | 293.15-393.15 | 0.0224 | 72 | 293.15-393.15 | 0.1-35 | 0.2763 |
| [C_4_py][BF_4_] | REF 19 | 6 | 298.2-343.2 | 0.0096 | 12 | 298.2-323.2 | 0.1-204.18 | 0.1954 |
|  | REF 20 | 7 | 293.15-353.15 | 0.0072 | 28 | 293.15-353.15 | 0.1-20 | 0.0144 |
|  | REF 75 | 11 | 283.15-333.15 | 0.0046 | 143 | 283.15-333.15 | 0.1-65 | 0.0253 |
| [C_4_mim][BF_4_] | REF 13 | 9 | 283.15-323.15 | 0.0007 | 108 | 283.15-323.15 | 0.1-60 | 0.0583 |
|  | REF 21 | 11 | 278.15-333.15 | 0.0170 | 15 | 298.15-333.15 | 0.1-60 | 0.0818 |
|  | REF 22 | 5 | 298.34-332.73 | 0.0064 | 62 | 298.34-332.73 | 0.1-59.92 | 0.0581 |
|  | REF 23 | 4 | 293.15-353.15 | 0.0084 | 16 | 293.15-353.15 | 0.1-20 | 0.0545 |
|  | REF 24 | 11 | 293.15-393.15 | 0.0053 | 66 | 293.15-393.15 | 0.1-10 | 0.0207 |
|  | REF 25 / REF 28 |  |  |  | 45 | 298.15-398.15 | 0.21-39.85 | 0.9861 |
|  | REF 26 | 10 | 292.94-414.92 | 0.0189 | 36 | 293.49-414.93 | 0.1-40 | 0.0254 |
|  | REF 27 | 36 | 273.15-363.15 | 0.0093 | 40 | 283.15-348.15 | 0.1-300 | 0.8844 |
|  | REF 29 | 9 | 313.1-472.2 | 0.0287 | 180 | 313.1-472.2 | 0.1-200 | 0.2031 |
|  | REF 30 |  |  |  | 21 | 313.2-433.2 | 0.1-2 | 0.2957 |
|  | REF 31 | 3 | 298.15-323.15 | 0.0000 | 12 | 298.15-323.15 | 0.1-30 | 0.0924 |
|  | REF 32 | 5 | 283.15-333.15 | 0.0012 | 54 | 283.15-333.15 | 0.1-40 | 0.0795 |
|  | REF 33 |  |  |  | 61 | 239.754-354.156 | 0.1-60.815 | 0.0883 |
|  | REF 34 | 29 | 283.15-353.15 | 0.0091 | 232 | 283.15-353.15 | 0.1-35 | 0.0794 |
| [C_4_mim][C(CN)_3_] | REF 11 | 8 | 293.15-393.15 | 0.0179 | 88 | 293.15-393.15 | 0.1-30 | 0.0281 |
| [C_2_mim][C_2_SO_4_] | REF 15 | 12 | 293.15-433.15 | 0.0086 | 191 | 293.15-433.15 | 0.1-60 | 0.0089 |
|  | REF 18 | 8 | 293.15-393.15 | 0.0425 | 72 | 293.15-393.15 | 0.1-35 | 0.0847 |
|  | REF 34 | 29 | 283.15-353.15 | 0.0063 | 232 | 283.15-353.15 | 0.1-35 | 0.0156 |
|  | REF 35 | 7 | 283.15-333.15 | 0.0040 | 56 | 283.15-333.15 | 0.1-35 | 0.0120 |
|  | REF 36 | 13 | 283.15-343.15 | 0.0024 | 104 | 283.15-343.15 | 0.1-35 | 0.0147 |
|  | REF 37 | 14 | 278.15-398.15 | 0.0195 | 112 | 278.15-398.15 | 0.1-120 | 0.0493 |
|  | REF 40 | 7 | 278.15-398.15 | 0.0163 | 56 | 278.15-398.15 | 0.1-120 | 0.0460 |
| [b2mpy][BF_4_] | REF 41 | 9 | 293.15-333.15 | 0.0024 | 111 | 293.15-333.15 | 0.1-65 | 0.0168 |
| [b3mpy][BF_4_] | REF 41 | 11 | 283.15-333.15 | 0.0033 | 143 | 283.15-333.15 | 0.1-65 | 0.0214 |
|  | REF 42 |  |  |  | 96 | 283.12-393.17 | 0.1-99.965 | 0.1122 |
| [b4mpy][BF_4_] | REF 41 | 11 | 283.15-333.15 | 0.0050 | 143 | 283.15-333.15 | 0.1-65 | 0.0220 |
|  | REF 43 |  |  |  | 96 | 283.13-393.18 | 0.1-99.998 | 0.4903 |
| [C_4_mim][C_1_SO_4_] | REF 34 | 29 | 283.15-353.15 | 0.0094 | 232 | 283.15-353.15 | 0.1-35 | 0.0086 |
|  | REF 78 | 11 | 283.15-333.15 | 0.0062 | 88 | 283.15-333.15 | 0.1-35 | 0.0082 |
| [C_6_py][BF_4_] | REF 20 | 7 | 293.15-353.15 | 0.0059 | 28 | 293.15-353.15 | 0.1-20 | 0.0386 |
| [C_6_mim][BF_4_] | REF 12 | 4 | 293.15-353.15 | 0.0018 | 180 | 313.3-472.5 | 0.1-200 | 0.2269 |
|  | REF 13 | 9 | 283.15-323.15 | 0.0009 | 108 | 283.15-323.15 | 0.1-60 | 0.0093 |
|  | REF 14 |  |  |  | 67 | 219.916-357.125 | 0.1-61.164 | 0.0938 |
|  | REF 44 |  |  |  | 91 | 283.11-373.16 | 0.1-100.083 | 0.0822 |
| [C_2_mim][PF_6_] | REF 12 | 8 | 312.8-472.4 | 0.7881 | 182 | 312.8-472.4 | 10-200 | 1.7708 |
| [C_2_mim][OTf] | REF 14 |  |  |  | 61 | 264.143-347.765 | 0.1242-61.503 | 0.7039 |
|  | REF 45 | 7 | 293.15-393.15 | 0.0043 | 84 | 293.15-393.15 | 0.1-35 | 0.0072 |
| [C_8_py][BF_4_] | REF 20 | 7 | 293.15-353.15 | 0.0039 | 28 | 293.15-353.15 | 0.1-20 | 0.0283 |
| [C_8_mim][BF_4_] | REF 13 | 9 | 283.15-323.15 | 0.0013 | 108 | 283.15-323.15 | 0.1-60 | 0.0412 |
|  | REF 19 | 6 | 298.2-343.2 | 0.0282 | 13 | 298.2-323.2 | 0.1-206.94 | 0.1584 |
|  | REF 24 | 11 | 293.15-393.15 | 0.0065 | 66 | 293.15-393.15 | 0.1-10 | 0.0726 |
|  | REF 46 | 15 | 273.15-363.16 | 0.0022 | 54 | 298.15-348.15 | 0.1-224.2 | 0.1275 |
|  | REF 47 | 3 | 298.15-348.15 | 0.0000 | 24 | 298.15-348.15 | 0.1-196.1 | 0.1022 |
| [C_2_mim][Tos] | REF 17 | 12 | 318.15-428.15 | 0.0062 | 156 | 318.15-428.15 | 0.1-60 | 0.0490 |
| [C_4_mim][PF_6_] | REF 19 | 6 | 298.2-343.2 | 0.0136 | 12 | 298.2-323.2 | 0.1-202.11 | 0.1871 |
|  | REF 22 | 6 | 298.15-323.15 | 0.0001 | 138 | 298.15-323.15 | 0.1-100 | 0.0248 |
|  | REF 23 | 4 | 293.15-353.15 | 0.0149 | 16 | 293.15-353.15 | 0.1-20 | 0.0552 |
|  | REF 25 |  |  |  | 45 | 298.15-398.15 | 0.73-40.07 | 0.2734 |
|  | REF 26 | 10 | 293.49-414.93 | 0.0088 | 36 | 293.48-414.94 | 0.1-40 | 0.1879 |
|  | REF 29 | 9 | 312.8-472.3 | 0.0757 | 180 | 312.8-472.3 | 0.1-200 | 0.1980 |
|  | REF 79 | 3 | 294.9-335.1 | 0.0000 | 6 | 294.9-335.1 | 0.1-20 | 0.0956 |
| [C_4_mim][OTf] | REF 14 |  |  |  | 61 | 290.497-350.378 | 0.2411-60.046 | 0.1008 |
|  | REF 24 | 11 | 293.15-393.15 | 0.0125 | 66 | 293.15-393.15 | 0.1-10 | 0.0149 |
| [C_4_C_1_mim][PF_6_] | REF 24 | 9 | 313.15-393.15 | 0.0129 | 54 | 313.15-393.15 | 0.1-10 | 0.0175 |
| [C_6_mim][PF_6_] | REF 12 | 5 | 293.15-353.15 | 0.0031 | 180 | 312.9-472.3 | 0.1-200 | 0.3592 |
|  | REF 24 | 11 | 293.15-393.15 | 0.0062 | 66 | 293.15-393.15 | 0.1-10 | 0.0106 |
|  | REF 49 | 4 | 293.15-353.15 | 0.0157 | 16 | 293.15-353.15 | 0.1-20 | 0.0410 |
|  | REF 50 | 14 | 273.15-363.15 | 0.0021 | 32 | 298.15-348.15 | 0.1-238.5 | 0.1256 |
|  | REF 79 | 3 | 294.1-335.2 | 0.0000 | 6 | 294.1-335.2 | 0.1-20 | 0.0380 |
| [C_8_mim][PF_6_] | REF 12 | 4 | 293.15-353.15 | 0.0049 | 180 | 312.8-472.3 | 0.1-200 | 0.3137 |
|  | REF 19 | 6 | 298.2-343.2 | 0.0112 | 12 | 298.2-323.2 | 0.1-204.18 | 0.5762 |
|  | REF 24 | 11 | 293.15-393.15 | 0.0133 | 66 | 293.15-393.15 | 0.1-10 | 0.0287 |
|  | REF 46 | 14 | 273.15-363.16 | 0.0026 | 28 | 298.15-343.15 | 0.1-175.9 | 0.1247 |
|  | REF 47 | 33 | 273.2-353.17 | 0.0019 | 22 | 298.15-348.15 | 0.1-196.1 | 0.2651 |
|  | REF 49 | 4 | 293.15-353.15 | 0.0164 | 16 | 293.15-353.15 | 0.1-20 | 0.0728 |
|  | REF 79 | 3 | 295.1-335.2 | 0.0000 | 6 | 295.1-335.2 | 0.1-20 | 0.0404 |
| [C_4_mim][C_8_SO_4_] | REF 29 | 9 | 312.9-472.6 | 0.0613 | 169 | 312.9-472.6 | 0.1-200 | 0.5005 |
|  | REF 51 | 58 | 298.15-428.15 | 0.0689 | 156 | 318.15-428.15 | 0.1-60 | 0.2160 |
|  | REF 80 |  |  |  | 45 | 298.15-398.15 | 0.11-40.03 | 0.1364 |
| [C_2_mim][NTf_2_] | REF 11 | 8 | 293.15-393.15 | 0.0035 | 88 | 293.15-393.15 | 0.1-30 | 0.0273 |
|  | REF 26 | 10 | 293.49-414.92 | 0.0082 | 36 | 293.49-414.93 | 0.1-40 | 0.0077 |
|  | REF 52 |  |  |  | 84 | 283.19-373.18 | 0.1-100.087 | 0.1318 |
| [N_1114_][NTf_2_] | REF 53 | 10 | 293.48-414.93 | 0.0047 | 36 | 293.48-414.95 | 0.1-40 | 0.0260 |
| [C_3_mim][NTf_2_] | REF 54 | 15 | 298.15-333.15 | 0.0049 | 150 | 298.15-333.15 | 0.1-59.59 | 0.1234 |
| [C_3_mpyrro][NTf_2_] | REF 45 | 7 | 293.15-393.15 | 0.0023 | 84 | 293.15-393.15 | 0.1-35 | 0.0071 |
| [p3mpy][NTf_2_] | REF 45 | 7 | 293.15-393.15 | 0.0039 | 84 | 293.15-393.15 | 0.1-35 | 0.0133 |
| [C_4_mim][NTf_2_] | REF 15 | 14 | 293.15-473.15 | 0.0211 | 223 | 293.15-473.15 | 0.1-60.01 | 0.0252 |
|  | REF 26 | 6 | 293.49-414.92 | 0.0019 | 36 | 293.49-414.92 | 0.1-40 | 0.0332 |
|  | REF 50 | 14 | 273.15-363.15 | 0.0013 | 36 | 283.15-348.15 | 0.1-298.9 | 0.4570 |
|  | REF 55 | 3 | 298.15-348.15 | 0.0000 | 27 | 298.15-348.15 | 0.1-50 | 0.0389 |
|  | REF 56 | 7 | 298.15-328.2 | 0.0079 | 161 | 298.15-328.2 | 0.1-59.1 | 0.0454 |
|  | REF 81 | 16 | 273.15-363.15 | 0.0012 | 77 | 288.15-348.15 | 0.1-251.5 | 0.0630 |
|  | REF 82 | 9 | 293.15-373.15 | 0.0054 | 120 | 311.8-412.1 | 0.1-200 | 0.0812 |
| [C_4_mpyrro][NTf_2_] | REF 45 | 7 | 293.15-393.15 | 0.0021 | 84 | 293.15-393.15 | 0.1-35 | 0.0070 |
|  | REF 53 | 10 | 293.49-414.93 | 0.0017 | 36 | 293.5-414.94 | 0.1-40 | 0.0080 |
|  | REF 57 | 14 | 273.15-363.15 | 0.0026 | 54 | 273.15-348.15 | 0.1-102.92 | 0.1109 |
|  | REF 58 | 7 | 278.15-398.15 | 0.0376 | 56 | 278.15-398.15 | 0.1-120 | 0.0975 |
| [C_3_mpip][NTf_2_] | REF 45 | 7 | 293.15-393.15 | 0.0179 | 84 | 293.15-393.15 | 0.1-35 | 0.0202 |
|  | REF 59 | 3 | 298.15-348.15 | 0.0000 | 27 | 298.15-348.15 | 0.1-50 | 0.1150 |
| [C_1_OC_2_mpyrro][NTf_2_] | REF 37 | 14 | 278.15-398.15 | 0.0266 | 112 | 278.15-398.15 | 0.1-120 | 0.0348 |
| [C_5_mim][NTf_2_] | REF 54 | 15 | 298.15-333.15 | 0.0098 | 150 | 298.15-333.15 | 0.1-59.59 | 0.1055 |
| [Aliquat][DCA] | REF 15 | 11 | 293.15-413.15 | 0.0053 | 176 | 293.15-413.15 | 0.1-60 | 0.0147 |
| [C_6_mim][NTf_2_] | REF 56 | 15 | 298.15-333.15 | 0.0084 | 148 | 298.15-333.15 | 0.1-59.59 | 0.0826 |
|  | REF 60 | 4 | 298.15-373.15 | 0.0299 | 24 | 298.15-423.15 | 0.1-40 | 0.3019 |
|  | REF 61 | 13 | 293.15-338.15 | 0.0145 | 145 | 293.15-338.15 | 0.1-65.02 | 0.1410 |
|  | REF 62 | 8 | 293.15-452.3 | 0.0129 | 160 | 312.6-452.3 | 0.1-200 | 0.0687 |
|  | REF 63 | 10 | 273.15-413.15 | 0.0038 | 256 | 273.13-413.17 | 0.1-139.984 | 0.0541 |
|  | REF 81 | 14 | 273.15-363.15 | 0.0011 | 11 | 288.15 | 0.1-174.6 | 0.2091 |
| [C_7_mim][NTf_2_] | REF 11 | 8 | 293.15-393.15 | 0.0092 | 88 | 293.15-393.15 | 0.1-30 | 0.0343 |
| [C_8_mim][NTf_2_] | REF 11 | 8 | 293.15-393.15 | 0.0375 | 88 | 293.15-393.15 | 0.1-30 | 0.0574 |
|  | REF 64 |  |  |  | 60 | 293.22-343.13 | 0.101-25 | 0.1376 |
| [C_10_mim][NTf_2_] | REF 18 | 8 | 293.15-393.15 | 0.0292 | 72 | 293.15-393.15 | 0.1-35 | 0.0829 |
| [P_666 14_]Cl | REF 65 |  |  |  | 134 | 298.13-333.14 | 0.19-65 | 0.1282 |
|  | REF 66 | 8 | 273.15-318.15 | 0.0104 | 64 | 273.15-318.15 | 0.1-25 | 0.0216 |
|  | REF 67 | 6 | 283.15-333.15 | 0.0025 | 78 | 283.15-333.15 | 0.1-45 | 0.0276 |
| [P_666 14_][OAc] | REF 65 |  |  |  | 144 | 298.15-334.11 | 0.21-65.01 | 0.6099 |
| [P_666 14_][DCA] | REF 66 | 10 | 273.15-318.15 | 0.0194 | 90 | 273.15-318.15 | 0.1-35 | 0.0306 |
|  | REF 67 | 6 | 283.15-333.15 | 0.0032 | 78 | 283.15-333.15 | 0.1-45 | 0.0086 |
| [C_2_mim][(C_2_F_5_)_3_PF_3_] | REF 68 | 6 | 293.14-343.18 | 0.0013 | 48 | 293.13-343.16 | 0.1-25 | 0.0147 |
| [P_666 14_]Br | REF 67 | 6 | 283.15-333.15 | 0.0028 | 78 | 283.15-333.15 | 0.1-45 | 0.0500 |
| [P_666 14_][C_1_SO_3_] | REF 67 | 6 | 283.15-333.15 | 0.0031 | 78 | 283.15-333.15 | 0.1-45 | 0.0069 |
| [C_4_mim][(C_2_F_5_)_3_PF_3_] | REF 68 |  |  |  | 63 | 293.15-353.05 | 0.1-25 | 0.0219 |
| [C_4_mpyrro][(C_2_F_5_)_3_PF_3_] | REF 69 | 7 | 293.19-353.17 | 0.0031 | 56 | 293.17-353.19 | 0.1-25 | 0.0661 |
|  | REF 74 | 7 | 278.15-398.15 | 0.0076 | 56 | 278.15-398.15 | 0.1-120 | 0.0328 |
| [C_1_OC_2_mpyrro][(C_2_F_5_)_3_PF_3_] | REF 58 | 7 | 278.15-398.15 | 0.0135 | 56 | 278.15-398.15 | 0.1-120 | 0.0922 |
| [C_6_mim][(C_2_F_5_)_3_PF_3_] | REF 68 |  |  |  | 60 | 293.16-343.18 | 0.1-25 | 0.1603 |
| [P_666 14_][NTf_2_] | REF 65 |  |  |  | 126 | 298.15-333.43 | 0.21-65.01 | 0.0339 |
|  | REF 67 | 6 | 283.15-333.15 | 0.0027 | 78 | 283.15-333.15 | 0.1-45 | 0.0617 |
| [P_666 14_][(C_2_F_5_)_3_PF_3_] | REF 69 | 7 | 293.2-353.2 | 0.0050 | 56 | 293.21-353.25 | 0.1-25 | 0.0754 |
|  | REF 70 | 11 | 293.15-343.15 | 0.0014 | 77 | 293.15-343.15 | 0.1-25 | 0.0157 |
|  | REF 74 | 7 | 278.15-398.15 | 0.0102 | 56 | 278.15-398.15 | 0.1-120 | 0.0478 |
| [C_6_mim]Cl | REF 62 | 8 | 303.15-451.2 | 0.0223 | 160 | 311.5-451.2 | 0.1-200 | 0.0884 |
| [C_6_mim][OTf] | REF 14 |  |  |  | 48 | 303.139-351.769 | 0.1038-60.665 | 1.0437 |
| [C_4_C_1_mim][(C_2_F_5_)_3_PF_3_] | REF 72 | 14 | 278.15-398.15 | 0.0207 | 112 | 278.15-398.15 | 0.1-120 | 0.0406 |
| [C_4_C_1_mim][NTf_2_] | REF 72 | 14 | 278.15-398.15 | 0.0181 | 112 | 278.15-398.15 | 0.1-120 | 0.0404 |
| [C_2_OHC_1_NH_2_][C_2_COO] | REF 73 | 13 | 298.15-358.15 | 0.0001 | 91 | 298.15-358.15 | 0.1-25 | 0.1240 |
| [C_2_OHC_1_NH_2_][C_3_COO] | REF 73 | 7 | 298.15-358.15 | 0.0002 | 35 | 298.15-358.15 | 0.1-25 | 0.0143 |
| [C_2_OHC_1_NH_2_][C_4_COO] | REF 73 | 7 | 298.15-358.15 | 0.0001 | 35 | 298.15-358.15 | 0.1-25 | 0.0204 |
| [C_1_mim][DMP] | REF 74 | 7 | 278.15-398.15 | 0.0142 | 56 | 278.15-398.15 | 0.1-120 | 0.0501 |
| [C_2_mim][C_6_SO_4_] | REF 74 | 7 | 278.15-398.15 | 0.0139 | 56 | 278.15-398.15 | 0.1-120 | 0.0320 |
| [C_4_mpyrro][B(CN)_4_] | REF 74 | 6 | 298.15-398.15 | 0.0097 | 69 | 298.15-398.15 | 0.1-60 | 0.0308 |
| [C_4_mpyrro][OTf] | REF 74 | 7 | 278.15-398.15 | 0.0148 | 56 | 278.15-398.15 | 0.1-120 | 0.0442 |
| [C_3_py][BF_4_] | REF 75 | 11 | 283.15-333.15 | 0.0049 | 143 | 283.15-333.15 | 0.1-65 | 0.0273 |
| [C_4_py][OTf] | REF 75 | 7 | 303.15-333.15 | 0.0044 | 91 | 303.15-333.15 | 0.1-65 | 0.4412 |
| [b3mpy][DCA] | REF 75 | 9 | 293.15-333.15 | 0.0105 | 117 | 293.15-333.15 | 0.1-65 | 0.0189 |
| [o3mpy][BF_4_] | REF 75 | 11 | 283.15-333.15 | 0.0036 | 143 | 283.15-333.15 | 0.1-65 | 0.0220 |
| [amim]Cl | REF 77 | 9 | 293.15-373.15 | 0.0060 | 80 | 313.4-373 | 0.1-200 | 0.2971 |
| [C_2_mim][MP] | REF 82 | 9 | 293.15-373.15 | 0.0030 | 120 | 311.2-411.1 | 0.1-200 | 0.0985 |
| [C_2_mim][DEP] | REF 82 | 9 | 293.15-373.15 | 0.0059 | 120 | 311.4-411.7 | 0.1-200 | 0.0765 |
| [C_2_eim][NTf_2_] | REF 18 | 8 | 293.15-393.15 | 0.0113 | 72 | 293.15-393.15 | 0.1-35 | 0.0392 |

$$RAAD/\%=\frac{100}{N}\sum_{i=1}^{N} \left|\frac{\rho_{i}^{calc}-\rho_{i}^{\exp}}{\rho_{i}^{calc}}\right|$$

**Table S2. Relative Absolute Average Deviation (RAAD), Maximum Deviation (Dmax), and Average Deviation (Bias) for Densities of Ionic Liquids**

| **Ionic Liquid** | **Reference** | ***T* / K** | **p / MPa** | **Maximum deviation** | **RAAD/%** | **Dmax/%** | **Bias/%** | **Comments:**  **References for Isothermal Compressibility and Density at *p* = 0.1 MPa if differ from the references listed in column 2.** |
| --- | --- | --- | --- | --- | --- | --- | --- | --- |
| [Aliquat][DCA] | REF 15 | 293.15 | 0.1-60 | for  T = 413.15 K  and  p = 60 MPa  0.10 % | 0.0086 | 0.0185 | -0.0005 | Isothermal Compressibility from REF 15 |
|  |  | 303.15 | 0.1-60 |  | 0.0049 | 0.0124 | 0.0003 |  |
|  |  | 313.15 | 0.1-60 |  | 0.0074 | 0.0154 | 0.0047 |  |
|  |  | 323.15 | 0.1-60 |  | 0.0095 | 0.0237 | 0.0092 |  |
|  |  | 333.15 | 0.1-60 |  | 0.0116 | 0.0270 | 0.0113 |  |
|  |  | 343.15 | 0.1-60 |  | 0.0124 | 0.0356 | 0.0113 |  |
|  |  | 353.15 | 0.1-60 |  | 0.0140 | 0.0340 | 0.0140 |  |
|  |  | 363.15 | 0.1-60 |  | 0.0145 | 0.0430 | 0.0129 |  |
|  |  | 373.15 | 0.1-60 |  | 0.0146 | 0.0480 | 0.0095 |  |
|  |  | 393.15 | 0.1-60 |  | 0.0216 | 0.0640 | 0.0164 |  |
|  |  | 413.15 | 0.1-60 |  | 0.0358 | 0.0964 | 0.0358 |  |
| [amim]Cl | REF 77 | 293.15 | 0.1 | for  T = 373 K  and  p = 150 MPa  0.51 % | 0.0053 | 0.0053 | 0.0053 | Isothermal Compressibility calculated from Tait equation – REF 77 |
|  |  | 303.15 | 0.1 |  | 0.0089 | 0.0089 | -0.0089 |  |
|  |  | 313.15 | 0.1 |  | 0.0040 | 0.0040 | -0.0040 |  |
|  |  | 323.15 | 0.1 |  | 0.0113 | 0.0113 | 0.0113 |  |
|  |  | 333.15 | 0.1 |  | 0.0017 | 0.0017 | 0.0017 |  |
|  |  | 343.15 | 0.1 |  | 0.0065 | 0.0065 | -0.0065 |  |
|  |  | 353.15 | 0.1 |  | 0.0041 | 0.0041 | -0.0041 |  |
|  |  | 363.15 | 0.1 |  | 0.0089 | 0.0089 | 0.0089 |  |
|  |  | 373.15 | 0.1 |  | 0.0036 | 0.0036 | -0.0036 |  |
|  |  | 313.4 | 10-200 |  | 0.1990 | 0.2561 | 0.1990 |  |
|  |  | 332.7 | 10-200 |  | 0.2611 | 0.3281 | 0.2611 |  |
|  |  | 352.8 | 10-200 |  | 0.3229 | 0.4318 | 0.3229 |  |
|  |  | 373 | 10-200 |  | 0.4013 | 0.5043 | 0.4013 |  |
| [b2mpy][BF_4_] | REF 41 | 293.15 | 0.1-45 | for  T = 308.15 K  and  p = 60 MPa  0.06 % | 0.0147 | 0.0372 | 0.0123 | Isothermal Compressibility from REF 41 |
|  |  | 298.15 | 0.1-55 |  | 0.0176 | 0.0480 | 0.0139 |  |
|  |  | 303.15 | 0.1-65 |  | 0.0190 | 0.0547 | 0.0175 |  |
|  |  | 308.15 | 0.1-65 |  | 0.0324 | 0.0567 | 0.0324 |  |
|  |  | 313.15 | 0.1-65 |  | 0.0114 | 0.0439 | 0.0104 |  |
|  |  | 318.15 | 0.1-65 |  | 0.0214 | 0.0416 | 0.0214 |  |
|  |  | 323.15 | 0.1-65 |  | 0.0058 | 0.0270 | 0.0030 |  |
|  |  | 328.15 | 0.1-65 |  | 0.0108 | 0.0283 | 0.0107 |  |
|  |  | 333.15 | 0.1-65 |  | 0.0085 | 0.0195 | -0.0015 |  |
| [b3mpy][BF_4_] | REF 41 | 283.15 | 0.1-65 | for  T = 288.15 K  and  p = 65 MPa  0.08 % | 0.0231 | 0.0773 | 0.0187 | Isothermal Compressibility from REF 41 |
|  |  | 288.15 | 0.1-65 |  | 0.0338 | 0.0796 | 0.0338 |  |
|  |  | 293.15 | 0.1-65 |  | 0.0170 | 0.0518 | 0.0133 |  |
|  |  | 298.15 | 0.1-65 |  | 0.0249 | 0.0597 | 0.0248 |  |
|  |  | 303.15 | 0.1-65 |  | 0.0172 | 0.0624 | 0.0143 |  |
|  |  | 308.15 | 0.1-65 |  | 0.0205 | 0.0514 | 0.0205 |  |
|  |  | 313.15 | 0.1-65 |  | 0.0142 | 0.0433 | 0.0068 |  |
|  |  | 318.15 | 0.1-65 |  | 0.0233 | 0.0465 | 0.0233 |  |
|  |  | 323.15 | 0.1-65 |  | 0.0115 | 0.0360 | 0.0067 |  |
|  |  | 328.15 | 0.1-65 |  | 0.0240 | 0.0494 | 0.0240 |  |
|  |  | 333.15 | 0.1-65 |  | 0.0115 | 0.0347 | 0.0098 |  |
| [b3mpy][BF_4_] | REF 42 | 283.12 | 2.024 | for  T = 293.15 K  and  p = 30.013 MPa  0.40 % | 0.0254 | 0.0254 | -0.0254 | Isothermal Compressibility from REF 41, density (p = 0.1 MPa) from REF 41 |
|  |  | 283.13 | 5.41 |  | 0.0232 | 0.0232 | -0.0232 |  |
|  |  | 283.14 | 10.608 |  | 0.0208 | 0.0208 | -0.0208 |  |
|  |  | 283.16 | 20.395-99.226 |  | 0.0243 | 0.0304 | -0.0243 |  |
|  |  | 293.13 | 2.373 |  | 0.0452 | 0.0452 | -0.0452 |  |
|  |  | 293.15 | 5.192-99.088 |  | 0.0642 | 0.0873 | -0.0642 |  |
|  |  | 293.16 | 79.917 |  | 0.0821 | 0.0821 | -0.0821 |  |
|  |  | 293.17 | 10.105 |  | 0.0459 | 0.0459 | -0.0459 |  |
|  |  | 298.15 | 1.023-99.965 |  | 0.0765 | 0.1093 | -0.0765 |  |
|  |  | 313.13 | 10.051 |  | 0.0568 | 0.0568 | -0.0568 |  |
|  |  | 313.15 | 2.471-79.83 |  | 0.0969 | 0.1362 | -0.0969 |  |
|  |  | 313.16 | 30.021-89.989 |  | 0.1107 | 0.1446 | -0.1107 |  |
|  |  | 313.17 | 19.949-99.194 |  | 0.1079 | 0.1495 | -0.1079 |  |
|  |  | 333.13 | 2.503 |  | 0.0234 | 0.0234 | -0.0234 |  |
|  |  | 333.14 | 39.76 |  | 0.0617 | 0.0617 | -0.0617 |  |
|  |  | 333.15 | 20.056-99.773 |  | 0.0950 | 0.1377 | -0.0950 |  |
|  |  | 333.16 | 5.448-70.003 |  | 0.0721 | 0.1104 | -0.0721 |  |
|  |  | 333.17 | 10.715-60.243 |  | 0.0625 | 0.0965 | -0.0625 |  |
|  |  | 353.12 | 6.509 |  | 0.0518 | 0.0518 | 0.0518 |  |
|  |  | 353.13 | 8.955 |  | 0.0522 | 0.0522 | 0.0522 |  |
|  |  | 353.14 | 2.79 |  | 0.0485 | 0.0485 | 0.0485 |  |
|  |  | 353.15 | 90.011-99.259 |  | 0.0485 | 0.0511 | -0.0485 |  |
|  |  | 353.16 | 29.979-80.426 |  | 0.0231 | 0.0378 | -0.0006 |  |
|  |  | 353.17 | 21.342 |  | 0.0460 | 0.0460 | 0.0460 |  |
|  |  | 373.12 | 60.192 |  | 0.1502 | 0.1502 | 0.1502 |  |
|  |  | 373.13 | 50.003 |  | 0.1636 | 0.1636 | 0.1636 |  |
|  |  | 373.14 | 39.996-99.035 |  | 0.1422 | 0.1760 | 0.1422 |  |
|  |  | 373.15 | 5.373-90.014 |  | 0.1573 | 0.1847 | 0.1573 |  |
|  |  | 373.16 | 2.495-80.221 |  | 0.1631 | 0.1871 | 0.1631 |  |
|  |  | 393.15 | 10.222-99.831 |  | 0.3728 | 0.3980 | 0.3728 |  |
|  |  | 393.16 | 40.133-80.159 |  | 0.3806 | 0.3980 | 0.3806 |  |
|  |  | 393.17 | 2.485-5.3 |  | 0.3317 | 0.3390 | 0.3317 |  |
| [b3mpy][DCA] | REF 75 | 293.15 | 0.1-65 | for  T = 303.15 K  and  p = 65 MPa  0.06 % | 0.0208 | 0.0550 | 0.0177 | Isothermal Compressibility from REF 75 |
|  |  | 298.15 | 0.1-65 |  | 0.0199 | 0.0506 | 0.0198 |  |
|  |  | 303.15 | 0.1-65 |  | 0.0276 | 0.0565 | 0.0276 |  |
|  |  | 308.15 | 0.1-65 |  | 0.0216 | 0.0519 | 0.0216 |  |
|  |  | 313.15 | 0.1-65 |  | 0.0166 | 0.0433 | 0.0166 |  |
|  |  | 318.15 | 0.1-65 |  | 0.0187 | 0.0379 | 0.0175 |  |
|  |  | 323.15 | 0.1-65 |  | 0.0141 | 0.0341 | 0.0044 |  |
|  |  | 328.15 | 0.1-65 |  | 0.0112 | 0.0256 | 0.0041 |  |
|  |  | 333.15 | 0.1-65 |  | 0.0139 | 0.0349 | 0.0030 |  |
| [b4mpy][BF_4_] | REF 41 | 283.15 | 0.1-65 | for  T = 283.15 K  and  p = 65 MPa  0.08 % | 0.0285 | 0.0833 | 0.0260 | Isothermal Compressibility from REF 41 |
|  |  | 288.15 | 0.1-65 |  | 0.0314 | 0.0751 | 0.0309 |  |
|  |  | 293.15 | 0.1-65 |  | 0.0175 | 0.0569 | 0.0143 |  |
|  |  | 298.15 | 0.1-65 |  | 0.0253 | 0.0609 | 0.0253 |  |
|  |  | 303.15 | 0.1-65 |  | 0.0152 | 0.0417 | 0.0124 |  |
|  |  | 308.15 | 0.1-65 |  | 0.0286 | 0.0550 | 0.0286 |  |
|  |  | 313.15 | 0.1-65 |  | 0.0125 | 0.0465 | 0.0094 |  |
|  |  | 318.15 | 0.1-65 |  | 0.0213 | 0.0581 | 0.0213 |  |
|  |  | 323.15 | 0.1-65 |  | 0.0117 | 0.0298 | 0.0023 |  |
|  |  | 328.15 | 0.1-65 |  | 0.0233 | 0.0482 | 0.0233 |  |
|  |  | 333.15 | 0.1-65 |  | 0.0132 | 0.0506 | 0.0121 |  |
| [b4mpy][BF_4_] | REF 43 | 283.13 | 2.389 | for  T = 313.16 K  and  p = 99.473 MPa  0.65 % | 0.5421 | 0.5421 | -0.5421 | Isothermal Compressibility from REF 41, density (p = 0.1 MPa) from REF 41 |
|  |  | 283.14 | 99.418 |  | 0.5279 | 0.5279 | -0.5279 |  |
|  |  | 283.15 | 60.042-89.923 |  | 0.5249 | 0.5272 | -0.5249 |  |
|  |  | 283.16 | 49.953-69.924 |  | 0.5227 | 0.5240 | -0.5227 |  |
|  |  | 283.17 | 20.156-39.808 |  | 0.5245 | 0.5281 | -0.5245 |  |
|  |  | 283.2 | 5.533-10.148 |  | 0.5397 | 0.5422 | -0.5397 |  |
|  |  | 293.13 | 2.593 |  | 0.5629 | 0.5629 | -0.5629 |  |
|  |  | 293.14 | 99.354 |  | 0.5878 | 0.5878 | -0.5878 |  |
|  |  | 293.15 | 69.958-90.001 |  | 0.5801 | 0.5854 | -0.5801 |  |
|  |  | 293.16 | 5.22-59.929 |  | 0.5649 | 0.5687 | -0.5649 |  |
|  |  | 293.17 | 10.236-39.693 |  | 0.5582 | 0.5596 | -0.5582 |  |
|  |  | 298.15 | 1.003-90.002 |  | 0.5831 | 0.6128 | -0.5831 |  |
|  |  | 313.15 | 59.74 |  | 0.6169 | 0.6169 | -0.6169 |  |
|  |  | 313.16 | 2.707-99.473 |  | 0.6116 | 0.6571 | -0.6116 |  |
|  |  | 313.17 | 5.339-79.71 |  | 0.5969 | 0.6403 | -0.5969 |  |
|  |  | 333.13 | 1.547-39.448 |  | 0.5614 | 0.5734 | -0.5614 |  |
|  |  | 333.14 | 49.914-60.043 |  | 0.5971 | 0.6051 | -0.5971 |  |
|  |  | 333.15 | 5.146-69.942 |  | 0.5771 | 0.6200 | -0.5771 |  |
|  |  | 333.16 | 19.866-89.996 |  | 0.6109 | 0.6459 | -0.6109 |  |
|  |  | 333.17 | 9.837-99.595 |  | 0.6015 | 0.6536 | -0.6015 |  |
|  |  | 353.13 | 59.133 |  | 0.5260 | 0.5260 | -0.5260 |  |
|  |  | 353.14 | 2.498-70.001 |  | 0.4941 | 0.5441 | -0.4941 |  |
|  |  | 353.15 | 89.993 |  | 0.5675 | 0.5675 | -0.5675 |  |
|  |  | 353.16 | 79.945-98.529 |  | 0.5659 | 0.5735 | -0.5659 |  |
|  |  | 373.13 | 59.93 |  | 0.3822 | 0.3822 | -0.3822 |  |
|  |  | 373.14 | 2.866-69.984 |  | 0.3728 | 0.3959 | -0.3728 |  |
|  |  | 373.15 | 5.031-99.124 |  | 0.3900 | 0.4157 | -0.3900 |  |
|  |  | 373.16 | 9.772 |  | 0.3562 | 0.3562 | -0.3562 |  |
|  |  | 373.17 | 20.168 |  | 0.3467 | 0.3467 | -0.3467 |  |
|  |  | 393.14 | 99.382 |  | 0.1782 | 0.1782 | -0.1782 |  |
|  |  | 393.15 | 10.459-90.001 |  | 0.1728 | 0.1832 | -0.1728 |  |
|  |  | 393.16 | 2.068-29.942 |  | 0.1898 | 0.2207 | -0.1898 |  |
|  |  | 393.17 | 39.803-80.173 |  | 0.1645 | 0.1822 | -0.1645 |  |
|  |  | 393.18 | 59.764 |  | 0.1648 | 0.1648 | -0.1648 |  |
| [b4mpy][SCN] | REF 6 | 298.15 | 0.1-10 | for  T = 328.15 K  and  p = 0.6 MPa  0.05 % | 0.0171 | 0.0196 | -0.0171 | Isothermal Compressibility from REF 6 |
|  |  | 308.15 | 0.1-10 |  | 0.0338 | 0.0380 | 0.0338 |  |
|  |  | 318.15 | 0.1-10 |  | 0.0016 | 0.0047 | 0.0014 |  |
|  |  | 328.15 | 0.1-10 |  | 0.0437 | 0.0453 | -0.0437 |  |
|  |  | 338.15 | 0.1-10 |  | 0.0253 | 0.0273 | 0.0253 |  |
| [C_10_mim][NTf_2_] | REF 18 | 293.15 | 0.1-35 | for  T = 373.15 K  and  p = 35 MPa  0.28 % | 0.0377 | 0.0768 | -0.0105 | Isothermal Compressibility from REF 18 |
|  |  | 298.15 | 0.1-35 |  | 0.0374 | 0.0776 | 0.0099 |  |
|  |  | 303.15 | 0.1-35 |  | 0.0368 | 0.0764 | 0.0032 |  |
|  |  | 313.15 | 0.1-35 |  | 0.0529 | 0.1134 | 0.0149 |  |
|  |  | 333.15 | 0.1-35 |  | 0.1583 | 0.2774 | 0.1583 |  |
|  |  | 353.15 | 0.1-35 |  | 0.0823 | 0.2102 | 0.0686 |  |
|  |  | 373.15 | 0.1-35 |  | 0.1232 | 0.2799 | 0.1232 |  |
|  |  | 393.15 | 0.1-35 |  | 0.0908 | 0.2223 | 0.0356 |  |
| [C_1_mim][DMP] | REF 74 | 278.15 | 0.1-120 | for  T = 298.15 K  and  p = 120 MPa  0.17 % | 0.0702 | 0.1597 | -0.0670 | Isothermal Compressibility and density calculated from Tait equation from REF 74 |
|  |  | 298.15 | 0.1-120 |  | 0.0626 | 0.1678 | -0.0315 |  |
|  |  | 313.15 | 0.1-120 |  | 0.0521 | 0.1436 | -0.0313 |  |
|  |  | 333.15 | 0.1-120 |  | 0.0411 | 0.1298 | -0.0370 |  |
|  |  | 348.15 | 0.1-120 |  | 0.0480 | 0.1257 | -0.0480 |  |
|  |  | 373.15 | 0.1-120 |  | 0.0386 | 0.0858 | -0.0379 |  |
|  |  | 398.15 | 0.1-120 |  | 0.0104 | 0.0212 | -0.0064 |  |
| [C_1_mim][C_1_SO_4_] | REF 16 | 313.15 | 2.5-25 | for  T = 328.15 K  and  p = 25 MPa  0.12 % | 0.0557 | 0.1224 | 0.0542 | Isothermal Compressibility from REF 17 |
|  |  | 318.15 | 0.1-25 |  | 0.0360 | 0.0723 | -0.0263 |  |
|  |  | 323.15 | 0.1-25 |  | 0.0628 | 0.1144 | -0.0592 |  |
|  |  | 328.15 | 0.1-25 |  | 0.0637 | 0.1232 | -0.0598 |  |
|  |  | 333.15 | 0.1-25 |  | 0.0359 | 0.0874 | -0.0293 |  |
| [C_1_mim][C_1_SO_4_] | REF 17 | 318.15 | 0.1-60 | for  T = 358.15 K  and  p = 50 MPa  0.15 % | 0.0431 | 0.0714 | 0.0392 | Isothermal Compressibility from REF 17 |
|  |  | 328.15 | 0.1-60 |  | 0.0619 | 0.0978 | 0.0590 |  |
|  |  | 338.15 | 0.1-60 |  | 0.0934 | 0.1328 | 0.0934 |  |
|  |  | 348.15 | 0.1-60 |  | 0.0999 | 0.1383 | 0.0999 |  |
|  |  | 358.15 | 0.1-60 |  | 0.1028 | 0.1506 | 0.1028 |  |
|  |  | 368.15 | 0.1-60 |  | 0.0944 | 0.1457 | 0.0944 |  |
|  |  | 378.15 | 0.1-60 |  | 0.0832 | 0.1343 | 0.0817 |  |
|  |  | 388.15 | 0.1-60 |  | 0.0704 | 0.1152 | 0.0669 |  |
|  |  | 398.15 | 0.1-60 |  | 0.0487 | 0.0838 | 0.0308 |  |
|  |  | 408.15 | 0.1-60 |  | 0.0582 | 0.0945 | 0.0582 |  |
|  |  | 418.15 | 0.1-60 |  | 0.0720 | 0.0912 | 0.0720 |  |
|  |  | 428.15 | 0.1-60 |  | 0.0303 | 0.0481 | 0.0268 |  |
| [C_1_OC_2_mPyrro][(C_2_F_5_)_3_PF_3_] | REF 58 | 278.15 | 0.1-120 | for  T = 298.15 K  and  p = 120 MPa  0.18 % | 0.0870 | 0.1725 | -0.0870 | Isothermal Compressibility calculated from Tait equation from REF 58 |
|  |  | 298.15 | 0.1-120 |  | 0.0692 | 0.1802 | -0.0608 |  |
|  |  | 313.15 | 0.1-120 |  | 0.0750 | 0.1620 | -0.0706 |  |
|  |  | 333.15 | 0.1-120 |  | 0.0861 | 0.1570 | -0.0861 |  |
|  |  | 348.15 | 0.1-120 |  | 0.0970 | 0.1689 | -0.0970 |  |
|  |  | 373.15 | 0.1-120 |  | 0.0993 | 0.1617 | -0.0986 |  |
|  |  | 398.15 | 0.1-120 |  | 0.0711 | 0.1073 | -0.0498 |  |
| [C_1_OC_2_mPyrro][NTf_2_] | REF 37 | 278.15 | 0.1-120 | for  T = 278.15 K  and  p = 120 MPa  0.18 % | 0.0435 | 0.1043 | -0.0435 | Isothermal Compressibility calculated from Tait equation from REF 37 |
|  |  | 298.15 | 0.1-120 |  | 0.0410 | 0.0599 | 0.0397 |  |
|  |  | 313.15 | 0.1-120 |  | 0.0202 | 0.0435 | 0.0153 |  |
|  |  | 333.15 | 0.1-120 |  | 0.0056 | 0.0205 | -0.0021 |  |
|  |  | 348.15 | 0.1-120 |  | 0.0192 | 0.0287 | -0.0192 |  |
|  |  | 373.15 | 0.1-120 |  | 0.0151 | 0.0315 | 0.0077 |  |
|  |  | 398.15 | 0.1-120 |  | 0.0577 | 0.0862 | 0.0577 |  |
|  |  | 278.15 | 0.1-120 |  | 0.1086 | 0.1759 | -0.1086 |  |
|  |  | 298.15 | 0.1-120 |  | 0.0236 | 0.0650 | -0.0029 |  |
|  |  | 313.15 | 0.1-120 |  | 0.0192 | 0.0660 | -0.0156 |  |
|  |  | 333.15 | 0.1-120 |  | 0.0250 | 0.0540 | -0.0250 |  |
|  |  | 348.15 | 0.1-120 |  | 0.0361 | 0.0556 | -0.0361 |  |
|  |  | 373.15 | 0.1-120 |  | 0.0132 | 0.0427 | -0.0040 |  |
|  |  | 398.15 | 0.1-120 |  | 0.0466 | 0.0789 | 0.0466 |  |
| [C_2_eim][NTf_2_] | REF 18 | 293.15 | 0.1-35 | for  T = 393.15 K  and  p = 35 MPa  0.15 % | 0.0226 | 0.0479 | 0.0166 | Isothermal Compressibility from REF 18 |
|  |  | 298.15 | 0.1-35 |  | 0.0306 | 0.0802 | 0.0296 |  |
|  |  | 303.15 | 0.1-35 |  | 0.0374 | 0.0895 | 0.0361 |  |
|  |  | 313.15 | 0.1-35 |  | 0.0304 | 0.0682 | 0.0130 |  |
|  |  | 333.15 | 0.1-35 |  | 0.0290 | 0.0823 | 0.0188 |  |
|  |  | 353.15 | 0.1-35 |  | 0.0357 | 0.0961 | 0.0216 |  |
|  |  | 373.15 | 0.1-35 |  | 0.0498 | 0.1310 | 0.0466 |  |
|  |  | 393.15 | 0.1-35 |  | 0.0552 | 0.1522 | 0.0493 |  |
| [C_2_mim][(C_2_F_5_)_3_PF_3_] | REF 68 | 293.14 | 0.1-25 | for  T = 293.13 K  and  p = 25 MPa  0.04 % | 0.0006 | 0.0006 | -0.0006 | Isothermal Compressibility calculated by using the GCM method proposed in REF 53 |
|  |  | 303.16 | 0.1-25 |  | 0.0018 | 0.0018 | 0.0018 |  |
|  |  | 313.15 | 0.1-25 |  | 0.0012 | 0.0012 | -0.0012 |  |
|  |  | 323.16 | 0.1-25 |  | 0.0014 | 0.0014 | -0.0014 |  |
|  |  | 333.18 | 0.1-25 |  | 0.0021 | 0.0021 | 0.0021 |  |
|  |  | 343.18 | 0.1-25 |  | 0.0007 | 0.0007 | -0.0007 |  |
|  |  | 293.13 | 0.1-25 |  | 0.0043 | 0.0043 | 0.0043 |  |
|  |  | 303.17 | 0.1-25 |  | 0.0001 | 0.0001 | 0.0001 |  |
|  |  | 313.17 | 0.1-25 |  | 0.0030 | 0.0030 | -0.0030 |  |
|  |  | 323.14 | 0.1-25 |  | 0.0117 | 0.0117 | -0.0117 |  |
|  |  | 333.17 | 0.1-25 |  | 0.0084 | 0.0084 | -0.0084 |  |
|  |  | 343.13 | 0.1-25 |  | 0.0198 | 0.0198 | -0.0198 |  |
|  |  | 293.13 | 0.1-25 |  | 0.0022 | 0.0022 | 0.0022 |  |
|  |  | 303.18 | 0.1-25 |  | 0.0020 | 0.0020 | -0.0020 |  |
|  |  | 313.18 | 0.1-25 |  | 0.0043 | 0.0043 | -0.0043 |  |
|  |  | 323.13 | 0.1-25 |  | 0.0107 | 0.0107 | -0.0107 |  |
|  |  | 333.18 | 0.1-25 |  | 0.0080 | 0.0080 | -0.0080 |  |
|  |  | 343.14 | 0.1-25 |  | 0.0187 | 0.0187 | -0.0187 |  |
|  |  | 293.14 | 0.1-25 |  | 0.0123 | 0.0123 | 0.0123 |  |
|  |  | 303.18 | 0.1-25 |  | 0.0112 | 0.0112 | 0.0112 |  |
|  |  | 313.18 | 0.1-25 |  | 0.0005 | 0.0005 | -0.0005 |  |
|  |  | 323.15 | 0.1-25 |  | 0.0119 | 0.0119 | -0.0119 |  |
|  |  | 333.18 | 0.1-25 |  | 0.0054 | 0.0054 | -0.0054 |  |
|  |  | 343.16 | 0.1-25 |  | 0.0212 | 0.0212 | -0.0212 |  |
|  |  | 293.14 | 0.1-25 |  | 0.0114 | 0.0114 | 0.0114 |  |
|  |  | 303.19 | 0.1-25 |  | 0.0135 | 0.0135 | 0.0135 |  |
|  |  | 313.17 | 0.1-25 |  | 0.0012 | 0.0012 | 0.0012 |  |
|  |  | 323.14 | 0.1-25 |  | 0.0122 | 0.0122 | -0.0122 |  |
|  |  | 333.18 | 0.1-25 |  | 0.0086 | 0.0086 | -0.0086 |  |
|  |  | 343.13 | 0.1-25 |  | 0.0243 | 0.0243 | -0.0243 |  |
|  |  | 293.14 | 0.1-25 |  | 0.0226 | 0.0226 | 0.0226 |  |
|  |  | 303.22 | 0.1-25 |  | 0.0185 | 0.0185 | 0.0185 |  |
|  |  | 313.18 | 0.1-25 |  | 0.0093 | 0.0093 | 0.0093 |  |
|  |  | 323.13 | 0.1-25 |  | 0.0070 | 0.0070 | -0.0070 |  |
|  |  | 333.18 | 0.1-25 |  | 0.0082 | 0.0082 | -0.0082 |  |
|  |  | 343.14 | 0.1-25 |  | 0.0290 | 0.0290 | -0.0290 |  |
|  |  | 293.13 | 0.1-25 |  | 0.0284 | 0.0284 | 0.0284 |  |
|  |  | 303.19 | 0.1-25 |  | 0.0214 | 0.0214 | 0.0214 |  |
|  |  | 313.17 | 0.1-25 |  | 0.0123 | 0.0123 | 0.0123 |  |
|  |  | 323.14 | 0.1-25 |  | 0.0038 | 0.0038 | -0.0038 |  |
|  |  | 333.19 | 0.1-25 |  | 0.0095 | 0.0095 | -0.0095 |  |
|  |  | 343.13 | 0.1-25 |  | 0.0335 | 0.0335 | -0.0335 |  |
|  |  | 293.13 | 0.1-25 |  | 0.0393 | 0.0393 | 0.0393 |  |
|  |  | 303.18 | 0.1-25 |  | 0.0287 | 0.0287 | 0.0287 |  |
|  |  | 313.16 | 0.1-25 |  | 0.0209 | 0.0209 | 0.0209 |  |
|  |  | 323.13 | 0.1-25 |  | 0.0003 | 0.0003 | 0.0003 |  |
|  |  | 333.17 | 0.1-25 |  | 0.0093 | 0.0093 | -0.0093 |  |
|  |  | 343.13 | 0.1-25 |  | 0.0274 | 0.0274 | -0.0274 |  |
|  |  | 293.13 | 0.1-25 |  | 0.0449 | 0.0449 | 0.0449 |  |
|  |  | 303.19 | 0.1-25 |  | 0.0405 | 0.0405 | 0.0405 |  |
|  |  | 313.17 | 0.1-25 |  | 0.0281 | 0.0281 | 0.0281 |  |
|  |  | 323.13 | 0.1-25 |  | 0.0094 | 0.0094 | 0.0094 |  |
|  |  | 333.18 | 0.1-25 |  | 0.0003 | 0.0003 | 0.0003 |  |
|  |  | 343.13 | 0.1-25 |  | 0.0278 | 0.0278 | -0.0278 |  |
| [C_2_mim][DCA] | REF 9, 10 | 256.825 | 20.357 | for  T = 339.672 K  and  p = 1.074 MPa  0.45 % | 0.2440 | 0.2440 | -0.2440 | Density from REF 10, Isothermal Compressibility calculated using the GCM method proposed in REF 53 |
|  |  | 256.923 | 29.975 |  | 0.2139 | 0.2139 | -0.2139 |  |
|  |  | 256.974 | 10.611 |  | 0.2564 | 0.2564 | -0.2564 |  |
|  |  | 257.123 | 1.2508 |  | 0.2736 | 0.2736 | -0.2736 |  |
|  |  | 261.968 | 9.9502 |  | 0.2778 | 0.2778 | -0.2778 |  |
|  |  | 262.109 | 19.886 |  | 0.2621 | 0.2621 | -0.2621 |  |
|  |  | 262.332 | 39.98 |  | 0.2208 | 0.2208 | -0.2208 |  |
|  |  | 262.456 | 0.6196 |  | 0.2936 | 0.2936 | -0.2936 |  |
|  |  | 267.671 | 20.023-39.943 |  | 0.2720 | 0.2837 | -0.2720 |  |
|  |  | 267.697 | 29.879 |  | 0.2685 | 0.2685 | -0.2685 |  |
|  |  | 267.701 | 49.706 |  | 0.2296 | 0.2296 | -0.2296 |  |
|  |  | 267.704 | 9.7532-9.894 |  | 0.3024 | 0.3108 | -0.3024 |  |
|  |  | 267.705 | 29.968 |  | 0.2664 | 0.2664 | -0.2664 |  |
|  |  | 267.706 | 49.662 |  | 0.2310 | 0.2310 | -0.2310 |  |
|  |  | 267.708 | 20.196 |  | 0.2799 | 0.2799 | -0.2799 |  |
|  |  | 267.711 | 40.066 |  | 0.2593 | 0.2593 | -0.2593 |  |
|  |  | 268.056 | 0.6497 |  | 0.3124 | 0.3124 | -0.3124 |  |
|  |  | 273.249 | 29.805 |  | 0.2868 | 0.2868 | -0.2868 |  |
|  |  | 273.343 | 49.967 |  | 0.2704 | 0.2704 | -0.2704 |  |
|  |  | 273.4 | 39.951 |  | 0.2740 | 0.2740 | -0.2740 |  |
|  |  | 273.491 | 19.943 |  | 0.2970 | 0.2970 | -0.2970 |  |
|  |  | 273.51 | 10.041 |  | 0.3120 | 0.3120 | -0.3120 |  |
|  |  | 273.552 | 60.051 |  | 0.2439 | 0.2439 | -0.2439 |  |
|  |  | 274.207 | 0.7757 |  | 0.3302 | 0.3302 | -0.3302 |  |
|  |  | 278.57 | 59.071 |  | 0.2791 | 0.2791 | -0.2791 |  |
|  |  | 279.197 | 40.05 |  | 0.2927 | 0.2927 | -0.2927 |  |
|  |  | 279.22 | 50.024 |  | 0.2814 | 0.2814 | -0.2814 |  |
|  |  | 279.413 | 30.01 |  | 0.3017 | 0.3017 | -0.3017 |  |
|  |  | 279.594 | 10.349 |  | 0.3232 | 0.3232 | -0.3232 |  |
|  |  | 279.65 | 9.9606 |  | 0.3277 | 0.3277 | -0.3277 |  |
|  |  | 279.661 | 20.569 |  | 0.3104 | 0.3104 | -0.3104 |  |
|  |  | 280.013 | 1.0168 |  | 0.3445 | 0.3445 | -0.3445 |  |
|  |  | 280.04 | 1.0776 |  | 0.3442 | 0.3442 | -0.3442 |  |
|  |  | 285.078 | 50.002 |  | 0.2989 | 0.2989 | -0.2989 |  |
|  |  | 285.128 | 60.064 |  | 0.2906 | 0.2906 | -0.2906 |  |
|  |  | 285.391 | 9.8165 |  | 0.3432 | 0.3432 | -0.3432 |  |
|  |  | 285.415 | 40.116 |  | 0.3057 | 0.3057 | -0.3057 |  |
|  |  | 285.434 | 19.56 |  | 0.3263 | 0.3263 | -0.3263 |  |
|  |  | 285.437 | 19.459 |  | 0.3297 | 0.3297 | -0.3297 |  |
|  |  | 285.452 | 9.8149 |  | 0.3470 | 0.3470 | -0.3470 |  |
|  |  | 285.503 | 20.153 |  | 0.3222 | 0.3222 | -0.3222 |  |
|  |  | 285.507 | 30.235 |  | 0.3149 | 0.3149 | -0.3149 |  |
|  |  | 286.615 | 2.009 |  | 0.3609 | 0.3609 | -0.3609 |  |
|  |  | 291.086 | 59.995 |  | 0.3059 | 0.3059 | -0.3059 |  |
|  |  | 291.453 | 50.059 |  | 0.3114 | 0.3114 | -0.3114 |  |
|  |  | 291.467 | 9.754 |  | 0.3588 | 0.3588 | -0.3588 |  |
|  |  | 291.47 | 50.178 |  | 0.3195 | 0.3195 | -0.3195 |  |
|  |  | 291.509 | 40.153 |  | 0.3156 | 0.3156 | -0.3156 |  |
|  |  | 291.613 | 19.904 |  | 0.3405 | 0.3405 | -0.3405 |  |
|  |  | 291.792 | 29.944 |  | 0.3264 | 0.3264 | -0.3264 |  |
|  |  | 297.625 | 60.101 |  | 0.3163 | 0.3163 | -0.3163 |  |
|  |  | 297.754 | 50.232 |  | 0.3192 | 0.3192 | -0.3192 |  |
|  |  | 297.911 | 20.011 |  | 0.3529 | 0.3529 | -0.3529 |  |
|  |  | 297.976 | 30.141 |  | 0.3359 | 0.3359 | -0.3359 |  |
|  |  | 298.078 | 1.0202 |  | 0.3928 | 0.3928 | -0.3928 |  |
|  |  | 298.157 | 0.1-40.265 |  | 0.3576 | 0.3919 | -0.3576 |  |
|  |  | 303.935 | 60.074 |  | 0.3235 | 0.3235 | -0.3235 |  |
|  |  | 304.201 | 39.942 |  | 0.3345 | 0.3345 | -0.3345 |  |
|  |  | 304.225 | 49.724 |  | 0.3280 | 0.3280 | -0.3280 |  |
|  |  | 304.281 | 29.918 |  | 0.3495 | 0.3495 | -0.3495 |  |
|  |  | 304.344 | 10.634 |  | 0.3840 | 0.3840 | -0.3840 |  |
|  |  | 310.522 | 20.049 |  | 0.3736 | 0.3736 | -0.3736 |  |
|  |  | 310.573 | 59.616 |  | 0.3296 | 0.3296 | -0.3296 |  |
|  |  | 310.652 | 49.941 |  | 0.3322 | 0.3322 | -0.3322 |  |
|  |  | 310.805 | 40.003 |  | 0.3433 | 0.3433 | -0.3433 |  |
|  |  | 311.244 | 1.092 |  | 0.4179 | 0.4179 | -0.4179 |  |
|  |  | 317.167 | 9.765 |  | 0.4056 | 0.4056 | -0.4056 |  |
|  |  | 317.186 | 29.905 |  | 0.3677 | 0.3677 | -0.3677 |  |
|  |  | 317.225 | 59.899 |  | 0.3320 | 0.3320 | -0.3320 |  |
|  |  | 317.445 | 50.073 |  | 0.3384 | 0.3384 | -0.3384 |  |
|  |  | 324.195 | 60.082 |  | 0.3353 | 0.3353 | -0.3353 |  |
|  |  | 324.282 | 20.103 |  | 0.3882 | 0.3882 | -0.3882 |  |
|  |  | 325.017 | 1.1271 |  | 0.4403 | 0.4403 | -0.4403 |  |
|  |  | 331.281 | 30.008 |  | 0.3752 | 0.3752 | -0.3752 |  |
|  |  | 331.293 | 9.8624 |  | 0.4230 | 0.4230 | -0.4230 |  |
|  |  | 337.917 | 60.055 |  | 0.3338 | 0.3338 | -0.3338 |  |
|  |  | 338.387 | 40.023 |  | 0.3577 | 0.3577 | -0.3577 |  |
|  |  | 338.742 | 20.149 |  | 0.3993 | 0.3993 | -0.3993 |  |
|  |  | 339.672 | 1.074 |  | 0.4561 | 0.4561 | -0.4561 |  |
|  |  | 345.693 | 50.073 |  | 0.3433 | 0.3433 | -0.3433 |  |
|  |  | 346.215 | 30.275 |  | 0.3770 | 0.3770 | -0.3770 |  |
|  |  | 346.223 | 9.8731 |  | 0.4321 | 0.4321 | -0.4321 |  |
| [C_2_mim][BF_4_] | REF 11 | 293.15 | 0.1-30 | for  T = 393.15 K  and  p = 30 MPa  0.09 % | 0.0241 | 0.0564 | -0.0241 | Isothermal Compressibility from REF 11 |
|  |  | 303.15 | 0.1-30 |  | 0.0163 | 0.0360 | 0.0057 |  |
|  |  | 313.15 | 0.1-30 |  | 0.0149 | 0.0590 | -0.0147 |  |
|  |  | 323.15 | 0.1-30 |  | 0.0160 | 0.0646 | -0.0098 |  |
|  |  | 333.15 | 0.1-30 |  | 0.0148 | 0.0601 | -0.0142 |  |
|  |  | 343.15 | 0.1-30 |  | 0.0249 | 0.0689 | -0.0249 |  |
|  |  | 353.15 | 0.1-30 |  | 0.0190 | 0.0598 | -0.0190 |  |
|  |  | 393.15 | 0.1-30 |  | 0.0214 | 0.0887 | -0.0177 |  |
| [C_2_mim][BF_4_] | REF 12 | 293.15 | 0.1 | for  T = 312.2 K  and  p = 200 MPa  0.44 % | 0.0008 | 0.0008 | 0.0008 | Isothermal Compressibility from REF 11 |
|  |  | 313.15 | 0.1 |  | 0.0024 | 0.0024 | -0.0024 |  |
|  |  | 333.15 | 0.1 |  | 0.0024 | 0.0024 | 0.0024 |  |
|  |  | 353.15 | 0.1 |  | 0.0008 | 0.0008 | -0.0008 |  |
|  |  | 312.2 | 10-200 |  | 0.2064 | 0.4445 | -0.2016 |  |
|  |  | 332.6 | 10-200 |  | 0.2057 | 0.4201 | -0.2057 |  |
|  |  | 352.7 | 10-200 |  | 0.1821 | 0.3580 | -0.1778 |  |
|  |  | 372.8 | 10-200 |  | 0.1515 | 0.3482 | -0.1493 |  |
|  |  | 392.9 | 10-200 |  | 0.1336 | 0.2629 | -0.1287 |  |
|  |  | 413 | 10-200 |  | 0.1188 | 0.2601 | -0.1125 |  |
|  |  | 432.9 | 10-200 |  | 0.0710 | 0.1747 | -0.0645 |  |
|  |  | 452.6 | 10-200 |  | 0.0394 | 0.1392 | -0.0256 |  |
|  |  | 472.4 | 10-200 |  | 0.0387 | 0.0903 | 0.0141 |  |
| [C_2_mim][BF_4_] | REF 13 | 283.15 | 0.1-60 | for  T = 283.15 K  and  p = 60 MPa  0.14 % | 0.0409 | 0.1416 | -0.0408 | Isothermal Compressibility from REF 11 |
|  |  | 288.15 | 0.1-60 |  | 0.0401 | 0.1302 | -0.0401 |  |
|  |  | 293.15 | 0.1-60 |  | 0.0355 | 0.1165 | -0.0355 |  |
|  |  | 298.15 | 0.1-60 |  | 0.0330 | 0.1120 | -0.0327 |  |
|  |  | 303.15 | 0.1-60 |  | 0.0327 | 0.1076 | -0.0327 |  |
|  |  | 308.15 | 0.1-60 |  | 0.0287 | 0.0948 | -0.0286 |  |
|  |  | 313.15 | 0.1-60 |  | 0.0276 | 0.0929 | -0.0276 |  |
|  |  | 318.15 | 0.1-60 |  | 0.0272 | 0.0895 | -0.0269 |  |
|  |  | 323.15 | 0.1-60 |  | 0.0257 | 0.0808 | -0.0257 |  |
| [C_2_mim][BF_4_] | REF 14 | 284.727 | 40.082 | for  T = 285.119 K  and  p = 9.9935 MPa  1.54 % | 1.3926 | 1.3926 | 1.3926 | Density (p = 0.1 MPa) and Isothermal Compressibility from REF 11 |
|  |  | 284.743 | 29.331 |  | 1.3934 | 1.3934 | 1.3934 |  |
|  |  | 285.119 | 9.9935 |  | 1.5176 | 1.5176 | 1.5176 |  |
|  |  | 285.204 | 19.389 |  | 1.4486 | 1.4486 | 1.4486 |  |
|  |  | 285.804 | 1.3269 |  | 1.4697 | 1.4697 | 1.4697 |  |
|  |  | 290.95 | 50.018 |  | 1.3231 | 1.3231 | 1.3231 |  |
|  |  | 291.302 | 39.987 |  | 1.3282 | 1.3282 | 1.3282 |  |
|  |  | 291.577 | 20.094 |  | 1.4471 | 1.4471 | 1.4471 |  |
|  |  | 291.59 | 10.267 |  | 1.4066 | 1.4066 | 1.4066 |  |
|  |  | 292.001 | 30.197 |  | 1.3784 | 1.3784 | 1.3784 |  |
|  |  | 292.029 | 1.0554 |  | 1.4063 | 1.4063 | 1.4063 |  |
|  |  | 298.143 | 10.219 |  | 1.3420 | 1.3420 | 1.3420 |  |
|  |  | 298.145 | 19.834 |  | 1.3264 | 1.3264 | 1.3264 |  |
|  |  | 298.146 | 20.203-61.242 |  | 1.3117 | 1.3525 | 1.3117 |  |
|  |  | 298.147 | 39.935 |  | 1.3222 | 1.3222 | 1.3222 |  |
|  |  | 298.153 | 39.774-50.71 |  | 1.2881 | 1.3173 | 1.2881 |  |
|  |  | 298.162 | 30.299 |  | 1.3829 | 1.3829 | 1.3829 |  |
|  |  | 298.634 | 1.1397 |  | 1.3617 | 1.3617 | 1.3617 |  |
|  |  | 304.088 | 59.595 |  | 1.1951 | 1.1951 | 1.1951 |  |
|  |  | 304.388 | 39.527 |  | 1.3182 | 1.3182 | 1.3182 |  |
|  |  | 304.61 | 49.734 |  | 1.2594 | 1.2594 | 1.2594 |  |
|  |  | 304.62 | 29.983 |  | 1.2793 | 1.2793 | 1.2793 |  |
|  |  | 304.693 | 19.721 |  | 1.2729 | 1.2729 | 1.2729 |  |
|  |  | 304.695 | 10.037 |  | 1.3040 | 1.3040 | 1.3040 |  |
|  |  | 305.23 | 1.1941 |  | 1.3048 | 1.3048 | 1.3048 |  |
|  |  | 311.405 | 39.938 |  | 1.2185 | 1.2185 | 1.2185 |  |
|  |  | 311.493 | 30.054 |  | 1.2264 | 1.2264 | 1.2264 |  |
|  |  | 311.508 | 60.021 |  | 1.1970 | 1.1970 | 1.1970 |  |
|  |  | 311.554 | 19.542 |  | 1.2298 | 1.2298 | 1.2298 |  |
|  |  | 311.556 | 10.293 |  | 1.2519 | 1.2519 | 1.2519 |  |
|  |  | 311.72 | 50.382 |  | 1.2528 | 1.2528 | 1.2528 |  |
|  |  | 312.051 | 1.1115 |  | 1.2749 | 1.2749 | 1.2749 |  |
|  |  | 318.36 | 19.918 |  | 1.1987 | 1.1987 | 1.1987 |  |
|  |  | 318.367 | 50.094 |  | 1.1648 | 1.1648 | 1.1648 |  |
|  |  | 318.372 | 9.976 |  | 1.2263 | 1.2263 | 1.2263 |  |
|  |  | 318.404 | 39.958 |  | 1.1708 | 1.1708 | 1.1708 |  |
|  |  | 318.494 | 30.004 |  | 1.1947 | 1.1947 | 1.1947 |  |
|  |  | 318.573 | 60.242 |  | 1.1924 | 1.1924 | 1.1924 |  |
|  |  | 319.239 | 1.1556 |  | 1.2360 | 1.2360 | 1.2360 |  |
|  |  | 325.39 | 60.094 |  | 1.1130 | 1.1130 | 1.1130 |  |
|  |  | 325.502 | 50.05 |  | 1.1211 | 1.1211 | 1.1211 |  |
|  |  | 325.589 | 30.106-39.966 |  | 1.1489 | 1.1529 | 1.1489 |  |
|  |  | 325.651 | 9.9021 |  | 1.1927 | 1.1927 | 1.1927 |  |
|  |  | 325.659 | 18.72 |  | 1.1315 | 1.1315 | 1.1315 |  |
|  |  | 326.257 | 1.1665 |  | 1.2496 | 1.2496 | 1.2496 |  |
|  |  | 332.567 | 59.788 |  | 1.0727 | 1.0727 | 1.0727 |  |
|  |  | 332.792 | 39.915 |  | 1.1073 | 1.1073 | 1.1073 |  |
|  |  | 332.852 | 50.074 |  | 1.1016 | 1.1016 | 1.1016 |  |
|  |  | 332.865 | 29.859 |  | 1.1352 | 1.1352 | 1.1352 |  |
|  |  | 332.985 | 10.159 |  | 1.2125 | 1.2125 | 1.2125 |  |
|  |  | 333.066 | 19.96 |  | 1.1519 | 1.1519 | 1.1519 |  |
|  |  | 333.62 | 1.2529 |  | 1.1975 | 1.1975 | 1.1975 |  |
|  |  | 340.278 | 59.745 |  | 1.0473 | 1.0473 | 1.0473 |  |
|  |  | 340.348 | 9.9692 |  | 1.1640 | 1.1640 | 1.1640 |  |
|  |  | 340.365 | 19.698 |  | 1.1703 | 1.1703 | 1.1703 |  |
|  |  | 340.39 | 50.227 |  | 1.0682 | 1.0682 | 1.0682 |  |
|  |  | 340.415 | 39.835 |  | 1.0924 | 1.0924 | 1.0924 |  |
|  |  | 340.548 | 29.899 |  | 1.1149 | 1.1149 | 1.1149 |  |
|  |  | 341.04 | 1.3571 |  | 1.1954 | 1.1954 | 1.1954 |  |
|  |  | 347.769 | 59.854 |  | 1.0287 | 1.0287 | 1.0287 |  |
|  |  | 347.971 | 10.024 |  | 1.1654 | 1.1654 | 1.1654 |  |
|  |  | 348.021 | 29.83 |  | 1.1480 | 1.1480 | 1.1480 |  |
|  |  | 348.043 | 19.82 |  | 1.1346 | 1.1346 | 1.1346 |  |
|  |  | 348.153 | 49.79 |  | 1.0511 | 1.0511 | 1.0511 |  |
|  |  | 348.237 | 39.938 |  | 1.0837 | 1.0837 | 1.0837 |  |
|  |  | 355.847 | 20.035 |  | 1.1502 | 1.1502 | 1.1502 |  |
|  |  | 355.861 | 29.675 |  | 1.1109 | 1.1109 | 1.1109 |  |
|  |  | 356.015 | 40.147 |  | 1.1279 | 1.1279 | 1.1279 |  |
|  |  | 356.019 | 49.918 |  | 1.0572 | 1.0572 | 1.0572 |  |
|  |  | 356.401 | 59.86 |  | 1.0020 | 1.0020 | 1.0020 |  |
| [C_2_mim][C_2_SO_4_] | REF 15 | 293.15 | 0.1-60 | for  T = 293.15 K  and  p = 0.25 MPa  0.02 % | 0.0174 | 0.0206 | -0.0174 | Isothermal Compressibility from REF 15 |
|  |  | 303.15 | 0.1-60 |  | 0.0017 | 0.0060 | -0.0007 |  |
|  |  | 313.15 | 0.1-60 |  | 0.0098 | 0.0144 | 0.0098 |  |
|  |  | 323.15 | 0.1-60 |  | 0.0116 | 0.0152 | 0.0116 |  |
|  |  | 333.15 | 0.1-60 |  | 0.0117 | 0.0149 | 0.0117 |  |
|  |  | 343.15 | 0.1-60 |  | 0.0079 | 0.0123 | 0.0079 |  |
|  |  | 353.15 | 0.1-60 |  | 0.0044 | 0.0116 | 0.0033 |  |
|  |  | 363.15 | 0.1-60 |  | 0.0025 | 0.0077 | -0.0011 |  |
|  |  | 373.15 | 0.1-60 |  | 0.0061 | 0.0110 | -0.0060 |  |
|  |  | 393.15 | 0.1-60 |  | 0.0126 | 0.0181 | -0.0126 |  |
|  |  | 413.15 | 0.1-60 |  | 0.0091 | 0.0161 | -0.0091 |  |
|  |  | 433.15 | 0.1-60 |  | 0.0122 | 0.0199 | 0.0122 |  |
| [C_2_mim][C_2_SO_4_] | REF 18 | 293.15 | 0.1-35 | for  T = 393.15 K  and  p = 35 MPa  0.20 % | 0.0990 | 0.1282 | -0.0974 | Isothermal Compressibility from REF 18 |
|  |  | 298.15 | 0.1-35 |  | 0.0992 | 0.1282 | -0.0992 |  |
|  |  | 303.15 | 0.1-35 |  | 0.1085 | 0.1413 | -0.1085 |  |
|  |  | 308.15 | 0.1-35 |  | 0.0864 | 0.1309 | -0.0864 |  |
|  |  | 333.15 | 0.1-35 |  | 0.0469 | 0.0828 | -0.0469 |  |
|  |  | 353.15 | 0.1-35 |  | 0.0378 | 0.0683 | -0.0378 |  |
|  |  | 373.15 | 0.1-35 |  | 0.0404 | 0.0616 | 0.0404 |  |
|  |  | 393.15 | 0.1-35 |  | 0.1255 | 0.2042 | 0.1255 |  |
| [C_2_mim][C_2_SO_4_] | REF 34 | 283.15 | 0.1-35 | for  T = 340.65 K  and  p = 35 MPa  0.05 % | 0.0149 | 0.0214 | -0.0149 | Isothermal Compressibility from REF 15 |
|  |  | 285.65 | 0.1-35 |  | 0.0124 | 0.0202 | -0.0124 |  |
|  |  | 288.15 | 0.1-35 |  | 0.0141 | 0.0236 | -0.0141 |  |
|  |  | 290.65 | 0.1-35 |  | 0.0106 | 0.0197 | -0.0106 |  |
|  |  | 293.15 | 0.1-35 |  | 0.0068 | 0.0156 | -0.0046 |  |
|  |  | 295.65 | 0.1-35 |  | 0.0067 | 0.0154 | -0.0039 |  |
|  |  | 298.15 | 0.1-35 |  | 0.0062 | 0.0118 | -0.0003 |  |
|  |  | 300.65 | 0.1-35 |  | 0.0057 | 0.0119 | 0.0005 |  |
|  |  | 303.15 | 0.1-35 |  | 0.0078 | 0.0185 | -0.0067 |  |
|  |  | 305.65 | 0.1-35 |  | 0.0079 | 0.0176 | -0.0031 |  |
|  |  | 308.15 | 0.1-35 |  | 0.0103 | 0.0230 | -0.0097 |  |
|  |  | 310.65 | 0.1-35 |  | 0.0112 | 0.0250 | -0.0103 |  |
|  |  | 313.15 | 0.1-35 |  | 0.0107 | 0.0244 | -0.0095 |  |
|  |  | 315.65 | 0.1-35 |  | 0.0112 | 0.0253 | -0.0102 |  |
|  |  | 318.15 | 0.1-35 |  | 0.0211 | 0.0366 | -0.0211 |  |
|  |  | 320.65 | 0.1-35 |  | 0.0215 | 0.0379 | -0.0215 |  |
|  |  | 323.15 | 0.1-35 |  | 0.0199 | 0.0367 | -0.0199 |  |
|  |  | 325.65 | 0.1-35 |  | 0.0213 | 0.0394 | -0.0213 |  |
|  |  | 328.15 | 0.1-35 |  | 0.0248 | 0.0435 | -0.0248 |  |
|  |  | 330.65 | 0.1-35 |  | 0.0227 | 0.0409 | -0.0227 |  |
|  |  | 333.15 | 0.1-35 |  | 0.0207 | 0.0398 | -0.0207 |  |
|  |  | 335.65 | 0.1-35 |  | 0.0249 | 0.0418 | -0.0249 |  |
|  |  | 338.15 | 0.1-35 |  | 0.0140 | 0.0314 | -0.0132 |  |
|  |  | 340.65 | 0.1-35 |  | 0.0278 | 0.0477 | -0.0278 |  |
|  |  | 343.15 | 0.1-35 |  | 0.0115 | 0.0279 | -0.0108 |  |
|  |  | 345.65 | 0.1-35 |  | 0.0119 | 0.0266 | -0.0032 |  |
|  |  | 348.15 | 0.1-35 |  | 0.0143 | 0.0335 | -0.0143 |  |
|  |  | 350.65 | 0.1-35 |  | 0.0156 | 0.0344 | -0.0144 |  |
|  |  | 353.15 | 0.1-35 |  | 0.0150 | 0.0326 | -0.0123 |  |
| [C_2_mim][C_2_SO_4_] | REF 35 | 283.15 | 0.1-35 | for  T = 313.15 K  and  p = 35 MPa  0.03 % | 0.0030 | 0.0068 | -0.0030 | Isothermal Compressibility from REF 15 |
|  |  | 293.15 | 0.1-35 |  | 0.0074 | 0.0147 | -0.0074 |  |
|  |  | 298.15 | 0.1-35 |  | 0.0089 | 0.0178 | -0.0089 |  |
|  |  | 303.15 | 0.1-35 |  | 0.0101 | 0.0170 | -0.0051 |  |
|  |  | 313.15 | 0.1-35 |  | 0.0192 | 0.0335 | -0.0192 |  |
|  |  | 323.15 | 0.1-35 |  | 0.0167 | 0.0322 | -0.0167 |  |
|  |  | 333.15 | 0.1-35 |  | 0.0127 | 0.0276 | -0.0121 |  |
| [C_2_mim][C_2_SO_4_] | REF 36 | 283.15 | 0.1-35 | for  T = 338.15 K  and  p = 35 MPa  0.04 % | 0.0089 | 0.0186 | -0.0089 | Isothermal Compressibility from REF 15 |
|  |  | 288.15 | 0.1-35 |  | 0.0087 | 0.0177 | -0.0087 |  |
|  |  | 293.15 | 0.1-35 |  | 0.0067 | 0.0162 | -0.0053 |  |
|  |  | 298.15 | 0.1-35 |  | 0.0081 | 0.0192 | -0.0081 |  |
|  |  | 303.15 | 0.1-35 |  | 0.0124 | 0.0276 | -0.0124 |  |
|  |  | 308.15 | 0.1-35 |  | 0.0102 | 0.0237 | -0.0092 |  |
|  |  | 313.15 | 0.1-35 |  | 0.0129 | 0.0276 | -0.0126 |  |
|  |  | 318.15 | 0.1-35 |  | 0.0210 | 0.0377 | -0.0210 |  |
|  |  | 323.15 | 0.1-35 |  | 0.0190 | 0.0370 | -0.0190 |  |
|  |  | 328.15 | 0.1-35 |  | 0.0167 | 0.0359 | -0.0167 |  |
|  |  | 333.15 | 0.1-35 |  | 0.0150 | 0.0314 | -0.0103 |  |
|  |  | 338.15 | 0.1-35 |  | 0.0179 | 0.0379 | -0.0179 |  |
|  |  | 343.15 | 0.1-35 |  | 0.0160 | 0.0342 | -0.0160 |  |
| [C_2_mim][C_2_SO_4_] | REF 37 | 278.15 | 0.1-120 | for  T = 373.15 K  and  p = 120 MPa  0.19 % | 0.0311 | 0.0626 | 0.0311 | Isothermal Compressibility from REF 15 |
|  |  | 278.15 | 0.1-120 |  | 0.0451 | 0.0580 | -0.0451 |  |
|  |  | 298.15 | 0.1-120 |  | 0.0403 | 0.0488 | 0.0403 |  |
|  |  | 298.15 | 0.1-120 |  | 0.0154 | 0.0417 | -0.0136 |  |
|  |  | 313.15 | 0.1-120 |  | 0.0182 | 0.0357 | 0.0013 |  |
|  |  | 313.15 | 0.1-120 |  | 0.0379 | 0.0908 | -0.0379 |  |
|  |  | 333.15 | 0.1-120 |  | 0.0357 | 0.1082 | -0.0284 |  |
|  |  | 333.15 | 0.1-120 |  | 0.0518 | 0.1400 | -0.0518 |  |
|  |  | 348.15 | 0.1-120 |  | 0.0553 | 0.1522 | -0.0547 |  |
|  |  | 348.15 | 0.1-120 |  | 0.0729 | 0.1763 | -0.0729 |  |
|  |  | 373.15 | 0.1-120 |  | 0.0609 | 0.1695 | -0.0573 |  |
|  |  | 373.15 | 0.1-120 |  | 0.0723 | 0.1857 | -0.0710 |  |
|  |  | 398.15 | 0.1-120 |  | 0.0537 | 0.1282 | -0.0066 |  |
|  |  | 398.15 | 0.1-120 |  | 0.0536 | 0.1445 | -0.0159 |  |
| [C_2_mim][C_2_SO_4_] | REF 40 | 278.15 | 0.1-120 | for  T = 373.15 K  and  p = 120 MPa  0.18% | 0.0149 | 0.0243 | -0.0095 | Isothermal Compressibility from REF 15 |
|  |  | 298.15 | 0.1-120 |  | 0.0168 | 0.0282 | 0.0108 |  |
|  |  | 313.15 | 0.1-120 |  | 0.0250 | 0.0706 | -0.0204 |  |
|  |  | 333.15 | 0.1-120 |  | 0.0435 | 0.1275 | -0.0414 |  |
|  |  | 348.15 | 0.1-120 |  | 0.0662 | 0.1680 | -0.0662 |  |
|  |  | 373.15 | 0.1-120 |  | 0.0696 | 0.1820 | -0.0675 |  |
|  |  | 398.15 | 0.1-120 |  | 0.0635 | 0.1421 | -0.0030 |  |
| [C_2_mim][C_6_SO_4_] | REF 74 | 278.15 | 0.1-120 | for  T = 348.15 K  and  p = 120 MPa  0.10 % | 0.0382 | 0.0558 | 0.0264 | Isothermal Compressibility calculated from Tait equation from REF 74 |
|  |  | 298.15 | 0.1-120 |  | 0.0315 | 0.0663 | 0.0168 |  |
|  |  | 313.15 | 0.1-120 |  | 0.0216 | 0.0502 | 0.0105 |  |
|  |  | 333.15 | 0.1-120 |  | 0.0212 | 0.0865 | -0.0021 |  |
|  |  | 348.15 | 0.1-120 |  | 0.0235 | 0.1013 | -0.0213 |  |
|  |  | 373.15 | 0.1-120 |  | 0.0310 | 0.0858 | -0.0238 |  |
|  |  | 398.15 | 0.1-120 |  | 0.0431 | 0.0908 | -0.0431 |  |
| [C_2_mim][C_1_SO_4_] | REF 18 | 293.15 | 0.1-35 | for  T = 293.15 K  and  p = 35 MPa  1.29 % | 0.5108 | 1.2764 | 0.5096 | Isothermal Compressibility from REF 18 |
|  |  | 298.15 | 0.1-35 |  | 0.4489 | 1.1299 | 0.4484 |  |
|  |  | 303.15 | 0.1-35 |  | 0.4579 | 1.0675 | 0.4579 |  |
|  |  | 308.15 | 0.1-35 |  | 0.2809 | 0.7540 | 0.2694 |  |
|  |  | 333.15 | 0.1-35 |  | 0.1655 | 0.4584 | 0.1571 |  |
|  |  | 353.15 | 0.1-35 |  | 0.0741 | 0.2180 | 0.0637 |  |
|  |  | 373.15 | 0.1-35 |  | 0.0451 | 0.1170 | 0.0451 |  |
|  |  | 393.15 | 0.1-35 |  | 0.0107 | 0.0307 | 0.0043 |  |
| [C_2_mim][DEP] | REF 82 | 293.15 | 0.1 | for  T = 411.1 K  and  p = 200 MPa  0.23 % | 0.0101 | 0.0101 | -0.0101 | Isothermal Compressibility calculated from Tait equation from REF 82 |
|  |  | 303.15 | 0.1 |  | 0.0071 | 0.0071 | 0.0071 |  |
|  |  | 313.15 | 0.1 |  | 0.0110 | 0.0110 | 0.0110 |  |
|  |  | 323.15 | 0.1 |  | 0.0015 | 0.0015 | 0.0015 |  |
|  |  | 333.15 | 0.1 |  | 0.0041 | 0.0041 | -0.0041 |  |
|  |  | 343.15 | 0.1 |  | 0.0054 | 0.0054 | -0.0054 |  |
|  |  | 353.15 | 0.1 |  | 0.0025 | 0.0025 | -0.0025 |  |
|  |  | 363.15 | 0.1 |  | 0.0045 | 0.0045 | -0.0045 |  |
|  |  | 373.15 | 0.1 |  | 0.0070 | 0.0070 | 0.0070 |  |
|  |  | 311.4 | 10-200 |  | 0.0379 | 0.1164 | -0.0360 |  |
|  |  | 331.3 | 10-200 |  | 0.0565 | 0.1422 | -0.0552 |  |
|  |  | 351.3 | 10-200 |  | 0.0662 | 0.1630 | -0.0652 |  |
|  |  | 371.3 | 10-200 |  | 0.0827 | 0.1908 | -0.0826 |  |
|  |  | 391.2 | 10-200 |  | 0.0996 | 0.2046 | -0.0996 |  |
|  |  | 411.1 | 10-200 |  | 0.1167 | 0.2258 | -0.1167 |  |
| [C_2_mim][Lactate] | REF 8 | 293.15 | 0.1 | for  T = 432.2 K  and  p = 20 MPa  0.20 % | 0.0066 | 0.0066 | -0.0066 | Isothermal Compressibility from REF 8 |
|  |  | 298.15 | 0.1 |  | 0.0012 | 0.0012 | -0.0012 |  |
|  |  | 303.15 | 0.1 |  | 0.0064 | 0.0064 | 0.0064 |  |
|  |  | 308.15 | 0.1 |  | 0.0075 | 0.0075 | 0.0075 |  |
|  |  | 313.15 | 0.1 |  | 0.0020 | 0.0020 | 0.0020 |  |
|  |  | 318.15 | 0.1 |  | 0.0012 | 0.0012 | -0.0012 |  |
|  |  | 323.15 | 0.1 |  | 0.0022 | 0.0022 | -0.0022 |  |
|  |  | 328.15 | 0.1 |  | 0.0009 | 0.0009 | -0.0009 |  |
|  |  | 333.15 | 0.1 |  | 0.0063 | 0.0063 | -0.0063 |  |
|  |  | 338.15 | 0.1 |  | 0.0004 | 0.0004 | -0.0004 |  |
|  |  | 343.15 | 0.1 |  | 0.0013 | 0.0013 | -0.0013 |  |
|  |  | 348.15 | 0.1 |  | 0.0002 | 0.0002 | 0.0002 |  |
|  |  | 353.15 | 0.1 |  | 0.0040 | 0.0040 | 0.0040 |  |
|  |  | 312.6 | 10-200 |  | 0.0692 | 0.1664 | -0.0669 |  |
|  |  | 332.1 | 10-200 |  | 0.0551 | 0.1542 | -0.0410 |  |
|  |  | 352.1 | 10-200 |  | 0.0370 | 0.1289 | -0.0203 |  |
|  |  | 372.2 | 10-200 |  | 0.0526 | 0.1564 | -0.0396 |  |
|  |  | 392.4 | 10-200 |  | 0.0616 | 0.1518 | -0.0246 |  |
|  |  | 412.4 | 10-200 |  | 0.0629 | 0.1186 | 0.0169 |  |
|  |  | 432.2 | 10-200 |  | 0.0889 | 0.1981 | 0.0532 |  |
| [C_2_mim][MP] | REF 82 | 293.15 | 0.1 | for  T = 311.2 K  and  p = 200 MPa  0.38 % | 0.0008 | 0.0008 | -0.0008 | Isothermal Compressibility calculated from Tait equation from REF 82 |
|  |  | 303.15 | 0.1 |  | 0.0033 | 0.0033 | 0.0033 |  |
|  |  | 313.15 | 0.1 |  | 0.0042 | 0.0042 | -0.0042 |  |
|  |  | 323.15 | 0.1 |  | 0.0020 | 0.0020 | 0.0020 |  |
|  |  | 333.15 | 0.1 |  | 0.0035 | 0.0035 | -0.0035 |  |
|  |  | 343.15 | 0.1 |  | 0.0049 | 0.0049 | 0.0049 |  |
|  |  | 353.15 | 0.1 |  | 0.0015 | 0.0015 | 0.0015 |  |
|  |  | 363.15 | 0.1 |  | 0.0053 | 0.0053 | -0.0053 |  |
|  |  | 373.15 | 0.1 |  | 0.0021 | 0.0021 | 0.0021 |  |
|  |  | 311.2 | 10-200 |  | 0.1408 | 0.3844 | -0.1114 |  |
|  |  | 330.9 | 10-200 |  | 0.0983 | 0.2568 | -0.0438 |  |
|  |  | 351.3 | 10-200 |  | 0.0632 | 0.1511 | 0.0030 |  |
|  |  | 371.3 | 10-200 |  | 0.0641 | 0.1445 | 0.0557 |  |
|  |  | 391.2 | 10-200 |  | 0.0928 | 0.1192 | 0.0928 |  |
|  |  | 411.1 | 10-200 |  | 0.1316 | 0.1647 | 0.1316 |  |
| [C_2_mim][NTf_2_] | REF 11 | 293.15 | 0.1-30 | for  T = 393.15 K  and  p = 30 MPa  0.16 % | 0.0178 | 0.0832 | -0.0158 | Isothermal Compressibility from REF 11 |
|  |  | 303.15 | 0.1-30 |  | 0.0247 | 0.0913 | -0.0247 |  |
|  |  | 313.15 | 0.1-30 |  | 0.0239 | 0.0900 | -0.0239 |  |
|  |  | 323.15 | 0.1-30 |  | 0.0263 | 0.1054 | -0.0263 |  |
|  |  | 333.15 | 0.1-30 |  | 0.0244 | 0.1114 | -0.0243 |  |
|  |  | 343.15 | 0.1-30 |  | 0.0222 | 0.1011 | -0.0172 |  |
|  |  | 353.15 | 0.1-30 |  | 0.0245 | 0.1146 | -0.0214 |  |
|  |  | 393.15 | 0.1-30 |  | 0.0390 | 0.1621 | -0.0390 |  |
| [C_2_mim][NTf_2_] | REF 26 | 293.49 | 0.1 | for  T = 322.3 K  and  p = 0.1 MPa  0.02 % | 0.0005 | 0.0005 | -0.0005 | Isothermal Compressibility from REF 26 |
|  |  | 302.68 | 0.1 |  | 0.0080 | 0.0080 | -0.0080 |  |
|  |  | 312.64 | 0.1 |  | 0.0052 | 0.0052 | -0.0052 |  |
|  |  | 322.3 | 0.1 |  | 0.0196 | 0.0196 | 0.0196 |  |
|  |  | 332.58 | 0.1 |  | 0.0049 | 0.0049 | 0.0049 |  |
|  |  | 342.49 | 0.1 |  | 0.0102 | 0.0102 | -0.0102 |  |
|  |  | 352.32 | 0.1 |  | 0.0091 | 0.0091 | 0.0091 |  |
|  |  | 373.32 | 0.1 |  | 0.0091 | 0.0091 | -0.0091 |  |
|  |  | 391.29 | 0.1 |  | 0.0081 | 0.0081 | -0.0081 |  |
|  |  | 414.92 | 0.1 |  | 0.0073 | 0.0073 | 0.0073 |  |
|  |  | 293.5 | 1-40 |  | 0.0050 | 0.0124 | 0.0027 |  |
|  |  | 322.32 | 1-40 |  | 0.0055 | 0.0082 | -0.0051 |  |
|  |  | 352.34 | 1-40 |  | 0.0119 | 0.0180 | -0.0119 |  |
|  |  | 373.31 | 1-40 |  | 0.0109 | 0.0163 | -0.0109 |  |
|  |  | 391.25 | 1-40 |  | 0.0044 | 0.0089 | -0.0037 |  |
|  |  | 414.93 | 1-40 |  | 0.0083 | 0.0174 | 0.0083 |  |
| [C_2_mim][NTf_2_] | REF 52 | 283.19 | 1.75-89.994 | for  T = 283.19 K  and  p = 1.75 MPa  0.31 % | 0.1800 | 0.3114 | 0.1800 | Density (p = 0.1 MPa) and Isothermal Compressibility from REF 11 |
|  |  | 283.2 | 10.046-40.033 |  | 0.2342 | 0.2834 | 0.2342 |  |
|  |  | 283.21 | 20.03-100.034 |  | 0.1598 | 0.2536 | 0.1598 |  |
|  |  | 293.14 | 99.755 |  | 0.0394 | 0.0394 | -0.0394 |  |
|  |  | 293.15 | 10.202-89.934 |  | 0.0879 | 0.1454 | 0.0575 |  |
|  |  | 293.16 | 20.787-69.993 |  | 0.0479 | 0.1193 | 0.0479 |  |
|  |  | 293.17 | 1.027-79.912 |  | 0.0951 | 0.1628 | 0.0879 |  |
|  |  | 293.19 | 39.872 |  | 0.0685 | 0.0685 | 0.0685 |  |
|  |  | 298.12 | 19.701-29.779 |  | 0.0795 | 0.0929 | 0.0795 |  |
|  |  | 298.13 | 39.926 |  | 0.0384 | 0.0384 | 0.0384 |  |
|  |  | 298.14 | 1.156 |  | 0.1373 | 0.1373 | 0.1373 |  |
|  |  | 298.15 | 49.997 |  | 0.0119 | 0.0119 | 0.0119 |  |
|  |  | 298.17 | 5.194 |  | 0.1258 | 0.1258 | 0.1258 |  |
|  |  | 298.18 | 90.001-99.95 |  | 0.0590 | 0.0616 | -0.0590 |  |
|  |  | 298.19 | 10.066-79.938 |  | 0.0638 | 0.1126 | 0.0113 |  |
|  |  | 298.2 | 59.613 |  | 0.0124 | 0.0124 | -0.0124 |  |
|  |  | 313.15 | 10.156-69.993 |  | 0.0528 | 0.0980 | -0.0459 |  |
|  |  | 313.16 | 1.963-89.994 |  | 0.0683 | 0.1245 | -0.0512 |  |
|  |  | 313.17 | 5.147-100.087 |  | 0.0793 | 0.1313 | -0.0520 |  |
|  |  | 333.13 | 80.064-89.994 |  | 0.2016 | 0.2047 | -0.2016 |  |
|  |  | 333.14 | 69.997-99.911 |  | 0.1968 | 0.2051 | -0.1968 |  |
|  |  | 333.15 | 60.111 |  | 0.1751 | 0.1751 | -0.1751 |  |
|  |  | 333.16 | 49.997 |  | 0.1581 | 0.1581 | -0.1581 |  |
|  |  | 333.17 | 2.359-40.159 |  | 0.1002 | 0.1398 | -0.1002 |  |
|  |  | 333.18 | 30.001 |  | 0.1206 | 0.1206 | -0.1206 |  |
|  |  | 333.19 | 9.791-18.829 |  | 0.0946 | 0.1015 | -0.0946 |  |
|  |  | 353.18 | 2.168-90.001 |  | 0.2030 | 0.2494 | -0.2030 |  |
|  |  | 353.19 | 9.673-99.944 |  | 0.1941 | 0.2454 | -0.1941 |  |
|  |  | 373.12 | 0.724 |  | 0.1333 | 0.1333 | -0.1333 |  |
|  |  | 373.14 | 4.935-99.92 |  | 0.2064 | 0.2264 | -0.2064 |  |
|  |  | 373.16 | 30.001-49.997 |  | 0.1889 | 0.2000 | -0.1889 |  |
|  |  | 373.17 | 10.147 |  | 0.1398 | 0.1398 | -0.1398 |  |
|  |  | 373.18 | 19.879 |  | 0.1549 | 0.1549 | -0.1549 |  |
| [C_2_mim][OAc] | REF 7 | 293.17 | 0.1-25 | for  T = 313.17 K  and  p = 25 MPa  0.46 % | 0.1187 | 0.3983 | -0.1107 | Isothermal Compressibility from REF 7 |
|  |  | 303.19 | 0.1-25 |  | 0.1315 | 0.4389 | -0.1305 |  |
|  |  | 313.17 | 0.1-25 |  | 0.1387 | 0.4583 | -0.1387 |  |
|  |  | 323.18 | 0.1-25 |  | 0.1315 | 0.4508 | -0.1248 |  |
|  |  | 333.15 | 0.1-25 |  | 0.1326 | 0.4575 | -0.1263 |  |
|  |  | 343.14 | 0.1-25 |  | 0.1308 | 0.4525 | -0.1284 |  |
|  |  | 353.16 | 0.1-25 |  | 0.1246 | 0.4241 | -0.1224 |  |
| [C_2_mim][OTf] | REF 45 | 293.15 | 0.1-35 | for  T = 353.15 K  and  p = 25 MPa  0.03 % | 0.0064 | 0.0132 | -0.0013 | Isothermal Compressibility from REF 45 |
|  |  | 303.15 | 0.1-35 |  | 0.0046 | 0.0077 | -0.0007 |  |
|  |  | 313.15 | 0.1-35 |  | 0.0074 | 0.0152 | -0.0070 |  |
|  |  | 323.15 | 0.1-35 |  | 0.0043 | 0.0087 | -0.0041 |  |
|  |  | 333.15 | 0.1-35 |  | 0.0082 | 0.0163 | 0.0082 |  |
|  |  | 353.15 | 0.1-35 |  | 0.0086 | 0.0297 | -0.0084 |  |
|  |  | 393.15 | 0.1-35 |  | 0.0095 | 0.0186 | 0.0082 |  |
| [C_2_mim][OTf] | REF 14 | 264.143 | 39.698 | for  T = 346.763 K  and  p = 20.122 MPa  1.04 % | 0.4814 | 0.4814 | -0.4814 | Density (p = 0.1 MPa) and Isothermal Compressibility from REF 45 |
|  |  | 264.213 | 29.714 |  | 0.4762 | 0.4762 | -0.4762 |  |
|  |  | 264.312 | 10.134 |  | 0.5227 | 0.5227 | -0.5227 |  |
|  |  | 265.067 | 1.2652 |  | 0.5304 | 0.5304 | -0.5304 |  |
|  |  | 271.029 | 50.162-50.199 |  | 0.5204 | 0.5213 | -0.5204 |  |
|  |  | 271.038 | 40.035-60.071 |  | 0.5132 | 0.5137 | -0.5132 |  |
|  |  | 271.039 | 40.309 |  | 0.5028 | 0.5028 | -0.5028 |  |
|  |  | 271.041 | 19.702-30.156 |  | 0.5563 | 0.5714 | -0.5563 |  |
|  |  | 271.042 | 11.097 |  | 0.5866 | 0.5866 | -0.5866 |  |
|  |  | 271.043 | 9.6773 |  | 0.5720 | 0.5720 | -0.5720 |  |
|  |  | 271.052 | 20.286 |  | 0.5492 | 0.5492 | -0.5492 |  |
|  |  | 271.401 | 1.1905 |  | 0.5571 | 0.5571 | -0.5571 |  |
|  |  | 272.278 | 2.6559 |  | 0.5528 | 0.5528 | -0.5528 |  |
|  |  | 277.453 | 49.84 |  | 0.5384 | 0.5384 | -0.5384 |  |
|  |  | 277.678 | 39.776 |  | 0.5846 | 0.5846 | -0.5846 |  |
|  |  | 277.795 | 30.125 |  | 0.5837 | 0.5837 | -0.5837 |  |
|  |  | 277.838 | 10.946 |  | 0.5635 | 0.5635 | -0.5635 |  |
|  |  | 277.844 | 19.967 |  | 0.5857 | 0.5857 | -0.5857 |  |
|  |  | 284.576 | 20.183 |  | 0.6065 | 0.6065 | -0.6065 |  |
|  |  | 284.65 | 40.227 |  | 0.6093 | 0.6093 | -0.6093 |  |
|  |  | 284.686 | 60.421 |  | 0.5792 | 0.5792 | -0.5792 |  |
|  |  | 284.725 | 50.105 |  | 0.6188 | 0.6188 | -0.6188 |  |
|  |  | 284.934 | 30.204 |  | 0.6148 | 0.6148 | -0.6148 |  |
|  |  | 285.095 | 0.7281 |  | 0.7268 | 0.7268 | -0.7268 |  |
|  |  | 291.424 | 29.93 |  | 0.6303 | 0.6303 | -0.6303 |  |
|  |  | 291.485 | 49.838 |  | 0.6477 | 0.6477 | -0.6477 |  |
|  |  | 291.916 | 10.046 |  | 0.7543 | 0.7543 | -0.7543 |  |
|  |  | 291.917 | 41.361 |  | 0.6692 | 0.6692 | -0.6692 |  |
|  |  | 291.94 | 40.088 |  | 0.6473 | 0.6473 | -0.6473 |  |
|  |  | 292.62 | 61.503 |  | 0.6566 | 0.6566 | -0.6566 |  |
|  |  | 298.187 | 39.944 |  | 0.6337 | 0.6337 | -0.6337 |  |
|  |  | 298.19 | 0.1242 |  | 0.8216 | 0.8216 | -0.8216 |  |
|  |  | 299.138 | 60.111 |  | 0.7028 | 0.7028 | -0.7028 |  |
|  |  | 299.193 | 50.157 |  | 0.6820 | 0.6820 | -0.6820 |  |
|  |  | 299.543 | 20.406 |  | 0.7815 | 0.7815 | -0.7815 |  |
|  |  | 300.18 | 1.6875 |  | 0.8283 | 0.8283 | -0.8283 |  |
|  |  | 305.754 | 49.851 |  | 0.6837 | 0.6837 | -0.6837 |  |
|  |  | 306.578 | 60.078 |  | 0.7246 | 0.7246 | -0.7246 |  |
|  |  | 306.751 | 10.122 |  | 0.8573 | 0.8573 | -0.8573 |  |
|  |  | 306.759 | 29.599 |  | 0.8279 | 0.8279 | -0.8279 |  |
|  |  | 313.345 | 59.888 |  | 0.7228 | 0.7228 | -0.7228 |  |
|  |  | 314.042 | 19.458 |  | 0.8849 | 0.8849 | -0.8849 |  |
|  |  | 314.641 | 40.402 |  | 0.8405 | 0.8405 | -0.8405 |  |
|  |  | 315.407 | 1.3775 |  | 0.9201 | 0.9201 | -0.9201 |  |
|  |  | 322.27 | 50.268 |  | 0.8713 | 0.8713 | -0.8713 |  |
|  |  | 322.294 | 30.09 |  | 0.9075 | 0.9075 | -0.9075 |  |
|  |  | 322.297 | 9.7991 |  | 0.9463 | 0.9463 | -0.9463 |  |
|  |  | 329.979 | 59.857 |  | 0.9117 | 0.9117 | -0.9117 |  |
|  |  | 330.678 | 40.501 |  | 0.9405 | 0.9405 | -0.9405 |  |
|  |  | 331.492 | 1.2566 |  | 0.9999 | 0.9999 | -0.9999 |  |
|  |  | 332.559 | 22.229 |  | 0.9831 | 0.9831 | -0.9831 |  |
|  |  | 338.105 | 9.0673 |  | 1.0161 | 1.0161 | -1.0161 |  |
|  |  | 338.466 | 50.063 |  | 0.9688 | 0.9688 | -0.9688 |  |
|  |  | 339.277 | 30.155 |  | 1.0109 | 1.0109 | -1.0109 |  |
|  |  | 346.743 | 59.641 |  | 1.0172 | 1.0172 | -1.0172 |  |
|  |  | 347.763 | 1.04 |  | 1.0492 | 1.0492 | -1.0492 |  |
|  |  | 347.765 | 40.116 |  | 1.0418 | 1.0418 | -1.0418 |  |
| [C_2_mim][PF_6_] | REF 12 | 312.8 | 10-200 | for  T = 312.8 K  and  p = 200 MPa  5.8 % | 3.4034 | 5.4974 | 3.4034 | Density - calculated values from REF 12; Isothermal Compressibility calculated using the GCM method proposed in REF 53 |
|  |  | 332.7 | 10-200 |  | 1.4807 | 3.2247 | 1.0275 |  |
|  |  | 352.7 | 10-200 |  | 2.0632 | 4.4244 | 1.7893 |  |
|  |  | 353.15 | 0.1 |  | 3.0302 | 3.0302 | 3.0302 |  |
|  |  | 372.8 | 10-200 |  | 3.1193 | 4.4209 | 3.1193 |  |
|  |  | 373.15 | 0.1 |  | 1.4217 | 1.4217 | 1.4217 |  |
|  |  | 392.9 | 10-200 |  | 1.9237 | 3.1631 | 1.9237 |  |
|  |  | 412.9 | 10-200 |  | 1.0838 | 2.0763 | 1.0530 |  |
|  |  | 432.6 | 10-200 |  | 0.5986 | 1.0964 | 0.5208 |  |
|  |  | 452.5 | 10-200 |  | 0.1306 | 0.3209 | 0.0129 |  |
|  |  | 472.4 | 10-200 |  | 1.7158 | 3.9391 | -1.5752 |  |
| [C_2_mim][SCN] | REF 6 | 298.15 | 0.1-10 | for  T = 328.15 K  and  p = 10 MPa  0.04 % | 0.0023 | 0.0051 | 0.0023 | Isothermal Compressibility from REF 6 |
|  |  | 308.15 | 0.1-10 |  | 0.0091 | 0.0108 | 0.0091 |  |
|  |  | 318.15 | 0.1-10 |  | 0.0313 | 0.0336 | -0.0313 |  |
|  |  | 328.15 | 0.1-10 |  | 0.0328 | 0.0357 | 0.0328 |  |
|  |  | 338.15 | 0.1-10 |  | 0.0127 | 0.0151 | -0.0127 |  |
| [C_2_mim][Tos] | REF 17 | 318.15 | 0.1-60 | for  T = 398.15 K  and  p = 50 MPa  0.09 % | 0.0534 | 0.0821 | 0.0534 | Isothermal Compressibility from REF 17 |
|  |  | 328.15 | 0.1-60 |  | 0.0458 | 0.0686 | 0.0458 |  |
|  |  | 338.15 | 0.1-60 |  | 0.0468 | 0.0753 | 0.0465 |  |
|  |  | 348.15 | 0.1-60 |  | 0.0494 | 0.0769 | 0.0491 |  |
|  |  | 358.15 | 0.1-60 |  | 0.0506 | 0.0784 | 0.0506 |  |
|  |  | 368.15 | 0.1-60 |  | 0.0461 | 0.0735 | 0.0456 |  |
|  |  | 378.15 | 0.1-60 |  | 0.0464 | 0.0814 | 0.0455 |  |
|  |  | 388.15 | 0.1-60 |  | 0.0462 | 0.0751 | 0.0443 |  |
|  |  | 398.15 | 0.1-60 |  | 0.0590 | 0.0849 | 0.0590 |  |
|  |  | 408.15 | 0.1-60 |  | 0.0557 | 0.0791 | 0.0557 |  |
|  |  | 418.15 | 0.1-60 |  | 0.0408 | 0.0629 | 0.0408 |  |
|  |  | 428.15 | 0.1-60 |  | 0.0107 | 0.0260 | -0.0011 |  |
| [C_2_OHC_1_NH_2_][C_2_COO] | REF 73 | 298.15 | 0.1-25 | for  T = 338.15 K  and  p = 25 MPa  0.51 % | 0.0248 | 0.0920 | -0.0194 | Isothermal Compressibility from REF 73 |
|  |  | 303.15 | 0.1-25 |  | 0.0211 | 0.0394 | 0.0211 |  |
|  |  | 308.15 | 0.1-25 |  | 0.0614 | 0.1054 | 0.0614 |  |
|  |  | 313.15 | 0.1-25 |  | 0.0991 | 0.2029 | 0.0991 |  |
|  |  | 318.15 | 0.1-25 |  | 0.1327 | 0.2963 | 0.1327 |  |
|  |  | 323.15 | 0.1-25 |  | 0.1602 | 0.3814 | 0.1601 |  |
|  |  | 328.15 | 0.1-25 |  | 0.1782 | 0.4509 | 0.1782 |  |
|  |  | 333.15 | 0.1-25 |  | 0.1852 | 0.4959 | 0.1851 |  |
|  |  | 338.15 | 0.1-25 |  | 0.1779 | 0.5090 | 0.1779 |  |
|  |  | 343.15 | 0.1-25 |  | 0.1543 | 0.4787 | 0.1543 |  |
|  |  | 348.15 | 0.1-25 |  | 0.1138 | 0.3983 | 0.1138 |  |
|  |  | 353.15 | 0.1-25 |  | 0.0623 | 0.2670 | 0.0570 |  |
|  |  | 358.15 | 0.1-25 |  | 0.0357 | 0.0896 | -0.0128 |  |
| [C_2_OHC_1_NH_2_][C_3_COO] | REF 73 | 298.15 | 0.1-25 | for  T = 358.15 K  and  p = 25 MPa  0.03 % | 0.0099 | 0.0204 | -0.0099 | Isothermal Compressibility from REF 73 |
|  |  | 308.15 | 0.1-25 |  | 0.0106 | 0.0208 | -0.0105 |  |
|  |  | 318.15 | 0.1-25 |  | 0.0114 | 0.0219 | -0.0114 |  |
|  |  | 328.15 | 0.1-25 |  | 0.0122 | 0.0248 | -0.0121 |  |
|  |  | 338.15 | 0.1-25 |  | 0.0128 | 0.0274 | -0.0128 |  |
|  |  | 348.15 | 0.1-25 |  | 0.0132 | 0.0307 | -0.0132 |  |
|  |  | 358.15 | 0.1-25 |  | 0.0132 | 0.0346 | -0.0132 |  |
| [C_2_OHC_1_NH_2_][C_4_COO] | REF 73 | 298.15 | 0.1-25 | for  T = 358.15 K  and  p = 25 MPa  0.05 % | 0.0115 | 0.0216 | -0.0098 | Isothermal Compressibility from REF 73 |
|  |  | 308.15 | 0.1-25 |  | 0.0123 | 0.0231 | -0.0123 |  |
|  |  | 318.15 | 0.1-25 |  | 0.0147 | 0.0253 | -0.0146 |  |
|  |  | 328.15 | 0.1-25 |  | 0.0169 | 0.0283 | -0.0169 |  |
|  |  | 338.15 | 0.1-25 |  | 0.0195 | 0.0339 | -0.0195 |  |
|  |  | 348.15 | 0.1-25 |  | 0.0214 | 0.0389 | -0.0214 |  |
|  |  | 358.15 | 0.1-25 |  | 0.0231 | 0.0454 | -0.0231 |  |
| [p3mpy][NTf_2_] | REF 45 | 293.15 | 0.1-35 | for  T = 393.15 K  and  p = 35 MPa  0.06 % | 0.0065 | 0.0107 | -0.0065 | Isothermal Compressibility from REF 45 |
|  |  | 303.15 | 0.1-35 |  | 0.0068 | 0.0147 | -0.0059 |  |
|  |  | 313.15 | 0.1-35 |  | 0.0082 | 0.0199 | 0.0064 |  |
|  |  | 323.15 | 0.1-35 |  | 0.0155 | 0.0426 | 0.0155 |  |
|  |  | 333.15 | 0.1-35 |  | 0.0171 | 0.0498 | 0.0165 |  |
|  |  | 353.15 | 0.1-35 |  | 0.0181 | 0.0453 | 0.0105 |  |
|  |  | 393.15 | 0.1-35 |  | 0.0162 | 0.0556 | 0.0114 |  |
| [C_3_mim][NTf_2_] | REF 54 | 298.15 | 0.1-59.59 | for  T = 298.15 K  and  p = 59.59 MPa  0.77 % | 0.2352 | 0.7669 | 0.2287 | Isothermal Compressibility from REF 54 |
|  |  | 299.15 | 0.1-59.59 |  | 0.2185 | 0.6969 | 0.2181 |  |
|  |  | 300.15 | 0.1-59.59 |  | 0.2015 | 0.6319 | 0.2015 |  |
|  |  | 301.15 | 0.1-59.59 |  | 0.1857 | 0.5698 | 0.1857 |  |
|  |  | 302.15 | 0.1-59.59 |  | 0.1680 | 0.5075 | 0.1680 |  |
|  |  | 303.15 | 0.1-59.59 |  | 0.1479 | 0.4516 | 0.1479 |  |
|  |  | 308.15 | 0.1-59.59 |  | 0.0698 | 0.2218 | 0.0685 |  |
|  |  | 313.15 | 0.1-59.59 |  | 0.0190 | 0.0569 | 0.0182 |  |
|  |  | 318.15 | 0.1-59.59 |  | 0.0253 | 0.0951 | -0.0253 |  |
|  |  | 323.15 | 0.1-59.59 |  | 0.0416 | 0.1324 | -0.0413 |  |
|  |  | 325.15 | 0.1-59.59 |  | 0.0608 | 0.1901 | -0.0605 |  |
|  |  | 327.15 | 0.1-59.59 |  | 0.0693 | 0.2129 | -0.0693 |  |
|  |  | 329.15 | 0.1-59.59 |  | 0.0734 | 0.2298 | -0.0722 |  |
|  |  | 331.15 | 0.1-59.59 |  | 0.0829 | 0.2465 | -0.0820 |  |
|  |  | 333.15 | 0.1-59.59 |  | 0.0857 | 0.2590 | -0.0849 |  |
| [C_3_mpip][NTf_2_] | REF 45 | 293.15 | 0.1-35 | for  T = 333.15 K  and  p = 15 MPa  0.05 % | 0.0147 | 0.0226 | 0.0147 | Isothermal Compressibility from REF 45 |
|  |  | 303.15 | 0.1-35 |  | 0.0085 | 0.0110 | -0.0085 |  |
|  |  | 313.15 | 0.1-35 |  | 0.0180 | 0.0272 | -0.0180 |  |
|  |  | 323.15 | 0.1-35 |  | 0.0251 | 0.0463 | -0.0063 |  |
|  |  | 333.15 | 0.1-35 |  | 0.0421 | 0.0516 | 0.0421 |  |
|  |  | 353.15 | 0.1-35 |  | 0.0156 | 0.0439 | 0.0146 |  |
|  |  | 393.15 | 0.1-35 |  | 0.0160 | 0.0372 | 0.0013 |  |
| [C_3_mpip][NTf_2_] | REF 59 | 298.15 | 0.1-50 | for  T = 348.15 K  and  p = 50 MPa  0.27 % | 0.0551 | 0.0903 | 0.0551 | Isothermal Compressibility REF 45 |
|  |  | 318.15 | 0.1-50 |  | 0.0996 | 0.1705 | 0.0996 |  |
|  |  | 348.15 | 0.1-50 |  | 0.1553 | 0.2713 | 0.1553 |  |
| [C_3_mpyrro][NTf_2_] | REF 45 | 293.15 | 0.1-35 | for  T = 393.15 K  and  p = 35 MPa  0.02 % | 0.0058 | 0.0149 | -0.0042 | Isothermal Compressibility from REF 45 |
|  |  | 303.15 | 0.1-35 |  | 0.0058 | 0.0112 | -0.0058 |  |
|  |  | 313.15 | 0.1-35 |  | 0.0047 | 0.0133 | -0.0044 |  |
|  |  | 323.15 | 0.1-35 |  | 0.0088 | 0.0146 | 0.0088 |  |
|  |  | 333.15 | 0.1-35 |  | 0.0056 | 0.0138 | 0.0056 |  |
|  |  | 353.15 | 0.1-35 |  | 0.0056 | 0.0099 | -0.0024 |  |
|  |  | 393.15 | 0.1-35 |  | 0.0104 | 0.0245 | 0.0104 |  |
| [C_3_py][BF_4_] | REF 75 | 283.15 | 0.1-65 | for  T = 328.15 K  and  p = 65 MPa  0.08 % | 0.0205 | 0.0597 | 0.0192 | Isothermal Compressibility from REF 75 |
|  |  | 288.15 | 0.1-65 |  | 0.0267 | 0.0638 | 0.0267 |  |
|  |  | 293.15 | 0.1-65 |  | 0.0307 | 0.0707 | 0.0307 |  |
|  |  | 298.15 | 0.1-65 |  | 0.0227 | 0.0608 | 0.0227 |  |
|  |  | 303.15 | 0.1-65 |  | 0.0217 | 0.0616 | 0.0189 |  |
|  |  | 308.15 | 0.1-65 |  | 0.0245 | 0.0652 | 0.0224 |  |
|  |  | 313.15 | 0.1-65 |  | 0.0160 | 0.0559 | 0.0143 |  |
|  |  | 318.15 | 0.1-65 |  | 0.0320 | 0.0732 | 0.0316 |  |
|  |  | 323.15 | 0.1-65 |  | 0.0216 | 0.0617 | 0.0209 |  |
|  |  | 328.15 | 0.1-65 |  | 0.0363 | 0.0768 | 0.0363 |  |
|  |  | 333.15 | 0.1-65 |  | 0.0303 | 0.0711 | 0.0303 |  |
| [C_4_C_1_mim][(C_2_F_5_)_3_PF_3_] | REF 72 | 278.15 | 0.1-120 | for  T = 278.15 K  and  p = 120 MPa  0.16 % | 0.0414 | 0.0929 | -0.0095 | Dataset used: density corrected (viscosity issue); Isothermal Compressibility calculated using the GCM method proposed in REF 53 |
|  |  | 298.15 | 0.1-120 |  | 0.0480 | 0.0770 | 0.0166 |  |
|  |  | 313.15 | 0.1-120 |  | 0.0284 | 0.0633 | 0.0034 |  |
|  |  | 333.15 | 0.1-120 |  | 0.0215 | 0.0601 | -0.0130 |  |
|  |  | 348.15 | 0.1-120 |  | 0.0273 | 0.0653 | -0.0271 |  |
|  |  | 373.15 | 0.1-120 |  | 0.0090 | 0.0235 | -0.0037 |  |
|  |  | 398.15 | 0.1-120 |  | 0.0218 | 0.0302 | 0.0037 |  |
|  |  | 278.15 | 0.1-120 |  | 0.0847 | 0.1636 | -0.0847 |  |
|  |  | 298.15 | 0.1-120 |  | 0.0572 | 0.1538 | -0.0526 |  |
|  |  | 313.15 | 0.1-120 |  | 0.0519 | 0.1353 | -0.0505 |  |
|  |  | 333.15 | 0.1-120 |  | 0.0502 | 0.1207 | -0.0502 |  |
|  |  | 348.15 | 0.1-120 |  | 0.0535 | 0.1080 | -0.0535 |  |
|  |  | 373.15 | 0.1-120 |  | 0.0237 | 0.0420 | -0.0214 |  |
|  |  | 398.15 | 0.1-120 |  | 0.0190 | 0.0358 | -0.0085 |  |
| [C_4_C_1_mim][NTf_2_] | REF 72 | 278.15 | 0.1-120 | for  T = 298.15 K  and  p = 120 MPa  0.17 % | 0.0417 | 0.0879 | 0.0020 | Dataset used: density corrected (viscosity issue); IsothermalCompressibility calculated by using the GCM method proposed in REF 53 |
|  |  | 298.15 | 0.1-120 |  | 0.0430 | 0.0954 | 0.0060 |  |
|  |  | 313.15 | 0.1-120 |  | 0.0306 | 0.0803 | -0.0051 |  |
|  |  | 333.15 | 0.1-120 |  | 0.0279 | 0.0902 | -0.0162 |  |
|  |  | 348.15 | 0.1-120 |  | 0.0276 | 0.0858 | -0.0264 |  |
|  |  | 373.15 | 0.1-120 |  | 0.0190 | 0.0518 | -0.0134 |  |
|  |  | 398.15 | 0.1-120 |  | 0.0146 | 0.0325 | -0.0136 |  |
|  |  | 278.15 | 0.1-120 |  | 0.0738 | 0.1611 | -0.0738 |  |
|  |  | 298.15 | 0.1-120 |  | 0.0589 | 0.1694 | -0.0552 |  |
|  |  | 313.15 | 0.1-120 |  | 0.0479 | 0.1413 | -0.0467 |  |
|  |  | 333.15 | 0.1-120 |  | 0.0429 | 0.1244 | -0.0427 |  |
|  |  | 348.15 | 0.1-120 |  | 0.0476 | 0.1202 | -0.0476 |  |
|  |  | 373.15 | 0.1-120 |  | 0.0309 | 0.0728 | -0.0286 |  |
|  |  | 398.15 | 0.1-120 |  | 0.0249 | 0.0412 | -0.0249 |  |
| [C_4_C_1_mim][PF_6_] | REF 24 | 313.15 | 0.1-10 | for  T = 313.15 K  and  p = 10 MPa  0.06 % | 0.0206 | 0.0556 | 0.0206 | Isothermal Compressibility calculated from Tait equation from REF 24 |
|  |  | 323.15 | 0.1-10 |  | 0.0121 | 0.0175 | -0.0079 |  |
|  |  | 333.15 | 0.1-10 |  | 0.0165 | 0.0439 | 0.0165 |  |
|  |  | 343.15 | 0.1-10 |  | 0.0134 | 0.0308 | 0.0134 |  |
|  |  | 353.15 | 0.1-10 |  | 0.0140 | 0.0357 | 0.0140 |  |
|  |  | 363.15 | 0.1-10 |  | 0.0152 | 0.0285 | 0.0152 |  |
|  |  | 373.15 | 0.1-10 |  | 0.0200 | 0.0257 | -0.0196 |  |
|  |  | 383.15 | 0.1-10 |  | 0.0131 | 0.0182 | -0.0107 |  |
|  |  | 393.15 | 0.1-10 |  | 0.0269 | 0.0501 | 0.0269 |  |
| [C_4_mim][(C_2_F_5_)_3_PF_3_] | REF 68 | 353.05 | 0.1 | for  T = 293.18 K  and  p = 0.5 MPa  0.59 % | 0.0001 | 0.0001 | -0.0001 | Isothermal Compressibility and density calculated by using the GCM method proposed in REF 53 |
|  |  | 313.15 | 0.101 |  | 0.0071 | 0.0071 | -0.0071 |  |
|  |  | 323.19 | 0.101 |  | 0.0057 | 0.0057 | 0.0057 |  |
|  |  | 333.14 | 0.101 |  | 0.0012 | 0.0012 | -0.0012 |  |
|  |  | 343.14 | 0.101 |  | 0.0000 | 0.0000 | 0.0000 |  |
|  |  | 303.21 | 0.102 |  | 0.0032 | 0.0032 | 0.0032 |  |
|  |  | 293.18 | 0.105 |  | 0.0128 | 0.0128 | 0.0128 |  |
|  |  | 303.22 | 0.498 |  | 0.0013 | 0.0013 | 0.0013 |  |
|  |  | 333.11 | 0.499 |  | 0.0018 | 0.0018 | 0.0018 |  |
|  |  | 343.14 | 0.499 |  | 0.0016 | 0.0016 | 0.0016 |  |
|  |  | 293.18 | 0.5 |  | 0.5966 | 0.5966 | -0.5966 |  |
|  |  | 313.14 | 0.5 |  | 0.0067 | 0.0067 | -0.0067 |  |
|  |  | 323.21 | 0.5 |  | 0.0047 | 0.0047 | 0.0047 |  |
|  |  | 353.04 | 0.5 |  | 0.0030 | 0.0030 | 0.0030 |  |
|  |  | 293.18 | 1 |  | 0.0149 | 0.0149 | 0.0149 |  |
|  |  | 303.23 | 1 |  | 0.0005 | 0.0005 | -0.0005 |  |
|  |  | 313.16 | 1 |  | 0.0086 | 0.0086 | -0.0086 |  |
|  |  | 323.21 | 1 |  | 0.0051 | 0.0051 | 0.0051 |  |
|  |  | 333.08 | 1 |  | 0.0011 | 0.0011 | -0.0011 |  |
|  |  | 343.14 | 1 |  | 0.0026 | 0.0026 | -0.0026 |  |
|  |  | 353.05 | 1 |  | 0.0012 | 0.0012 | -0.0012 |  |
|  |  | 293.18 | 2.5 |  | 0.0206 | 0.0206 | 0.0206 |  |
|  |  | 303.26 | 2.5 |  | 0.0055 | 0.0055 | 0.0055 |  |
|  |  | 313.18 | 2.5 |  | 0.0055 | 0.0055 | -0.0055 |  |
|  |  | 323.2 | 2.5 |  | 0.0002 | 0.0002 | 0.0002 |  |
|  |  | 333.08 | 2.5 |  | 0.0042 | 0.0042 | -0.0042 |  |
|  |  | 343.14 | 2.5 |  | 0.0095 | 0.0095 | -0.0095 |  |
|  |  | 353.04 | 2.5 |  | 0.0114 | 0.0114 | -0.0114 |  |
|  |  | 293.18 | 5 |  | 0.0204 | 0.0204 | 0.0204 |  |
|  |  | 303.26 | 5 |  | 0.0094 | 0.0094 | 0.0094 |  |
|  |  | 313.18 | 5 |  | 0.0100 | 0.0100 | -0.0100 |  |
|  |  | 323.2 | 5 |  | 0.0002 | 0.0002 | -0.0002 |  |
|  |  | 333.05 | 5 |  | 0.0111 | 0.0111 | -0.0111 |  |
|  |  | 343.13 | 5 |  | 0.0137 | 0.0137 | -0.0137 |  |
|  |  | 353.04 | 5 |  | 0.0186 | 0.0186 | -0.0186 |  |
|  |  | 293.18 | 10 |  | 0.0271 | 0.0271 | 0.0271 |  |
|  |  | 303.26 | 10 |  | 0.0118 | 0.0118 | 0.0118 |  |
|  |  | 313.18 | 10 |  | 0.0060 | 0.0060 | -0.0060 |  |
|  |  | 323.2 | 10 |  | 0.0007 | 0.0007 | -0.0007 |  |
|  |  | 333.04 | 10 |  | 0.0093 | 0.0093 | -0.0093 |  |
|  |  | 343.13 | 10 |  | 0.0172 | 0.0172 | -0.0172 |  |
|  |  | 353.03 | 10 |  | 0.0262 | 0.0262 | -0.0262 |  |
|  |  | 293.18 | 15 |  | 0.0335 | 0.0335 | 0.0335 |  |
|  |  | 303.26 | 15 |  | 0.0196 | 0.0196 | 0.0196 |  |
|  |  | 313.18 | 15 |  | 0.0033 | 0.0033 | 0.0033 |  |
|  |  | 323.2 | 15 |  | 0.0038 | 0.0038 | 0.0038 |  |
|  |  | 333.04 | 15 |  | 0.0098 | 0.0098 | -0.0098 |  |
|  |  | 343.14 | 15 |  | 0.0231 | 0.0231 | -0.0231 |  |
|  |  | 353.03 | 15 |  | 0.0302 | 0.0302 | -0.0302 |  |
|  |  | 293.18 | 20 |  | 0.0457 | 0.0457 | 0.0457 |  |
|  |  | 303.27 | 20 |  | 0.0264 | 0.0264 | 0.0264 |  |
|  |  | 313.19 | 20 |  | 0.0112 | 0.0112 | 0.0112 |  |
|  |  | 323.22 | 20 |  | 0.0060 | 0.0060 | 0.0060 |  |
|  |  | 333.03 | 20 |  | 0.0045 | 0.0045 | -0.0045 |  |
|  |  | 343.14 | 20 |  | 0.0236 | 0.0236 | -0.0236 |  |
|  |  | 353.02 | 20 |  | 0.0354 | 0.0354 | -0.0354 |  |
|  |  | 293.18 | 25 |  | 0.0581 | 0.0581 | 0.0581 |  |
|  |  | 303.28 | 25 |  | 0.0392 | 0.0392 | 0.0392 |  |
|  |  | 313.2 | 25 |  | 0.0188 | 0.0188 | 0.0188 |  |
|  |  | 323.23 | 25 |  | 0.0208 | 0.0208 | 0.0208 |  |
|  |  | 333.02 | 25 |  | 0.0001 | 0.0001 | -0.0001 |  |
|  |  | 343.14 | 25 |  | 0.0189 | 0.0189 | -0.0189 |  |
|  |  | 353.02 | 25 |  | 0.0299 | 0.0299 | -0.0299 |  |
| [C_4_mim][DCA] | REF 15 | 293.15 | 0.1-60 | for  T = 393.15 K  and  p = 60 MPa  0.03 % | 0.0035 | 0.0078 | 0.0015 | Isothermal Compressibility from REF 15 |
|  |  | 303.15 | 0.1-60 |  | 0.0073 | 0.0140 | 0.0072 |  |
|  |  | 313.15 | 0.1-60 |  | 0.0078 | 0.0147 | 0.0078 |  |
|  |  | 323.15 | 0.1-60 |  | 0.0048 | 0.0128 | 0.0045 |  |
|  |  | 333.15 | 0.1-60 |  | 0.0352 | 0.5625 | 0.0331 |  |
|  |  | 343.15 | 0.1-60 |  | 0.0047 | 0.0147 | 0.0013 |  |
|  |  | 353.15 | 0.1-60 |  | 0.0045 | 0.0138 | 0.0010 |  |
|  |  | 363.15 | 0.1-60 |  | 0.0063 | 0.0129 | 0.0003 |  |
|  |  | 373.15 | 0.1-60 |  | 0.0064 | 0.0165 | 0.0021 |  |
|  |  | 393.15 | 0.1-60 |  | 0.0108 | 0.0326 | 0.0106 |  |
| [C_4_mim][DCA] | REF 76 | 283.13 | 1.903-5.035 | for  T = 393.17 K  and  p = 1.948 MPa  0.16 % | 0.0319 | 0.0339 | -0.0319 | Isothermal Compressibility from REF 15 |
|  |  | 283.15 | 9.948-99.916 |  | 0.0578 | 0.1071 | -0.0578 |  |
|  |  | 283.16 | 0.1-39.59 |  | 0.0088 | 0.0143 | -0.0088 |  |
|  |  | 293.14 | 2.517 |  | 0.0394 | 0.0394 | -0.0394 |  |
|  |  | 293.15 | 9.841 |  | 0.0299 | 0.0299 | -0.0299 |  |
|  |  | 293.16 | 0.1-98.971 |  | 0.0559 | 0.1181 | -0.0547 |  |
|  |  | 293.17 | 5.25-79.759 |  | 0.0462 | 0.0826 | -0.0462 |  |
|  |  | 298.11 | 1.527 |  | 0.0515 | 0.0515 | -0.0515 |  |
|  |  | 298.13 | 0.1-5.031 |  | 0.0214 | 0.0409 | -0.0214 |  |
|  |  | 298.14 | 98.948 |  | 0.1272 | 0.1272 | -0.1272 |  |
|  |  | 298.15 | 10.127-89.998 |  | 0.0625 | 0.1106 | -0.0625 |  |
|  |  | 298.16 | 49.997-59.652 |  | 0.0541 | 0.0601 | -0.0541 |  |
|  |  | 298.17 | 39.572 |  | 0.0328 | 0.0328 | -0.0328 |  |
|  |  | 303.14 | 0.1 |  | 0.0001 | 0.0001 | -0.0001 |  |
|  |  | 313.12 | 10.081-59.618 |  | 0.0690 | 0.0902 | -0.0690 |  |
|  |  | 313.14 | 39.539-49.994 |  | 0.0641 | 0.0698 | -0.0641 |  |
|  |  | 313.15 | 29.997-89.997 |  | 0.0912 | 0.1189 | -0.0912 |  |
|  |  | 313.16 | 0.1-99.676 |  | 0.0542 | 0.1262 | -0.0542 |  |
|  |  | 313.18 | 2.493-5.188 |  | 0.0275 | 0.0283 | -0.0275 |  |
|  |  | 323.14 | 0.1 |  | 0.0042 | 0.0042 | 0.0042 |  |
|  |  | 333.15 | 2.011-99.101 |  | 0.0591 | 0.1037 | -0.0591 |  |
|  |  | 333.16 | 0.1-59.702 |  | 0.0294 | 0.0667 | -0.0294 |  |
|  |  | 343.14 | 0.1 |  | 0.0025 | 0.0025 | 0.0025 |  |
|  |  | 353.12 | 19.804 |  | 0.0083 | 0.0083 | 0.0083 |  |
|  |  | 353.14 | 0.949-40.069 |  | 0.0139 | 0.0282 | 0.0087 |  |
|  |  | 353.15 | 10.221-98.489 |  | 0.0299 | 0.0390 | -0.0155 |  |
|  |  | 353.16 | 5.13-79.923 |  | 0.0325 | 0.0344 | -0.0096 |  |
|  |  | 353.17 | 59.903 |  | 0.0170 | 0.0170 | -0.0170 |  |
|  |  | 373.13 | 1.946-39.714 |  | 0.0800 | 0.0951 | 0.0800 |  |
|  |  | 373.14 | 5.239 |  | 0.0972 | 0.0972 | 0.0972 |  |
|  |  | 373.15 | 29.958-98.898 |  | 0.0576 | 0.0750 | 0.0576 |  |
|  |  | 373.16 | 19.993-79.655 |  | 0.0569 | 0.0908 | 0.0569 |  |
|  |  | 393.14 | 59.675-99.002 |  | 0.1129 | 0.1335 | 0.1129 |  |
|  |  | 393.15 | 5.173-89.994 |  | 0.1186 | 0.1531 | 0.1186 |  |
|  |  | 393.16 | 10.149-39.93 |  | 0.1328 | 0.1511 | 0.1328 |  |
|  |  | 393.17 | 1.948 |  | 0.1636 | 0.1636 | 0.1636 |  |
| [C_4_mim][DCA] | REF 9 | 237.204 | 1.3279 | for  T = 237.204 K  and  p = 1.3279 MPa  0.25 % | 0.2500 | 0.2500 | 0.2500 | Density (p = 0.1 MPa), Isothermal Compressibility from REF 15 |
|  |  | 242.136 | 9.9869 |  | 0.2425 | 0.2425 | 0.2425 |  |
|  |  | 247.769 | 20.189 |  | 0.2462 | 0.2462 | 0.2462 |  |
|  |  | 248.088 | 1.1705 |  | 0.2410 | 0.2410 | 0.2410 |  |
|  |  | 253.328 | 10.197 |  | 0.2399 | 0.2399 | 0.2399 |  |
|  |  | 253.334 | 29.961 |  | 0.2409 | 0.2409 | 0.2409 |  |
|  |  | 259.122 | 20.009 |  | 0.2343 | 0.2343 | 0.2343 |  |
|  |  | 259.172 | 39.931 |  | 0.2286 | 0.2286 | 0.2286 |  |
|  |  | 259.682 | 1.03 |  | 0.2322 | 0.2322 | 0.2322 |  |
|  |  | 265.123 | 50.114 |  | 0.2187 | 0.2187 | 0.2187 |  |
|  |  | 265.239 | 10.141 |  | 0.2283 | 0.2283 | 0.2283 |  |
|  |  | 265.249 | 30.347 |  | 0.2293 | 0.2293 | 0.2293 |  |
|  |  | 270.553 | 18.709 |  | 0.2214 | 0.2214 | 0.2214 |  |
|  |  | 271.066 | 59.859 |  | 0.1994 | 0.1994 | 0.1994 |  |
|  |  | 271.134 | 39.925 |  | 0.2160 | 0.2160 | 0.2160 |  |
|  |  | 273.279 | 2.7675 |  | 0.2215 | 0.2215 | 0.2215 |  |
|  |  | 277.415 | 50.079 |  | 0.2022 | 0.2022 | 0.2022 |  |
|  |  | 277.917 | 9.9551 |  | 0.2168 | 0.2168 | 0.2168 |  |
|  |  | 277.964 | 30.503 |  | 0.2109 | 0.2109 | 0.2109 |  |
|  |  | 277.981 | 10.008 |  | 0.2148 | 0.2148 | 0.2148 |  |
|  |  | 283.668 | 59.955 |  | 0.1836 | 0.1836 | 0.1836 |  |
|  |  | 284.145 | 40.138 |  | 0.1977 | 0.1977 | 0.1977 |  |
|  |  | 284.154 | 1.0388 |  | 0.2116 | 0.2116 | 0.2116 |  |
|  |  | 284.23 | 1.1483 |  | 0.2109 | 0.2109 | 0.2109 |  |
|  |  | 284.466 | 20.074 |  | 0.2102 | 0.2102 | 0.2102 |  |
|  |  | 291.052 | 29.671-30.032 |  | 0.2117 | 0.2225 | 0.2117 |  |
|  |  | 291.056 | 11.299 |  | 0.2046 | 0.2046 | 0.2046 |  |
|  |  | 291.445 | 51.356 |  | 0.1808 | 0.1808 | 0.1808 |  |
|  |  | 296.97 | 19.796 |  | 0.1914 | 0.1914 | 0.1914 |  |
|  |  | 297.265 | 60.158 |  | 0.1657 | 0.1657 | 0.1657 |  |
|  |  | 298.179 | 40.27 |  | 0.2091 | 0.2091 | 0.2091 |  |
|  |  | 298.184 | 40.659 |  | 0.1868 | 0.1868 | 0.1868 |  |
|  |  | 299.21 | 3.7191 |  | 0.1961 | 0.1961 | 0.1961 |  |
|  |  | 303.971 | 30.176 |  | 0.1897 | 0.1897 | 0.1897 |  |
|  |  | 303.972 | 10.315 |  | 0.1864 | 0.1864 | 0.1864 |  |
|  |  | 304.811 | 50325 |  | 0.1715 | 0.1715 | 0.1715 |  |
|  |  | 310.98 | 40.117 |  | 0.1764 | 0.1764 | 0.1764 |  |
|  |  | 311.013 | 20.033 |  | 0.1737 | 0.1737 | 0.1737 |  |
|  |  | 311.344 | 59.7 |  | 0.1547 | 0.1547 | 0.1547 |  |
|  |  | 311.367 | 0.6766 |  | 0.1826 | 0.1826 | 0.1826 |  |
|  |  | 317.984 | 49.917 |  | 0.1626 | 0.1626 | 0.1626 |  |
|  |  | 318.267 | 9.911 |  | 0.1755 | 0.1755 | 0.1755 |  |
|  |  | 318.275 | 30.183 |  | 0.1695 | 0.1695 | 0.1695 |  |
|  |  | 325.32 | 60.007 |  | 0.1462 | 0.1462 | 0.1462 |  |
|  |  | 325.555 | 39.796 |  | 0.1488 | 0.1488 | 0.1488 |  |
|  |  | 326.005 | 19.921 |  | 0.1578 | 0.1578 | 0.1578 |  |
|  |  | 326.531 | 0.8763 |  | 0.1673 | 0.1673 | 0.1673 |  |
|  |  | 333.119 | 50.157 |  | 0.1466 | 0.1466 | 0.1466 |  |
|  |  | 333.667 | 9.9181 |  | 0.1600 | 0.1600 | 0.1600 |  |
|  |  | 333.67 | 30.169 |  | 0.1581 | 0.1581 | 0.1581 |  |
|  |  | 340.796 | 60.149 |  | 0.1305 | 0.1305 | 0.1305 |  |
|  |  | 341.31 | 39.98 |  | 0.1485 | 0.1485 | 0.1485 |  |
|  |  | 341.769 | 20.126 |  | 0.1557 | 0.1557 | 0.1557 |  |
|  |  | 342.411 | 0.7815 |  | 0.1624 | 0.1624 | 0.1624 |  |
|  |  | 349.322 | 50.089 |  | 0.1376 | 0.1376 | 0.1376 |  |
|  |  | 349.802 | 9.6292 |  | 0.1566 | 0.1566 | 0.1566 |  |
|  |  | 349.804 | 30.031 |  | 0.1182 | 0.1182 | 0.1182 |  |
| [C_4_mim][BF_4_] | REF 21 | 278.15 | 0.1 | for  T = 333.15 K  and  p = 60 MPa  0.10 % | 0.0080 | 0.0080 | -0.0080 | Isothermal Compressibility from REF 22 |
|  |  | 283.15 | 0.1 |  | 0.0025 | 0.0025 | -0.0025 |  |
|  |  | 288.15 | 0.1 |  | 0.0096 | 0.0096 | 0.0096 |  |
|  |  | 293.15 | 0.1 |  | 0.0160 | 0.0160 | 0.0160 |  |
|  |  | 298.15 | 0.1 |  | 0.0254 | 0.0254 | 0.0254 |  |
|  |  | 303.15 | 0.1 |  | 0.0317 | 0.0317 | 0.0317 |  |
|  |  | 313.15 | 0.1 |  | 0.0028 | 0.0028 | -0.0028 |  |
|  |  | 323.15 | 0.1 |  | 0.0162 | 0.0162 | -0.0162 |  |
|  |  | 333.15 | 0.1 |  | 0.0107 | 0.0107 | 0.0107 |  |
|  |  | 298.15 | 0.1-60 |  | 0.0276 | 0.0425 | -0.0276 |  |
|  |  | 303.15 | 0.1-60 |  | 0.0126 | 0.0214 | -0.0023 |  |
|  |  | 313.15 | 10-60 |  | 0.0478 | 0.0605 | 0.0478 |  |
|  |  | 323.15 | 10-60 |  | 0.0340 | 0.0490 | 0.0340 |  |
|  |  | 333.15 | 10-60 |  | 0.0718 | 0.0998 | 0.0718 |  |
| [C_4_mim][BF_4_] | REF 23 | 293.15 | 0.1-20 | for  T = 313.15 K  and  p = 20 MPa  0.14 % | 0.0438 | 0.0839 | 0.0422 | Isothermal Compressibility from REF 22 |
|  |  | 313.15 | 0.1-20 |  | 0.0779 | 0.1383 | 0.0779 |  |
|  |  | 333.15 | 0.1-20 |  | 0.0297 | 0.0542 | -0.0297 |  |
|  |  | 353.15 | 0.1-20 |  | 0.0246 | 0.0531 | -0.0135 |  |
| [C_4_mim][BF_4_] | REF 24 | 293.15 | 0.1-10 | for  T = 343.15 K  and  p = 10 MPa  0.03 % | 0.0048 | 0.0143 | 0.0044 | Isothermal Compressibility from REF 22 |
|  |  | 303.15 | 0.1-10 |  | 0.0053 | 0.0114 | -0.0022 |  |
|  |  | 313.15 | 0.1-10 |  | 0.0054 | 0.0098 | -0.0041 |  |
|  |  | 323.15 | 0.1-10 |  | 0.0027 | 0.0133 | 0.0027 |  |
|  |  | 333.15 | 0.1-10 |  | 0.0255 | 0.0334 | -0.0255 |  |
|  |  | 343.15 | 0.1-10 |  | 0.0108 | 0.0336 | 0.0096 |  |
|  |  | 353.15 | 0.1-10 |  | 0.0050 | 0.0109 | 0.0023 |  |
|  |  | 363.15 | 0.1-10 |  | 0.0077 | 0.0129 | -0.0075 |  |
|  |  | 373.15 | 0.1-10 |  | 0.0036 | 0.0067 | -0.0023 |  |
|  |  | 383.15 | 0.1-10 |  | 0.0054 | 0.0116 | -0.0044 |  |
|  |  | 393.15 | 0.1-10 |  | 0.0165 | 0.0296 | -0.0165 |  |
| [C_4_mim][BF_4_] | REF 26 | 292.94 | 0.1 | for  T = 352.33 K  and  p = 40 MPa  0.15 % | 0.0187 | 0.0187 | -0.0187 | Isothermal Compressibility from REF 22 |
|  |  | 302.8 | 0.1 |  | 0.0031 | 0.0031 | -0.0031 |  |
|  |  | 312.79 | 0.1 |  | 0.0052 | 0.0052 | 0.0052 |  |
|  |  | 322.83 | 0.1 |  | 0.0109 | 0.0109 | 0.0109 |  |
|  |  | 332.53 | 0.1 |  | 0.0210 | 0.0210 | 0.0210 |  |
|  |  | 342.72 | 0.1 |  | 0.0099 | 0.0099 | 0.0099 |  |
|  |  | 352.32 | 0.1 |  | 0.0098 | 0.0098 | 0.0098 |  |
|  |  | 373.32 | 0.1 |  | 0.0146 | 0.0146 | -0.0146 |  |
|  |  | 391.29 | 0.1 |  | 0.0579 | 0.0579 | -0.0579 |  |
|  |  | 414.92 | 0.1 |  | 0.0374 | 0.0374 | 0.0374 |  |
|  |  | 293.5 | 1 |  | 0.0088 | 0.0088 | -0.0088 |  |
|  |  | 322.31 | 1 |  | 0.0216 | 0.0216 | 0.0216 |  |
|  |  | 352.33 | 1 |  | 0.0160 | 0.0160 | 0.0160 |  |
|  |  | 373.34 | 1 |  | 0.0075 | 0.0075 | -0.0075 |  |
|  |  | 391.27 | 1 |  | 0.0559 | 0.0559 | -0.0559 |  |
|  |  | 414.93 | 1 |  | 0.0297 | 0.0297 | 0.0297 |  |
|  |  | 293.51 | 5 |  | 0.0043 | 0.0043 | -0.0043 |  |
|  |  | 322.33 | 5 |  | 0.0366 | 0.0366 | 0.0366 |  |
|  |  | 352.32 | 5 |  | 0.0264 | 0.0264 | 0.0264 |  |
|  |  | 373.31 | 5 |  | 0.0019 | 0.0019 | 0.0019 |  |
|  |  | 391.31 | 5 |  | 0.0459 | 0.0459 | -0.0459 |  |
|  |  | 414.92 | 5 |  | 0.0302 | 0.0302 | 0.0302 |  |
|  |  | 293.49 | 10 |  | 0.0206 | 0.0206 | 0.0206 |  |
|  |  | 322.3 | 10 |  | 0.0509 | 0.0509 | 0.0509 |  |
|  |  | 352.31 | 10 |  | 0.0442 | 0.0442 | 0.0442 |  |
|  |  | 373.32 | 10 |  | 0.0095 | 0.0095 | 0.0095 |  |
|  |  | 391.29 | 10 |  | 0.0301 | 0.0301 | -0.0301 |  |
|  |  | 414.93 | 10 |  | 0.0420 | 0.0420 | 0.0420 |  |
|  |  | 293.5 | 20 |  | 0.0487 | 0.0487 | 0.0487 |  |
|  |  | 322.31 | 20 |  | 0.0898 | 0.0898 | 0.0898 |  |
|  |  | 352.32 | 20 |  | 0.0842 | 0.0842 | 0.0842 |  |
|  |  | 373.31 | 20 |  | 0.0505 | 0.0505 | 0.0505 |  |
|  |  | 391.29 | 20 |  | 0.0028 | 0.0028 | -0.0028 |  |
|  |  | 414.92 | 20 |  | 0.0665 | 0.0665 | 0.0665 |  |
|  |  | 293.49 | 30 |  | 0.0813 | 0.0813 | 0.0813 |  |
|  |  | 322.31 | 30 |  | 0.1238 | 0.1238 | 0.1238 |  |
|  |  | 352.32 | 30 |  | 0.1110 | 0.1110 | 0.1110 |  |
|  |  | 373.33 | 30 |  | 0.0760 | 0.0760 | 0.0760 |  |
|  |  | 391.29 | 30 |  | 0.0287 | 0.0287 | 0.0287 |  |
|  |  | 414.93 | 30 |  | 0.0949 | 0.0949 | 0.0949 |  |
|  |  | 293.49 | 40 |  | 0.1010 | 0.1010 | 0.1010 |  |
|  |  | 322.3 | 40 |  | 0.1457 | 0.1457 | 0.1457 |  |
|  |  | 352.33 | 40 |  | 0.1488 | 0.1488 | 0.1488 |  |
|  |  | 373.33 | 40 |  | 0.1156 | 0.1156 | 0.1156 |  |
|  |  | 391.29 | 40 |  | 0.0650 | 0.0650 | 0.0650 |  |
|  |  | 414.92 | 40 |  | 0.1301 | 0.1301 | 0.1301 |  |
| [C_4_mim][BF_4_] | REF 29 | 313.1 | 0.1-200 | for  T = 452.3 K  and  p = 200 MPa  0.52 % | 0.0598 | 0.1204 | 0.0557 | Isothermal Compressibility from REF 22 |
|  |  | 332.6 | 0.1-200 |  | 0.1214 | 0.2062 | 0.1214 |  |
|  |  | 352.5 | 0.1-200 |  | 0.1892 | 0.3183 | 0.1892 |  |
|  |  | 372.7 | 0.1-200 |  | 0.1987 | 0.3709 | 0.1987 |  |
|  |  | 392.8 | 0.1-200 |  | 0.1649 | 0.3991 | 0.1498 |  |
|  |  | 412.9 | 0.1-200 |  | 0.1353 | 0.3853 | 0.1163 |  |
|  |  | 432.6 | 0.1-200 |  | 0.1662 | 0.4573 | 0.1240 |  |
|  |  | 452.3 | 0.1-200 |  | 0.1662 | 0.5192 | 0.1161 |  |
|  |  | 472.2 | 0.1-200 |  | 0.1646 | 0.4781 | 0.0201 |  |
| [C_4_mim][BF_4_] | REF 30 | 313.2 | 0.1-2 | for  T = 433.2 K  and  p = 2 Mpa  0.61 % | 0.3998 | 0.5686 | 0.3998 | Density (p = 0.1 MPa) from REF 26, Isothermal Compressibility from REF 22 |
|  |  | 353.2 | 0.2-2 |  | 0.1908 | 0.2183 | 0.1908 |  |
|  |  | 393.2 | 0.6-2 |  | 0.1018 | 0.1282 | -0.1018 |  |
|  |  | 433.2 | 1.4-2 |  | 0.5973 | 0.6102 | -0.5973 |  |
| [C_4_mim][BF_4_] | REF 31 | 298.15 | 0.1-30 | for  T = 323.15 K  and  p = 30 MPa  0.07 % | 0.0220 | 0.0631 | -0.0217 | Isothermal Compressibility from REF 22 |
|  |  | 323.15 | 0.1-30 |  | 0.0304 | 0.0722 | -0.0304 |  |
| [C_4_mim][BF_4_] | REF 32 | 283.15 | 0.1-40 | for  T = 323.15 K  and  p = 40 MPa  0.03 % | 0.0087 | 0.0213 | -0.0064 | Isothermal Compressibility from REF 22 |
|  |  | 293.15 | 0.1-40 |  | 0.0092 | 0.0263 | -0.0073 |  |
|  |  | 303.15 | 0.1-40 |  | 0.0113 | 0.0237 | -0.0107 |  |
|  |  | 313.15 | 0.1-40 |  | 0.0138 | 0.0240 | -0.0133 |  |
|  |  | 323.15 | 0.1-40 |  | 0.0136 | 0.0312 | -0.0136 |  |
|  |  | 333.15 | 0.1-40 |  | 0.0147 | 0.0268 | -0.0132 |  |
| [C_4_mim][BF_4_] | REF 34 | 283.15 | 0.1-35 | for  T = 353.15 K  and  p = 35 MPa  0.05 % | 0.0217 | 0.0359 | -0.0217 | Isothermal Compressibility from REF 22 |
|  |  | 285.65 | 0.1-35 |  | 0.0188 | 0.0332 | -0.0188 |  |
|  |  | 288.15 | 0.1-35 |  | 0.0162 | 0.0305 | -0.0162 |  |
|  |  | 290.65 | 0.1-35 |  | 0.0148 | 0.0286 | -0.0148 |  |
|  |  | 293.15 | 0.1-35 |  | 0.0112 | 0.0242 | -0.0112 |  |
|  |  | 295.65 | 0.1-35 |  | 0.0091 | 0.0222 | -0.0081 |  |
|  |  | 298.15 | 0.1-35 |  | 0.0079 | 0.0194 | -0.0066 |  |
|  |  | 300.65 | 0.1-35 |  | 0.0167 | 0.0368 | 0.0104 |  |
|  |  | 303.15 | 0.1-35 |  | 0.0025 | 0.0054 | -0.0008 |  |
|  |  | 305.65 | 0.1-35 |  | 0.0070 | 0.0141 | -0.0019 |  |
|  |  | 308.15 | 0.1-35 |  | 0.0074 | 0.0169 | -0.0042 |  |
|  |  | 310.65 | 0.1-35 |  | 0.0095 | 0.0206 | -0.0089 |  |
|  |  | 313.15 | 0.1-35 |  | 0.0084 | 0.0185 | -0.0071 |  |
|  |  | 315.65 | 0.1-35 |  | 0.0091 | 0.0205 | -0.0081 |  |
|  |  | 318.15 | 0.1-35 |  | 0.0158 | 0.0267 | -0.0158 |  |
|  |  | 320.65 | 0.1-35 |  | 0.0259 | 0.0379 | -0.0259 |  |
|  |  | 323.15 | 0.1-35 |  | 0.0241 | 0.0366 | -0.0241 |  |
|  |  | 325.65 | 0.1-35 |  | 0.0241 | 0.0378 | -0.0241 |  |
|  |  | 328.15 | 0.1-35 |  | 0.0264 | 0.0399 | -0.0264 |  |
|  |  | 330.65 | 0.1-35 |  | 0.0300 | 0.0436 | -0.0300 |  |
|  |  | 333.15 | 0.1-35 |  | 0.0295 | 0.0431 | -0.0295 |  |
|  |  | 335.65 | 0.1-35 |  | 0.0096 | 0.0184 | -0.0086 |  |
|  |  | 338.15 | 0.1-35 |  | 0.0069 | 0.0128 | -0.0020 |  |
|  |  | 340.65 | 0.1-35 |  | 0.0130 | 0.0267 | -0.0119 |  |
|  |  | 343.15 | 0.1-35 |  | 0.0093 | 0.0199 | 0.0067 |  |
|  |  | 345.65 | 0.1-35 |  | 0.0125 | 0.0190 | 0.0025 |  |
|  |  | 348.15 | 0.1-35 |  | 0.0186 | 0.0355 | -0.0184 |  |
|  |  | 350.65 | 0.1-35 |  | 0.0256 | 0.0410 | -0.0256 |  |
|  |  | 353.15 | 0.1-35 |  | 0.0290 | 0.0491 | -0.0290 |  |
| [C_4_mim][BF_4_] | REF 13 | 283.15 | 0.1-60 | for  T = 313.15 K  and  p = 14 MPa  0.05 % | 0.0054 | 0.0092 | 0.0035 | Isothermal Compressibility from REF 22 |
|  |  | 288.15 | 0.1-60 |  | 0.0033 | 0.0060 | 0.0027 |  |
|  |  | 293.15 | 0.1-60 |  | 0.0050 | 0.0095 | 0.0050 |  |
|  |  | 298.15 | 0.1-60 |  | 0.0074 | 0.0146 | 0.0074 |  |
|  |  | 303.15 | 0.1-60 |  | 0.0077 | 0.0150 | 0.0075 |  |
|  |  | 308.15 | 0.1-60 |  | 0.0094 | 0.0210 | 0.0094 |  |
|  |  | 313.15 | 0.1-60 |  | 0.0113 | 0.0483 | 0.0111 |  |
|  |  | 318.15 | 0.1-60 |  | 0.0067 | 0.0213 | 0.0064 |  |
|  |  | 323.15 | 0.1-60 |  | 0.0074 | 0.0240 | 0.0071 |  |
| [C_4_mim][BF_4_] | REF 22 | 298.34 | 0.1-59.23 | for  T = 332.73 K  and  p = 59.13 MPa  0.11 % | 0.0123 | 0.0253 | 0.0088 | Isothermal Compressibility from REF 22 |
|  |  | 303.23 | 0.1-59.82 |  | 0.0361 | 0.0463 | 0.0361 |  |
|  |  | 313.01 | 0.1-59.45 |  | 0.0688 | 0.0905 | 0.0685 |  |
|  |  | 322.85 | 0.1-59.92 |  | 0.0448 | 0.0691 | 0.0439 |  |
|  |  | 332.73 | 0.1-59.13 |  | 0.0633 | 0.1105 | 0.0633 |  |
| [C_4_mim][BF_4_] | REF 25 | 298.15 | 0.28-39.61 | for  T = 398.15 K  and  p = 39.56 MPa  1.22 % | 0.8184 | 0.8567 | -0.8184 | Density (p = 0.1 MPa) from REF 26, Isothermal Compressibility from REF 22 |
|  |  | 323.15 | 0.73-39.85 |  | 0.8121 | 0.8261 | -0.8121 |  |
|  |  | 348.15 | 0.21-39.61 |  | 0.8618 | 0.8933 | -0.8618 |  |
|  |  | 373.15 | 0.47-39.82 |  | 0.9703 | 1.0215 | -0.9703 |  |
|  |  | 398.15 | 0.87-39.56 |  | 1.1451 | 1.2317 | -1.1451 |  |
| [C_4_mim][BF_4_] | REF 27 | 273.15 | 0.1 | for  T = 323.15 K  and  p = 225.3 MPa  1.78 % | 0.0071 | 0.0128 | -0.0071 | Isothermal Compressibility from REF 22 |
|  |  | 278.15 | 0.1 |  | 0.0039 | 0.0043 | 0.0039 |  |
|  |  | 283.15 | 0.1-100.6 |  | 0.0538 | 0.1796 | -0.0489 |  |
|  |  | 288.15 | 0.1 |  | 0.0176 | 0.0185 | 0.0176 |  |
|  |  | 293.15 | 0.1 |  | 0.0113 | 0.0171 | 0.0113 |  |
|  |  | 298.15 | 0.1-200.9 |  | 0.1471 | 0.3100 | -0.1399 |  |
|  |  | 303.15 | 0.1 |  | 0.0163 | 0.0381 | 0.0150 |  |
|  |  | 313.15 | 0.1 |  | 0.0186 | 0.0475 | 0.0179 |  |
|  |  | 323.15 | 0.1-250.1 |  | 1.3483 | 1.8099 | -1.3471 |  |
|  |  | 333.15 | 0.1 |  | 0.0047 | 0.0054 | 0.0008 |  |
|  |  | 343.15 | 0.1 |  | 0.0038 | 0.0053 | 0.0027 |  |
|  |  | 348.15 | 0.1-300 |  | 0.2116 | 0.3066 | -0.2116 |  |
|  |  | 353.15 | 0.1 |  | 0.0077 | 0.0085 | 0.0077 |  |
|  |  | 360.15 | 0.1 |  | 0.0028 | 0.0028 | -0.0028 |  |
|  |  | 362.76 | 0.1 |  | 0.0034 | 0.0034 | -0.0034 |  |
|  |  | 363.15 | 0.1 |  | 0.0083 | 0.0083 | 0.0083 |  |
|  |  | 363.16 | 0.1 |  | 0.0018 | 0.0018 | -0.0018 |  |
| [C_4_mim][BF_4_] | REF 28 same data as REF 25 | 298.15 | 0.28-39.61 | for  T = 398.15 K  and  p = 39.56 MPa  1.22 % | 0.8184 | 0.8567 | -0.8184 | Density (p = 0.1 MPa) from REF 26, Isothermal Compressibility from REF 22 |
|  |  | 323.15 | 0.73-39.85 |  | 0.8121 | 0.8261 | -0.8121 |  |
|  |  | 348.15 | 0.21-39.61 |  | 0.8618 | 0.8933 | -0.8618 |  |
|  |  | 373.15 | 0.47-39.82 |  | 0.9703 | 1.0215 | -0.9703 |  |
|  |  | 398.15 | 0.87-39.56 |  | 1.1451 | 1.2317 | -1.1451 |  |
| [C_4_mim][BF_4_] | REF 33 | 239.754 | 1.8754 | for  T = 353.675 K  and  p = 59.697  0.13 % | 0.0090 | 0.0090 | 0.0090 | Isothermal Compressibility from REF 22 |
|  |  | 244.146 | 9.1872 |  | 0.0132 | 0.0132 | 0.0132 |  |
|  |  | 244.152 | 30.106 |  | 0.0238 | 0.0238 | -0.0238 |  |
|  |  | 250.803 | 41.033 |  | 0.0336 | 0.0336 | -0.0336 |  |
|  |  | 251.148 | 20.631 |  | 0.0141 | 0.0141 | 0.0141 |  |
|  |  | 251.624 | 1.4315 |  | 0.0529 | 0.0529 | 0.0529 |  |
|  |  | 256.789 | 51.047 |  | 0.0349 | 0.0349 | -0.0349 |  |
|  |  | 257.247 | 10.286 |  | 0.0532 | 0.0532 | 0.0532 |  |
|  |  | 257.259 | 30.54 |  | 0.0123 | 0.0123 | 0.0123 |  |
|  |  | 262.282 | 59.767 |  | 0.0484 | 0.0484 | -0.0484 |  |
|  |  | 263.492 | 40.569 |  | 0.0098 | 0.0098 | 0.0098 |  |
|  |  | 263.701 | 20.36 |  | 0.0518 | 0.0518 | 0.0518 |  |
|  |  | 264.468 | 1.1974 |  | 0.0436 | 0.0436 | 0.0436 |  |
|  |  | 269.793 | 50.298 |  | 0.0032 | 0.0032 | -0.0032 |  |
|  |  | 270.25 | 9.9638 |  | 0.0453 | 0.0453 | 0.0453 |  |
|  |  | 270.252 | 30.434 |  | 0.0464 | 0.0464 | 0.0464 |  |
|  |  | 275.567 | 59.083 |  | 0.0177 | 0.0177 | -0.0177 |  |
|  |  | 276.946 | 19.845 |  | 0.0386 | 0.0386 | 0.0386 |  |
|  |  | 276.968 | 40.555 |  | 0.0351 | 0.0351 | 0.0351 |  |
|  |  | 278.132 | 1.66 |  | 0.0508 | 0.0508 | 0.0508 |  |
|  |  | 283.484 | 50.231 |  | 0.0223 | 0.0223 | 0.0223 |  |
|  |  | 284.057 | 30.274 |  | 0.0301 | 0.0301 | 0.0301 |  |
|  |  | 284.095 | 10.163 |  | 0.0461 | 0.0461 | 0.0461 |  |
|  |  | 290.086 | 59.872 |  | 0.0055 | 0.0055 | 0.0055 |  |
|  |  | 291.266 | 40.686 |  | 0.0183 | 0.0183 | 0.0183 |  |
|  |  | 291.46 | 20.613 |  | 0.0379 | 0.0379 | 0.0379 |  |
|  |  | 292.092 | 1.4458-1.9404 |  | 0.0785 | 0.0884 | 0.0785 |  |
|  |  | 298.118 | 0.1059 |  | 0.0673 | 0.0673 | -0.0673 |  |
|  |  | 298.124 | 9.5508 |  | 0.0567 | 0.0567 | 0.0567 |  |
|  |  | 298.131 | 10.109 |  | 0.0784 | 0.0784 | 0.0784 |  |
|  |  | 298.138 | 10.428-29.887 |  | 0.0495 | 0.0735 | 0.0495 |  |
|  |  | 298.58 | 50.942 |  | 0.0027 | 0.0027 | -0.0027 |  |
|  |  | 305.026 | 19.306 |  | 0.0631 | 0.0631 | 0.0631 |  |
|  |  | 305.528 | 0.8986 |  | 0.0822 | 0.0822 | 0.0822 |  |
|  |  | 305.535 | 60.508 |  | 0.0263 | 0.0263 | -0.0263 |  |
|  |  | 305.667 | 40.148 |  | 0.0048 | 0.0048 | 0.0048 |  |
|  |  | 313.034 | 9.7903 |  | 0.0468 | 0.0468 | 0.0468 |  |
|  |  | 313.037 | 29.821 |  | 0.0399 | 0.0399 | 0.0399 |  |
|  |  | 313.038 | 30.149 |  | 0.0353 | 0.0353 | 0.0353 |  |
|  |  | 313.045 | 10.27 |  | 0.0519 | 0.0519 | 0.0519 |  |
|  |  | 313.058 | 29.28 |  | 0.0179 | 0.0179 | 0.0179 |  |
|  |  | 313.606 | 50.546 |  | 0.0278 | 0.0278 | -0.0278 |  |
|  |  | 320.097 | 0.3286 |  | 0.0415 | 0.0415 | 0.0415 |  |
|  |  | 320.513 | 19.693 |  | 0.0279 | 0.0279 | 0.0279 |  |
|  |  | 321.398 | 60.815 |  | 0.0553 | 0.0553 | -0.0553 |  |
|  |  | 321.613 | 40.89 |  | 0.0105 | 0.0105 | 0.0105 |  |
|  |  | 327.983 | 48.935 |  | 0.0164 | 0.0164 | -0.0164 |  |
|  |  | 328.175 | 28.863 |  | 0.0148 | 0.0148 | -0.0148 |  |
|  |  | 328.176 | 10.05 |  | 0.0094 | 0.0094 | 0.0094 |  |
|  |  | 328.178 | 29.263 |  | 0.0005 | 0.0005 | 0.0005 |  |
|  |  | 328.181 | 29.052 |  | 0.0056 | 0.0056 | 0.0056 |  |
|  |  | 328.224 | 10.05 |  | 0.0065 | 0.0065 | 0.0065 |  |
|  |  | 336.35 | 39.21 |  | 0.0362 | 0.0362 | -0.0362 |  |
|  |  | 336.681 | 20.276 |  | 0.0236 | 0.0236 | -0.0236 |  |
|  |  | 337.094 | 59.633 |  | 0.0812 | 0.0812 | -0.0812 |  |
|  |  | 344.203 | 29.113 |  | 0.0607 | 0.0607 | -0.0607 |  |
|  |  | 344.315 | 48.811 |  | 0.0743 | 0.0743 | -0.0743 |  |
|  |  | 353.675 | 59.697 |  | 0.1302 | 0.1302 | -0.1302 |  |
|  |  | 354.156 | 40.617 |  | 0.1146 | 0.1146 | -0.1146 |  |
| [C_4_mim][C(CN)_3_] | REF 11 | 293.15 | 0.1-30 | for  T = 393.15 K  and  p = 30 MPa  0.13 % | 0.0393 | 0.0914 | -0.0393 | Isothermal Compressibility from REF 11 |
|  |  | 303.15 | 0.1-30 |  | 0.0145 | 0.0386 | 0.0019 |  |
|  |  | 313.15 | 0.1-30 |  | 0.0207 | 0.0566 | 0.0028 |  |
|  |  | 323.15 | 0.1-30 |  | 0.0188 | 0.0797 | -0.0134 |  |
|  |  | 333.15 | 0.1-30 |  | 0.0218 | 0.0792 | -0.0072 |  |
|  |  | 343.15 | 0.1-30 |  | 0.0427 | 0.1129 | -0.0427 |  |
|  |  | 353.15 | 0.1-30 |  | 0.0303 | 0.0838 | -0.0303 |  |
|  |  | 393.15 | 0.1-30 |  | 0.0299 | 0.1284 | -0.0278 |  |
| [C_4_mim][C_8_SO_4_] | REF 29 | 312.9 | 0.1-90 | for  T = 472.6 K  and  p = 200 MPa  2.07 % | 0.5751 | 1.0555 | -0.5751 | Isothermal Compressibility from REF 29 |
|  |  | 332.8 | 0.1-200 |  | 0.8894 | 1.8249 | -0.8853 |  |
|  |  | 352.8 | 0.1-200 |  | 0.6058 | 1.3612 | -0.5894 |  |
|  |  | 373 | 0.1-200 |  | 0.3621 | 0.8721 | -0.3331 |  |
|  |  | 393.1 | 0.1-200 |  | 0.1384 | 0.3534 | -0.1125 |  |
|  |  | 413 | 0.1-200 |  | 0.0925 | 0.1809 | 0.0879 |  |
|  |  | 432.9 | 0.1-200 |  | 0.2990 | 0.6922 | 0.2830 |  |
|  |  | 452.7 | 0.1-200 |  | 0.5988 | 1.3477 | 0.5957 |  |
|  |  | 472.6 | 0.1-200 |  | 0.7971 | 2.0273 | 0.7971 |  |
| [C_4_mim][C_8_SO_4_] | REF 51 | 298.15 | 0.1 | for  T = 318.15 K  and  p = 50 MPa  0.41 % | 0.0637 | 0.0637 | -0.0637 | Isothermal Compressibility from REF 51 |
|  |  | 299.15 | 0.1 |  | 0.0599 | 0.0599 | -0.0599 |  |
|  |  | 300.15 | 0.1 |  | 0.0577 | 0.0577 | -0.0577 |  |
|  |  | 301.15 | 0.1 |  | 0.0545 | 0.0545 | -0.0545 |  |
|  |  | 302.15 | 0.1 |  | 0.0512 | 0.0512 | -0.0512 |  |
|  |  | 303.15 | 0.1 |  | 0.0476 | 0.0476 | -0.0476 |  |
|  |  | 304.15 | 0.1 |  | 0.0449 | 0.0449 | -0.0449 |  |
|  |  | 305.15 | 0.1 |  | 0.0410 | 0.0410 | -0.0410 |  |
|  |  | 306.15 | 0.1 |  | 0.0380 | 0.0380 | -0.0380 |  |
|  |  | 307.15 | 0.1 |  | 0.0338 | 0.0338 | -0.0338 |  |
|  |  | 308.15 | 0.1 |  | 0.0304 | 0.0304 | -0.0304 |  |
|  |  | 309.15 | 0.1 |  | 0.0269 | 0.0269 | -0.0269 |  |
|  |  | 310.15 | 0.1 |  | 0.0232 | 0.0232 | -0.0232 |  |
|  |  | 311.15 | 0.1 |  | 0.0184 | 0.0184 | -0.0184 |  |
|  |  | 312.15 | 0.1 |  | 0.0143 | 0.0143 | -0.0143 |  |
|  |  | 313.15 | 0.1 |  | 0.0101 | 0.0101 | -0.0101 |  |
|  |  | 314.15 | 0.1 |  | 0.0058 | 0.0058 | -0.0058 |  |
|  |  | 315.15 | 0.1 |  | 0.0013 | 0.0013 | -0.0013 |  |
|  |  | 316.15 | 0.1 |  | 0.0024 | 0.0024 | 0.0024 |  |
|  |  | 317.15 | 0.1 |  | 0.0073 | 0.0073 | 0.0073 |  |
|  |  | 318.15 | 0.1 |  | 0.0113 | 0.0113 | 0.0113 |  |
|  |  | 318.15 | 0.1 |  | 0.0113 | 0.0113 | 0.0113 |  |
|  |  | 319.15 | 0.1 |  | 0.0155 | 0.0155 | 0.0155 |  |
|  |  | 320.15 | 0.1 |  | 0.0208 | 0.0208 | 0.0208 |  |
|  |  | 321.15 | 0.1 |  | 0.0253 | 0.0253 | 0.0253 |  |
|  |  | 322.15 | 0.1 |  | 0.0291 | 0.0291 | 0.0291 |  |
|  |  | 323.15 | 0.1 |  | 0.0339 | 0.0339 | 0.0339 |  |
|  |  | 324.15 | 0.1 |  | 0.0389 | 0.0389 | 0.0389 |  |
|  |  | 325.15 | 0.1 |  | 0.0432 | 0.0432 | 0.0432 |  |
|  |  | 326.15 | 0.1 |  | 0.0476 | 0.0476 | 0.0476 |  |
|  |  | 327.15 | 0.1 |  | 0.0511 | 0.0511 | 0.0511 |  |
|  |  | 328.15 | 0.1 |  | 0.0539 | 0.0539 | 0.0539 |  |
|  |  | 328.15 | 0.1 |  | 0.0568 | 0.0568 | 0.0568 |  |
|  |  | 329.15 | 0.1 |  | 0.0569 | 0.0569 | 0.0569 |  |
|  |  | 330.15 | 0.1 |  | 0.0590 | 0.0590 | 0.0590 |  |
|  |  | 331.15 | 0.1 |  | 0.0614 | 0.0614 | 0.0614 |  |
|  |  | 332.15 | 0.1 |  | 0.0648 | 0.0648 | 0.0648 |  |
|  |  | 333.15 | 0.1 |  | 0.0684 | 0.0684 | 0.0684 |  |
|  |  | 334.15 | 0.1 |  | 0.0712 | 0.0712 | 0.0712 |  |
|  |  | 335.15 | 0.1 |  | 0.0741 | 0.0741 | 0.0741 |  |
|  |  | 336.15 | 0.1 |  | 0.0763 | 0.0763 | 0.0763 |  |
|  |  | 337.15 | 0.1 |  | 0.0805 | 0.0805 | 0.0805 |  |
|  |  | 338.15 | 0.1 |  | 0.0830 | 0.0830 | 0.0830 |  |
|  |  | 338.15 | 0.1 |  | 0.0840 | 0.0840 | 0.0840 |  |
|  |  | 339.15 | 0.1 |  | 0.0876 | 0.0876 | 0.0876 |  |
|  |  | 340.15 | 0.1 |  | 0.0943 | 0.0943 | 0.0943 |  |
|  |  | 341.15 | 0.1 |  | 0.0992 | 0.0992 | 0.0992 |  |
|  |  | 342.15 | 0.1 |  | 0.1042 | 0.1042 | 0.1042 |  |
|  |  | 343.15 | 0.1 |  | 0.1094 | 0.1094 | 0.1094 |  |
|  |  | 348.15 | 0.1 |  | 0.3566 | 0.3566 | -0.3566 |  |
|  |  | 358.15 | 0.1 |  | 0.3285 | 0.3285 | -0.3285 |  |
|  |  | 368.15 | 0.1 |  | 0.2742 | 0.2742 | -0.2742 |  |
|  |  | 378.15 | 0.1 |  | 0.2130 | 0.2130 | -0.2130 |  |
|  |  | 388.15 | 0.1 |  | 0.1447 | 0.1447 | -0.1447 |  |
|  |  | 398.15 | 0.1 |  | 0.0594 | 0.0594 | -0.0594 |  |
|  |  | 408.15 | 0.1 |  | 0.0132 | 0.0132 | 0.0132 |  |
|  |  | 418.15 | 0.1 |  | 0.0930 | 0.0930 | 0.0930 |  |
|  |  | 428.15 | 0.1 |  | 0.1699 | 0.1699 | 0.1699 |  |
|  |  | 318.15 | 1-60 |  | 0.3990 | 0.4153 | -0.3990 |  |
|  |  | 328.15 | 1-60 |  | 0.3774 | 0.4056 | -0.3774 |  |
|  |  | 338.15 | 1-60 |  | 0.3618 | 0.3890 | -0.3618 |  |
|  |  | 348.15 | 1-60 |  | 0.3275 | 0.3579 | -0.3275 |  |
|  |  | 358.15 | 1-60 |  | 0.2986 | 0.3179 | -0.2986 |  |
|  |  | 368.15 | 1-60 |  | 0.2478 | 0.2760 | -0.2478 |  |
|  |  | 378.15 | 1-60 |  | 0.1950 | 0.2076 | -0.1950 |  |
|  |  | 388.15 | 1-60 |  | 0.1454 | 0.1803 | -0.1454 |  |
|  |  | 398.15 | 1-60 |  | 0.0810 | 0.1195 | -0.0810 |  |
|  |  | 408.15 | 1-60 |  | 0.0342 | 0.0764 | -0.0260 |  |
|  |  | 418.15 | 1-60 |  | 0.0443 | 0.1015 | 0.0270 |  |
|  |  | 428.15 | 1-60 |  | 0.0871 | 0.1899 | 0.0871 |  |
| [C_4_mim][C_8_SO_4_] | REF 80 | 298.15 | 0.29-40.01 | for  T = 298.15 K  and  p = 40.01 MPa  0.46 % | 0.2125 | 0.4608 | -0.1969 | Density (p = 0.1 MPa), Isothermal Compressibility from REF 29 |
|  |  | 323.15 | 0.28-39.94 |  | 0.1029 | 0.2191 | 0.0370 |  |
|  |  | 348.15 | 0.48-39.95 |  | 0.1411 | 0.1813 | 0.1411 |  |
|  |  | 373.15 | 0.11-40.01 |  | 0.1576 | 0.1739 | 0.1576 |  |
|  |  | 398.15 | 0.33-40.03 |  | 0.0679 | 0.1307 | 0.0659 |  |
| [C_4_mim][C_1_SO_4_] | REF 34 | 283.15 | 0.1-35 | for  T = 338.15 K  and  p = 35 MPa  0.04 % | 0.0010 | 0.0020 | 0.0004 | Isothermal Compressibility from REF 34 |
|  |  | 285.65 | 0.1-35 |  | 0.0024 | 0.0041 | 0.0022 |  |
|  |  | 288.15 | 0.1-35 |  | 0.0034 | 0.0054 | 0.0034 |  |
|  |  | 290.65 | 0.1-35 |  | 0.0048 | 0.0070 | 0.0048 |  |
|  |  | 293.15 | 0.1-35 |  | 0.0019 | 0.0044 | -0.0019 |  |
|  |  | 295.65 | 0.1-35 |  | 0.0144 | 0.0155 | -0.0144 |  |
|  |  | 298.15 | 0.1-35 |  | 0.0056 | 0.0079 | -0.0056 |  |
|  |  | 300.65 | 0.1-35 |  | 0.0142 | 0.0208 | 0.0142 |  |
|  |  | 303.15 | 0.1-35 |  | 0.0051 | 0.0076 | -0.0051 |  |
|  |  | 305.65 | 0.1-35 |  | 0.0010 | 0.0040 | 0.0006 |  |
|  |  | 308.15 | 0.1-35 |  | 0.0011 | 0.0023 | 0.0009 |  |
|  |  | 310.65 | 0.1-35 |  | 0.0008 | 0.0017 | 0.0006 |  |
|  |  | 313.15 | 0.1-35 |  | 0.0017 | 0.0029 | 0.0017 |  |
|  |  | 315.65 | 0.1-35 |  | 0.0027 | 0.0037 | -0.0019 |  |
|  |  | 318.15 | 0.1-35 |  | 0.0066 | 0.0088 | -0.0066 |  |
|  |  | 320.65 | 0.1-35 |  | 0.0068 | 0.0089 | -0.0068 |  |
|  |  | 323.15 | 0.1-35 |  | 0.0081 | 0.0107 | -0.0081 |  |
|  |  | 325.65 | 0.1-35 |  | 0.0066 | 0.0082 | -0.0066 |  |
|  |  | 328.15 | 0.1-35 |  | 0.0060 | 0.0087 | -0.0060 |  |
|  |  | 330.65 | 0.1-35 |  | 0.0033 | 0.0067 | -0.0033 |  |
|  |  | 333.15 | 0.1-35 |  | 0.0045 | 0.0128 | -0.0045 |  |
|  |  | 335.65 | 0.1-35 |  | 0.0180 | 0.0253 | 0.0180 |  |
|  |  | 338.15 | 0.1-35 |  | 0.0349 | 0.0386 | 0.0349 |  |
|  |  | 340.65 | 0.1-35 |  | 0.0013 | 0.0043 | 0.0012 |  |
|  |  | 343.15 | 0.1-35 |  | 0.0216 | 0.0279 | 0.0216 |  |
|  |  | 345.65 | 0.1-35 |  | 0.0232 | 0.0266 | 0.0232 |  |
|  |  | 348.15 | 0.1-35 |  | 0.0079 | 0.0105 | 0.0079 |  |
|  |  | 350.65 | 0.1-35 |  | 0.0227 | 0.0242 | -0.0227 |  |
|  |  | 353.15 | 0.1-35 |  | 0.0205 | 0.0240 | -0.0205 |  |
| [C_4_mim][C_1_SO_4_] | REF 78 | 283.15 | 0.1-35 | for  T = 303.15 K  and  p = 30 MPa  0.04 % | 0.0039 | 0.0065 | 0.0018 | Isothermal Compressibility from REF 34 |
|  |  | 288.15 | 0.1-35 |  | 0.0040 | 0.0124 | 0.0009 |  |
|  |  | 293.15 | 0.1-35 |  | 0.0025 | 0.0040 | 0.0020 |  |
|  |  | 298.15 | 0.1-35 |  | 0.0081 | 0.0102 | -0.0081 |  |
|  |  | 303.15 | 0.1-35 |  | 0.0283 | 0.0436 | -0.0283 |  |
|  |  | 308.15 | 0.1-35 |  | 0.0065 | 0.0073 | 0.0065 |  |
|  |  | 313.15 | 0.1-35 |  | 0.0106 | 0.0127 | 0.0106 |  |
|  |  | 318.15 | 0.1-35 |  | 0.0153 | 0.0166 | 0.0153 |  |
|  |  | 323.15 | 0.1-35 |  | 0.0020 | 0.0033 | 0.0013 |  |
|  |  | 328.15 | 0.1-35 |  | 0.0044 | 0.0073 | -0.0044 |  |
|  |  | 333.15 | 0.1-35 |  | 0.0022 | 0.0113 | -0.0022 |  |
| [C_4_mim]Cl | REF 8 | 348.15 | 0.1 | for  T = 452 K  and  p = 10 MPa  1.23 % | 0.0007 | 0.0007 | 0.0007 | Isothermal Compressibility from REF 8 |
|  |  | 353.15 | 0.1 |  | 0.0009 | 0.0009 | 0.0009 |  |
|  |  | 358.15 | 0.1 |  | 0.0049 | 0.0049 | -0.0049 |  |
|  |  | 363.15 | 0.1 |  | 0.0022 | 0.0022 | 0.0022 |  |
|  |  | 368.15 | 0.1 |  | 0.0031 | 0.0031 | 0.0031 |  |
|  |  | 373.15 | 0.1 |  | 0.0021 | 0.0021 | -0.0021 |  |
|  |  | 352.1 | 10-200 |  | 0.1874 | 0.4606 | -0.1867 |  |
|  |  | 372.4 | 10-200 |  | 0.1459 | 0.4690 | -0.0676 |  |
|  |  | 392.3 | 10-200 |  | 0.2183 | 0.4120 | 0.1084 |  |
|  |  | 412.3 | 10-200 |  | 0.3263 | 0.6474 | 0.2811 |  |
|  |  | 432.2 | 10-200 |  | 0.4958 | 0.8973 | 0.4853 |  |
|  |  | 452 | 10-200 |  | 0.7762 | 1.2111 | 0.7762 |  |
| [C_4_mim][NTf_2_] | REF 15 | 293.15 | 0.1-59.97 | for  T = 453.15 K  and  p = 60 MPa  0.11 % | 0.0327 | 0.0392 | -0.0327 | Isothermal Compressibility from REF 15 |
|  |  | 303.15 | 0.1-60.01 |  | 0.0088 | 0.0129 | -0.0088 |  |
|  |  | 313.15 | 0.1-60 |  | 0.0112 | 0.0162 | 0.0112 |  |
|  |  | 323.15 | 0.1-60 |  | 0.0176 | 0.0219 | 0.0176 |  |
|  |  | 333.15 | 0.1-60 |  | 0.0199 | 0.0292 | 0.0199 |  |
|  |  | 343.15 | 0.1-60 |  | 0.0170 | 0.0265 | 0.0170 |  |
|  |  | 353.15 | 0.1-60.01 |  | 0.0105 | 0.0220 | 0.0104 |  |
|  |  | 363.15 | 0.1-60 |  | 0.0060 | 0.0153 | -0.0006 |  |
|  |  | 373.15 | 0.1-60.01 |  | 0.0116 | 0.0267 | -0.0102 |  |
|  |  | 393.15 | 0.1-60 |  | 0.0319 | 0.0540 | -0.0319 |  |
|  |  | 413.15 | 0.1-60 |  | 0.0510 | 0.0779 | -0.0510 |  |
|  |  | 433.15 | 0.1-60 |  | 0.0559 | 0.0962 | -0.0559 |  |
|  |  | 453.15 | 0.1-60 |  | 0.0409 | 0.1058 | -0.0409 |  |
|  |  | 473.15 | 0.1-60 |  | 0.0335 | 0.0885 | 0.0041 |  |
| [C_4_mim][NTf_2_] | REF 56 | 298.15 | 0.1-59.1 | for  T = 318.14 K  and  p = 46.8 MPa  0.09 % | 0.0119 | 0.0409 | 0.0059 | Isothermal Compressibility from REF 15 |
|  |  | 303.14 | 0.1-59.1 |  | 0.0268 | 0.0548 | 0.0267 |  |
|  |  | 308.09 | 0.1-59.1 |  | 0.0421 | 0.0652 | 0.0416 |  |
|  |  | 313.15 | 0.1-59.1 |  | 0.0592 | 0.0863 | 0.0592 |  |
|  |  | 318.14 | 0.1-59.1 |  | 0.0743 | 0.0947 | 0.0743 |  |
|  |  | 323.14 | 0.1-59.1 |  | 0.0386 | 0.0570 | 0.0369 |  |
|  |  | 328.2 | 0.1-59.1 |  | 0.0539 | 0.0716 | 0.0539 |  |
| [C_4_mim][NTf_2_] | REF 26 | 293.49 | 0.1-40 | for  T = 373.32 K  and  p = 40 MPa  0.09 % | 0.0291 | 0.0608 | 0.0290 | Isothermal Compressibility from REF 15 |
|  |  | 322.31 | 0.1-40 |  | 0.0181 | 0.0368 | 0.0181 |  |
|  |  | 352.33 | 0.1-40 |  | 0.0178 | 0.0375 | 0.0177 |  |
|  |  | 373.32 | 0.1-40 |  | 0.0215 | 0.0453 | 0.0200 |  |
|  |  | 391.28 | 0.1-40 |  | 0.0313 | 0.0685 | 0.0309 |  |
|  |  | 414.92 | 0.1-40 |  | 0.0548 | 0.1208 | 0.0548 |  |
| [C_4_mim][NTf_2_] | REF 81 | 273.15 | 0.1 | for  T = 288.15 K  and  p = 199.9 MPa  0.29 % | 0.0023 | 0.0023 | 0.0023 | Isothermal Compressibility from REF 15 |
|  |  | 278.15 | 0.1 |  | 0.0012 | 0.0012 | 0.0012 |  |
|  |  | 283.15 | 0.1 |  | 0.0009 | 0.0009 | -0.0009 |  |
|  |  | 288.15 | 0.1 |  | 0.0015 | 0.0015 | -0.0015 |  |
|  |  | 293.15 | 0.1 |  | 0.0017 | 0.0017 | -0.0017 |  |
|  |  | 293.15 | 0.1 |  | 0.0010 | 0.0010 | -0.0010 |  |
|  |  | 298.15 | 0.1 |  | 0.0016 | 0.0016 | -0.0016 |  |
|  |  | 298.15 | 0.1 |  | 0.0012 | 0.0012 | 0.0012 |  |
|  |  | 303.15 | 0.1 |  | 0.0012 | 0.0012 | -0.0012 |  |
|  |  | 313.15 | 0.1 |  | 0.0003 | 0.0003 | -0.0003 |  |
|  |  | 323.15 | 0.1 |  | 0.0011 | 0.0011 | 0.0011 |  |
|  |  | 323.15 | 0.1 |  | 0.0025 | 0.0025 | 0.0025 |  |
|  |  | 333.15 | 0.1 |  | 0.0009 | 0.0009 | 0.0009 |  |
|  |  | 343.15 | 0.1 |  | 0.0004 | 0.0004 | 0.0004 |  |
|  |  | 353.15 | 0.1 |  | 0.0003 | 0.0003 | -0.0003 |  |
|  |  | 363.15 | 0.1 |  | 0.0012 | 0.0012 | -0.0012 |  |
|  |  | 288.15 | 10.32-199.9 |  | 0.0973 | 0.2922 | 0.0949 |  |
|  |  | 288.16 | 99.6 |  | 0.1103 | 0.1103 | 0.1103 |  |
|  |  | 298.15 | 9.76-249.6 |  | 0.0726 | 0.1598 | 0.0592 |  |
|  |  | 298.16 | 20.12-225.5 |  | 0.0500 | 0.1333 | 0.0355 |  |
|  |  | 323.15 | 15.15-251.5 |  | 0.0398 | 0.1184 | 0.0354 |  |
|  |  | 323.16 | 10.25-201 |  | 0.0331 | 0.0774 | 0.0331 |  |
|  |  | 348.15 | 10.05-250.7 |  | 0.0556 | 0.2365 | -0.0470 |  |
| [C_4_mim][NTf_2_] | REF 55 | 298.15 | 0.1-50 | for  T = 298.15 K  and  p = 50 MPa  0.12 % | 0.0578 | 0.1157 | 0.0578 | Isothermal Compressibility from REF 15 |
|  |  | 318.15 | 0.1-50 |  | 0.0151 | 0.0380 | 0.0137 |  |
|  |  | 348.15 | 0.1-50 |  | 0.0322 | 0.0565 | 0.0322 |  |
| [C_4_mim][NTf_2_] | REF 50 | 273.15 | 0.1 | for  T = 348.15 K  and  p = 298.9 MPa  2.68 % | 0.0020 | 0.0020 | 0.0020 | Isothermal Compressibility from REF 15 |
|  |  | 278.15 | 0.1 |  | 0.0012 | 0.0012 | 0.0012 |  |
|  |  | 283.15 | 0.1 |  | 0.0008 | 0.0008 | -0.0008 |  |
|  |  | 288.15 | 0.1 |  | 0.0010 | 0.0010 | -0.0010 |  |
|  |  | 293.15 | 0.1 |  | 0.0011 | 0.0011 | -0.0011 |  |
|  |  | 298.15 | 0.1 |  | 0.0009 | 0.0009 | -0.0009 |  |
|  |  | 303.15 | 0.1 |  | 0.0004 | 0.0004 | -0.0004 |  |
|  |  | 313.15 | 0.1 |  | 0.0006 | 0.0006 | 0.0006 |  |
|  |  | 323.15 | 0.1 |  | 0.0020 | 0.0020 | 0.0020 |  |
|  |  | 333.15 | 0.1 |  | 0.0016 | 0.0016 | 0.0016 |  |
|  |  | 343.15 | 0.1 |  | 0.0009 | 0.0009 | 0.0009 |  |
|  |  | 348.15 | 0.1 |  | 0.0009 | 0.0009 | 0.0009 |  |
|  |  | 353.15 | 0.1 |  | 0.0003 | 0.0003 | -0.0003 |  |
|  |  | 363.15 | 0.1 |  | 0.0019 | 0.0019 | -0.0019 |  |
|  |  | 283.15 | 24.3-74.1 |  | 0.1147 | 0.2069 | 0.1147 |  |
|  |  | 298.15 | 24.3-150.2 |  | 0.1824 | 0.3876 | 0.1824 |  |
|  |  | 323.15 | 26.4-249.6 |  | 0.1171 | 0.3780 | -0.0540 |  |
|  |  | 348.15 | 0.8-298.9 |  | 0.9807 | 2.7524 | -0.9401 |  |
| [C_4_mim][NTf_2_] | REF 82 | 293.15 | 0.1 | for  T = 411.1 K  and  p = 200 MPa  0.37 % | 0.0071 | 0.0071 | 0.0071 | Isothermal Compressibility from REF 15 |
|  |  | 303.15 | 0.1 |  | 0.0075 | 0.0075 | -0.0075 |  |
|  |  | 313.15 | 0.1 |  | 0.0041 | 0.0041 | -0.0041 |  |
|  |  | 323.15 | 0.1 |  | 0.0037 | 0.0037 | 0.0037 |  |
|  |  | 333.15 | 0.1 |  | 0.0056 | 0.0056 | -0.0056 |  |
|  |  | 343.15 | 0.1 |  | 0.0037 | 0.0037 | 0.0037 |  |
|  |  | 353.15 | 0.1 |  | 0.0030 | 0.0030 | 0.0030 |  |
|  |  | 363.15 | 0.1 |  | 0.0067 | 0.0067 | 0.0067 |  |
|  |  | 373.15 | 0.1 |  | 0.0071 | 0.0071 | -0.0071 |  |
|  |  | 311.8 | 10-200 |  | 0.0882 | 0.1210 | 0.0882 |  |
|  |  | 331.3 | 10-200 |  | 0.0123 | 0.0313 | 0.0042 |  |
|  |  | 351.3 | 10-200 |  | 0.0375 | 0.1288 | -0.0353 |  |
|  |  | 371.3 | 10-200 |  | 0.0825 | 0.2145 | -0.0801 |  |
|  |  | 391.2 | 10-200 |  | 0.1295 | 0.3083 | -0.1260 |  |
|  |  | 411.1 | 10-200 |  | 0.1380 | 0.3684 | -0.1223 |  |
| [C_4_mim][OAc] | REF 7 | 298.1 | 0.1-25 | for  T = 298.15 K  and  p = 25 MPa  0.13 % | 0.0368 | 0.1250 | 0.0333 | Isothermal Compressibility from REF 7 |
|  |  | 302.9 | 0.1-25 |  | 0.0286 | 0.0955 | 0.0272 |  |
|  |  | 312.6 | 0.1-25 |  | 0.0179 | 0.0500 | 0.0178 |  |
|  |  | 322.45 | 0.1-25 |  | 0.0049 | 0.0112 | 0.0002 |  |
|  |  | 332.2 | 0.1-25 |  | 0.0026 | 0.0090 | 0.0012 |  |
|  |  | 343.26 | 0.1-25 |  | 0.0245 | 0.0366 | 0.0245 |  |
|  |  | 353.06 | 0.1-25 |  | 0.0039 | 0.0076 | -0.0009 |  |
| [C_4_mim][OAc] | REF 82 | 293.15 | 0.1-200 | for  T = 371.3 K  and  p = 200 MPa  1.66 % | 0.0148 | 0.0148 | -0.0148 | Isothermal Compressibility from REF 7 |
|  |  | 303.15 | 0.1-200 |  | 0.0128 | 0.0128 | 0.0128 |  |
|  |  | 313.15 | 0.1-200 |  | 0.0074 | 0.0074 | 0.0074 |  |
|  |  | 323.15 | 0.1-200 |  | 0.0068 | 0.0068 | 0.0068 |  |
|  |  | 333.15 | 0.1-200 |  | 0.0016 | 0.0016 | 0.0016 |  |
|  |  | 343.15 | 0.1-200 |  | 0.0084 | 0.0084 | -0.0084 |  |
|  |  | 353.15 | 0.1-200 |  | 0.0134 | 0.0134 | -0.0134 |  |
|  |  | 363.15 | 0.1-200 |  | 0.0035 | 0.0035 | -0.0035 |  |
|  |  | 373.15 | 0.1-200 |  | 0.0116 | 0.0116 | 0.0116 |  |
|  |  | 311.4 | 0.1-200 |  | 0.3977 | 0.8520 | 0.3977 |  |
|  |  | 331.1 | 0.1-200 |  | 0.1974 | 0.5227 | -0.1858 |  |
|  |  | 351.3 | 0.1-200 |  | 0.4954 | 1.2830 | -0.4753 |  |
|  |  | 371.3 | 0.1-200 |  | 0.6391 | 1.6915 | -0.5521 |  |
| [C_4_mim][OTf] | REF 24 | 293.15 | 0.1-10 | for  T = 393.15 K  and  p = 10 MPa  0.07 % | 0.0199 | 0.0413 | -0.0199 | Isothermal Compressibility calculated from REF 24 |
|  |  | 303.15 | 0.1-10 |  | 0.0081 | 0.0231 | -0.0050 |  |
|  |  | 313.15 | 0.1-10 |  | 0.0030 | 0.0058 | 0.0029 |  |
|  |  | 323.15 | 0.1-10 |  | 0.0071 | 0.0159 | 0.0035 |  |
|  |  | 333.15 | 0.1-10 |  | 0.0068 | 0.0135 | -0.0068 |  |
|  |  | 343.15 | 0.1-10 |  | 0.0181 | 0.0213 | -0.0181 |  |
|  |  | 353.15 | 0.1-10 |  | 0.0344 | 0.0491 | 0.0344 |  |
|  |  | 363.15 | 0.1-10 |  | 0.0081 | 0.0148 | -0.0039 |  |
|  |  | 373.15 | 0.1-10 |  | 0.0153 | 0.0250 | -0.0081 |  |
|  |  | 383.15 | 0.1-10 |  | 0.0096 | 0.0320 | 0.0038 |  |
|  |  | 393.15 | 0.1-10 |  | 0.0296 | 0.0679 | 0.0296 |  |
| [C_4_mim][OTf] | REF 14 | 290.497 | 40.403 | for  T = 291.26 K  and  p = 1.5921 MPa  0.30 % | 0.0641 | 0.0641 | 0.0641 | Density (p = 0.1 MPa), Isothermal Compressibility calculated from REF 24 |
|  |  | 290.513 | 20.585 |  | 0.1954 | 0.1954 | 0.1954 |  |
|  |  | 290.527 | 10.565 |  | 0.2121 | 0.2121 | 0.2121 |  |
|  |  | 290.692 | 30.827 |  | 0.1184 | 0.1184 | 0.1184 |  |
|  |  | 291.26 | 1.5921 |  | 0.2974 | 0.2974 | 0.2974 |  |
|  |  | 298.149 | 9.7577 |  | 0.2061 | 0.2061 | 0.2061 |  |
|  |  | 298.151 | 19.969 |  | 0.1249 | 0.1249 | 0.1249 |  |
|  |  | 298.153 | 20.141-50.367 |  | 0.0672 | 0.1319 | 0.0647 |  |
|  |  | 298.154 | 30.285 |  | 0.1194 | 0.1194 | 0.1194 |  |
|  |  | 298.156 | 30.053-40.41 |  | 0.0791 | 0.1104 | 0.0791 |  |
|  |  | 298.157 | 19.936 |  | 0.1233 | 0.1233 | 0.1233 |  |
|  |  | 298.158 | 9.707 |  | 0.2033 | 0.2033 | 0.2033 |  |
|  |  | 298.16 | 40.303 |  | 0.0440 | 0.0440 | 0.0440 |  |
|  |  | 298.166 | 10.0812 |  | 0.2182 | 0.2182 | 0.2182 |  |
|  |  | 298.167 | 50.464 |  | 0.0004 | 0.0004 | 0.0004 |  |
|  |  | 298.171 | 10.432 |  | 0.1855 | 0.1855 | 0.1855 |  |
|  |  | 299.03 | 2.4808 |  | 0.2249 | 0.2249 | 0.2249 |  |
|  |  | 305.413 | 59.674 |  | 0.0496 | 0.0496 | -0.0496 |  |
|  |  | 305.45 | 10.034 |  | 0.1590 | 0.1590 | 0.1590 |  |
|  |  | 305.451 | 19.292 |  | 0.1218 | 0.1218 | 0.1218 |  |
|  |  | 305.901 | 39.945 |  | 0.0597 | 0.0597 | 0.0597 |  |
|  |  | 305.999 | 29.921-50.404 |  | 0.0383 | 0.0727 | 0.0344 |  |
|  |  | 306.053 | 19.559 |  | 0.1454 | 0.1454 | 0.1454 |  |
|  |  | 307.165 | 1.752 |  | 0.1851 | 0.1851 | 0.1851 |  |
|  |  | 313.409 | 59.599 |  | 0.0424 | 0.0424 | -0.0424 |  |
|  |  | 313.929 | 49.845 |  | 0.0174 | 0.0174 | 0.0174 |  |
|  |  | 314.206 | 9.8391 |  | 0.1306 | 0.1306 | 0.1306 |  |
|  |  | 314.211 | 20.109-39.744 |  | 0.0531 | 0.0874 | 0.0531 |  |
|  |  | 314.323 | 29.477 |  | 0.0941 | 0.0941 | 0.0941 |  |
|  |  | 314.845 | 0.831 |  | 0.1538 | 0.1538 | 0.1538 |  |
|  |  | 321.663 | 59.111 |  | 0.0122 | 0.0122 | -0.0122 |  |
|  |  | 321.817 | 29.288 |  | 0.0680 | 0.0680 | 0.0680 |  |
|  |  | 323.069 | 39.773 |  | 0.0599 | 0.0599 | 0.0599 |  |
|  |  | 323.164 | 50.452 |  | 0.0117 | 0.0117 | -0.0117 |  |
|  |  | 323.182 | 10.082 |  | 0.1055 | 0.1055 | 0.1055 |  |
|  |  | 323.198 | 20.072 |  | 0.0856 | 0.0856 | 0.0856 |  |
|  |  | 323.398 | 1.5818 |  | 0.1622 | 0.1622 | 0.1622 |  |
|  |  | 330.391 | 28.233 |  | 0.0697 | 0.0697 | 0.0697 |  |
|  |  | 330.498 | 39.24 |  | 0.0462 | 0.0462 | 0.0462 |  |
|  |  | 331.304 | 0.2411 |  | 0.1230 | 0.1230 | 0.1230 |  |
|  |  | 331.38 | 10.182 |  | 0.1316 | 0.1316 | 0.1316 |  |
|  |  | 331.381 | 19.05 |  | 0.0762 | 0.0762 | 0.0762 |  |
|  |  | 331.385 | 59.986 |  | 0.0290 | 0.0290 | -0.0290 |  |
|  |  | 331.781 | 49.813 |  | 0.0418 | 0.0418 | 0.0418 |  |
|  |  | 339.842 | 49.769 |  | 0.0425 | 0.0425 | 0.0425 |  |
|  |  | 340.706 | 39.738 |  | 0.0669 | 0.0669 | 0.0669 |  |
|  |  | 340.733 | 10.118 |  | 0.1077 | 0.1077 | 0.1077 |  |
|  |  | 340.747 | 20.227 |  | 0.1222 | 0.1222 | 0.1222 |  |
|  |  | 340.934 | 60.046 |  | 0.0350 | 0.0350 | 0.0350 |  |
|  |  | 341.386 | 30.102 |  | 0.0769 | 0.0769 | 0.0769 |  |
|  |  | 341.681 | 1.3302 |  | 0.1096 | 0.1096 | 0.1096 |  |
|  |  | 347.879 | 58.421 |  | 0.0430 | 0.0430 | 0.0430 |  |
|  |  | 350.01 | 30.185 |  | 0.1397 | 0.1397 | 0.1397 |  |
|  |  | 350.144 | 9.9577 |  | 0.1176 | 0.1176 | 0.1176 |  |
|  |  | 350.152 | 19.909 |  | 0.1171 | 0.1171 | 0.1171 |  |
|  |  | 350.311 | 39.671 |  | 0.0885 | 0.0885 | 0.0885 |  |
|  |  | 350.378 | 50.208 |  | 0.0773 | 0.0773 | 0.0773 |  |
| [C_4_mim][SCN] | REF 6 | 298.15 | 0.1-10 | for  T = 308.15 K  and  p = 0.1 MPa  0.02 % | 0.0049 | 0.0078 | -0.0049 | Isothermal Compressibility from REF 6 |
|  |  | 308.15 | 0.1-10 |  | 0.0103 | 0.0170 | 0.0103 |  |
|  |  | 318.15 | 0.1-10 |  | 0.0106 | 0.0119 | -0.0106 |  |
|  |  | 328.15 | 0.1-10 |  | 0.0026 | 0.0058 | -0.0026 |  |
|  |  | 338.15 | 0.1-10 |  | 0.0015 | 0.0031 | 0.0011 |  |
| [C_4_mpyrro][(C_2_F_5_)_3_PF_3_] | REF 74 | 278.15 | 0.1-120 | for  T = 278.15 K  and  p = 120 MPa  0.14 % | 0.0469 | 0.1353 | -0.0395 | Isothermal Compressibility from REF 74 |
|  |  | 298.15 | 0.1-120 |  | 0.0464 | 0.1222 | -0.0263 |  |
|  |  | 313.15 | 0.1-120 |  | 0.0281 | 0.0911 | -0.0189 |  |
|  |  | 333.15 | 0.1-120 |  | 0.0177 | 0.0588 | -0.0140 |  |
|  |  | 348.15 | 0.1-120 |  | 0.0232 | 0.0535 | -0.0232 |  |
|  |  | 373.15 | 0.1-120 |  | 0.0129 | 0.0219 | -0.0001 |  |
|  |  | 398.15 | 0.1-120 |  | 0.0347 | 0.0667 | 0.0135 |  |
| [C_4_mpyrro][(C_2_F_5_)_3_PF_3_] | REF 69 | 293.19 | 0.1 | for  T = 293.17 K  and  p = 15 MPa  0.11 % | 0.0034 | 0.0034 | 0.0034 | Isothermal Compressibility from REF 74 |
|  |  | 303.18 | 0.1 |  | 0.0039 | 0.0039 | -0.0039 |  |
|  |  | 313.2 | 0.1 |  | 0.0055 | 0.0055 | -0.0055 |  |
|  |  | 323.14 | 0.1 |  | 0.0064 | 0.0064 | 0.0064 |  |
|  |  | 333.17 | 0.1 |  | 0.0009 | 0.0009 | 0.0009 |  |
|  |  | 343.16 | 0.1 |  | 0.0002 | 0.0002 | -0.0002 |  |
|  |  | 353.17 | 0.1 |  | 0.0011 | 0.0011 | -0.0011 |  |
|  |  | 293.19 | 0.5 |  | 0.0056 | 0.0056 | 0.0056 |  |
|  |  | 303.2 | 0.5 |  | 0.0022 | 0.0022 | -0.0022 |  |
|  |  | 313.2 | 0.5 |  | 0.0079 | 0.0079 | -0.0079 |  |
|  |  | 323.14 | 0.5 |  | 0.0048 | 0.0048 | 0.0048 |  |
|  |  | 333.17 | 0.5 |  | 0.0003 | 0.0003 | 0.0003 |  |
|  |  | 343.17 | 0.5 |  | 0.0070 | 0.0070 | -0.0070 |  |
|  |  | 353.17 | 0.5 |  | 0.0127 | 0.0127 | -0.0127 |  |
|  |  | 293.19 | 1 |  | 0.0379 | 0.0379 | 0.0379 |  |
|  |  | 303.19 | 1 |  | 0.0257 | 0.0257 | 0.0257 |  |
|  |  | 313.2 | 1 |  | 0.0207 | 0.0207 | 0.0207 |  |
|  |  | 323.15 | 1 |  | 0.0276 | 0.0276 | 0.0276 |  |
|  |  | 333.17 | 1 |  | 0.0251 | 0.0251 | 0.0251 |  |
|  |  | 343.18 | 1 |  | 0.0186 | 0.0186 | 0.0186 |  |
|  |  | 353.18 | 1 |  | 0.0078 | 0.0078 | 0.0078 |  |
|  |  | 293.18 | 2.5 |  | 0.0541 | 0.0541 | 0.0541 |  |
|  |  | 303.26 | 2.5 |  | 0.0521 | 0.0521 | 0.0521 |  |
|  |  | 313.21 | 2.5 |  | 0.0418 | 0.0418 | 0.0418 |  |
|  |  | 323.14 | 2.5 |  | 0.0536 | 0.0536 | 0.0536 |  |
|  |  | 333.18 | 2.5 |  | 0.0470 | 0.0470 | 0.0470 |  |
|  |  | 343.19 | 2.5 |  | 0.0442 | 0.0442 | 0.0442 |  |
|  |  | 353.18 | 2.5 |  | 0.0381 | 0.0381 | 0.0381 |  |
|  |  | 293.17 | 5 |  | 0.0707 | 0.0707 | 0.0707 |  |
|  |  | 303.26 | 5 |  | 0.0668 | 0.0668 | 0.0668 |  |
|  |  | 313.2 | 5 |  | 0.0562 | 0.0562 | 0.0562 |  |
|  |  | 323.15 | 5 |  | 0.0656 | 0.0656 | 0.0656 |  |
|  |  | 333.17 | 5 |  | 0.0597 | 0.0597 | 0.0597 |  |
|  |  | 343.2 | 5 |  | 0.0552 | 0.0552 | 0.0552 |  |
|  |  | 353.17 | 5 |  | 0.0502 | 0.0502 | 0.0502 |  |
|  |  | 293.17 | 10 |  | 0.1038 | 0.1038 | 0.1038 |  |
|  |  | 303.25 | 10 |  | 0.0977 | 0.0977 | 0.0977 |  |
|  |  | 313.19 | 10 |  | 0.0908 | 0.0908 | 0.0908 |  |
|  |  | 323.14 | 10 |  | 0.1043 | 0.1043 | 0.1043 |  |
|  |  | 333.18 | 10 |  | 0.1017 | 0.1017 | 0.1017 |  |
|  |  | 343.2 | 10 |  | 0.0965 | 0.0965 | 0.0965 |  |
|  |  | 353.19 | 10 |  | 0.0893 | 0.0893 | 0.0893 |  |
|  |  | 293.17 | 15 |  | 0.1056 | 0.1056 | 0.1056 |  |
|  |  | 303.26 | 15 |  | 0.0954 | 0.0954 | 0.0954 |  |
|  |  | 313.21 | 15 |  | 0.0907 | 0.0907 | 0.0907 |  |
|  |  | 323.14 | 15 |  | 0.1025 | 0.1025 | 0.1025 |  |
|  |  | 333.18 | 15 |  | 0.1036 | 0.1036 | 0.1036 |  |
|  |  | 343.2 | 15 |  | 0.0962 | 0.0962 | 0.0962 |  |
|  |  | 353.18 | 15 |  | 0.0942 | 0.0942 | 0.0942 |  |
|  |  | 293.17 | 20 |  | 0.1013 | 0.1013 | 0.1013 |  |
|  |  | 303.25 | 20 |  | 0.0940 | 0.0940 | 0.0940 |  |
|  |  | 313.2 | 20 |  | 0.0918 | 0.0918 | 0.0918 |  |
|  |  | 323.14 | 20 |  | 0.0994 | 0.0994 | 0.0994 |  |
|  |  | 333.18 | 20 |  | 0.0974 | 0.0974 | 0.0974 |  |
|  |  | 343.2 | 20 |  | 0.0935 | 0.0935 | 0.0935 |  |
|  |  | 353.19 | 20 |  | 0.0946 | 0.0946 | 0.0946 |  |
|  |  | 293.17 | 25 |  | 0.1037 | 0.1037 | 0.1037 |  |
|  |  | 303.25 | 25 |  | 0.0983 | 0.0983 | 0.0983 |  |
|  |  | 313.21 | 25 |  | 0.0913 | 0.0913 | 0.0913 |  |
|  |  | 323.14 | 25 |  | 0.1017 | 0.1017 | 0.1017 |  |
|  |  | 333.18 | 25 |  | 0.1024 | 0.1024 | 0.1024 |  |
|  |  | 343.21 | 25 |  | 0.1009 | 0.1009 | 0.1009 |  |
|  |  | 353.19 | 25 |  | 0.0995 | 0.0995 | 0.0995 |  |
| [C_4_mpyrro][B(CN)_4_] | REF 74 | 298.15 | 0.1-15 | for  T = 313.15 K  and  p = 60 MPa  0.13 % | 0.0094 | 0.0254 | -0.0072 | Isothermal Compressibility from REF 74 |
|  |  | 313.15 | 0.1-60 |  | 0.0641 | 0.1265 | -0.0641 |  |
|  |  | 333.15 | 0.1-60 |  | 0.0327 | 0.0844 | -0.0324 |  |
|  |  | 348.15 | 0.1-60 |  | 0.0220 | 0.0578 | -0.0220 |  |
|  |  | 373.15 | 0.1-60 |  | 0.0108 | 0.0318 | -0.0053 |  |
|  |  | 398.15 | 0.1-60 |  | 0.0233 | 0.0715 | -0.0101 |  |
| [C_4_mpyrro][NTf_2_] | REF 45 | 293.15 | 0.1-35 | for  T = 393.15 K  and  p = 35 MPa  0.03 % | 0.0062 | 0.0232 | -0.0030 | Isothermal Compressibility from REF 45 |
|  |  | 303.15 | 0.1-35 |  | 0.0056 | 0.0157 | -0.0052 |  |
|  |  | 313.15 | 0.1-35 |  | 0.0031 | 0.0074 | -0.0015 |  |
|  |  | 323.15 | 0.1-35 |  | 0.0068 | 0.0100 | 0.0068 |  |
|  |  | 333.15 | 0.1-35 |  | 0.0094 | 0.0139 | 0.0094 |  |
|  |  | 353.15 | 0.1-35 |  | 0.0057 | 0.0175 | 0.0018 |  |
|  |  | 393.15 | 0.1-35 |  | 0.0094 | 0.0345 | 0.0039 |  |
| [C_4_mpyrro][NTf_2_] | REF 53 | 293.51 | 1 | for  T = 293.5 K  and  p = 40 MPa  1.00 % | 0.7683 | 0.7683 | 0.7683 | Density (p = 0.1 MPa), Isothermal Compressibility from REF 45 |
|  |  | 322.33 | 1 |  | 0.5444 | 0.5444 | 0.5444 |  |
|  |  | 352.35 | 1 |  | 0.3624 | 0.3624 | 0.3624 |  |
|  |  | 373.32 | 1 |  | 0.2778 | 0.2778 | 0.2778 |  |
|  |  | 391.26 | 1 |  | 0.2172 | 0.2172 | 0.2172 |  |
|  |  | 414.94 | 1 |  | 0.1867 | 0.1867 | 0.1867 |  |
|  |  | 293.51 | 5 |  | 0.7998 | 0.7998 | 0.7998 |  |
|  |  | 322.32 | 5 |  | 0.5841 | 0.5841 | 0.5841 |  |
|  |  | 352.33 | 5 |  | 0.4048 | 0.4048 | 0.4048 |  |
|  |  | 373.31 | 5 |  | 0.3219 | 0.3219 | 0.3219 |  |
|  |  | 391.3 | 5 |  | 0.2653 | 0.2653 | 0.2653 |  |
|  |  | 414.93 | 5 |  | 0.2427 | 0.2427 | 0.2427 |  |
|  |  | 293.5 | 10 |  | 0.8340 | 0.8340 | 0.8340 |  |
|  |  | 322.31 | 10 |  | 0.6233 | 0.6233 | 0.6233 |  |
|  |  | 352.32 | 10 |  | 0.4580 | 0.4580 | 0.4580 |  |
|  |  | 373.33 | 10 |  | 0.3715 | 0.3715 | 0.3715 |  |
|  |  | 391.29 | 10 |  | 0.3273 | 0.3273 | 0.3273 |  |
|  |  | 414.93 | 10 |  | 0.2973 | 0.2973 | 0.2973 |  |
|  |  | 293.5 | 20 |  | 0.8976 | 0.8976 | 0.8976 |  |
|  |  | 322.31 | 20 |  | 0.7008 | 0.7008 | 0.7008 |  |
|  |  | 352.31 | 20 |  | 0.5392 | 0.5392 | 0.5392 |  |
|  |  | 373.34 | 20 |  | 0.4752 | 0.4752 | 0.4752 |  |
|  |  | 391.32 | 20 |  | 0.4334 | 0.4334 | 0.4334 |  |
|  |  | 414.93 | 20 |  | 0.4117 | 0.4117 | 0.4117 |  |
|  |  | 293.5 | 30 |  | 0.9465 | 0.9465 | 0.9465 |  |
|  |  | 322.32 | 30 |  | 0.7593 | 0.7593 | 0.7593 |  |
|  |  | 352.33 | 30 |  | 0.6177 | 0.6177 | 0.6177 |  |
|  |  | 373.31 | 30 |  | 0.5556 | 0.5556 | 0.5556 |  |
|  |  | 391.29 | 30 |  | 0.5221 | 0.5221 | 0.5221 |  |
|  |  | 414.93 | 30 |  | 0.5241 | 0.5241 | 0.5241 |  |
|  |  | 293.5 | 40 |  | 0.9897 | 0.9897 | 0.9897 |  |
|  |  | 322.31 | 40 |  | 0.8104 | 0.8104 | 0.8104 |  |
|  |  | 352.31 | 40 |  | 0.6864 | 0.6864 | 0.6864 |  |
|  |  | 373.34 | 40 |  | 0.6321 | 0.6321 | 0.6321 |  |
|  |  | 391.32 | 40 |  | 0.6048 | 0.6048 | 0.6048 |  |
|  |  | 414.93 | 40 |  | 0.6085 | 0.6085 | 0.6085 |  |
| [C_4_mpyrro][NTf_2_] | REF 53 | 293.51 | 1 | for  T = 293.5 K  and  p = 40 MPa  0.04 % | 0.7683 | 0.7683 | 0.7683 | Density (p = 0.1 MPa), Isothermal Compressibility from REF 53 |
|  |  | 322.33 | 1 |  | 0.5444 | 0.5444 | 0.5444 |  |
|  |  | 352.35 | 1 |  | 0.3624 | 0.3624 | 0.3624 |  |
|  |  | 373.32 | 1 |  | 0.2778 | 0.2778 | 0.2778 |  |
|  |  | 391.26 | 1 |  | 0.2172 | 0.2172 | 0.2172 |  |
|  |  | 414.94 | 1 |  | 0.1867 | 0.1867 | 0.1867 |  |
|  |  | 293.51 | 5 |  | 0.7998 | 0.7998 | 0.7998 |  |
|  |  | 322.32 | 5 |  | 0.5841 | 0.5841 | 0.5841 |  |
|  |  | 352.33 | 5 |  | 0.4048 | 0.4048 | 0.4048 |  |
|  |  | 373.31 | 5 |  | 0.3219 | 0.3219 | 0.3219 |  |
|  |  | 391.3 | 5 |  | 0.2653 | 0.2653 | 0.2653 |  |
|  |  | 414.93 | 5 |  | 0.2427 | 0.2427 | 0.2427 |  |
|  |  | 293.5 | 10 |  | 0.8340 | 0.8340 | 0.8340 |  |
|  |  | 322.31 | 10 |  | 0.6233 | 0.6233 | 0.6233 |  |
|  |  | 352.32 | 10 |  | 0.4580 | 0.4580 | 0.4580 |  |
|  |  | 373.33 | 10 |  | 0.3715 | 0.3715 | 0.3715 |  |
|  |  | 391.29 | 10 |  | 0.3273 | 0.3273 | 0.3273 |  |
|  |  | 414.93 | 10 |  | 0.2973 | 0.2973 | 0.2973 |  |
|  |  | 293.5 | 20 |  | 0.8976 | 0.8976 | 0.8976 |  |
|  |  | 322.31 | 20 |  | 0.7008 | 0.7008 | 0.7008 |  |
|  |  | 352.31 | 20 |  | 0.5392 | 0.5392 | 0.5392 |  |
|  |  | 373.34 | 20 |  | 0.4752 | 0.4752 | 0.4752 |  |
|  |  | 391.32 | 20 |  | 0.4334 | 0.4334 | 0.4334 |  |
|  |  | 414.93 | 20 |  | 0.4117 | 0.4117 | 0.4117 |  |
|  |  | 293.5 | 30 |  | 0.9465 | 0.9465 | 0.9465 |  |
|  |  | 322.32 | 30 |  | 0.7593 | 0.7593 | 0.7593 |  |
|  |  | 352.33 | 30 |  | 0.6177 | 0.6177 | 0.6177 |  |
|  |  | 373.31 | 30 |  | 0.5556 | 0.5556 | 0.5556 |  |
|  |  | 391.29 | 30 |  | 0.5221 | 0.5221 | 0.5221 |  |
|  |  | 414.93 | 30 |  | 0.5241 | 0.5241 | 0.5241 |  |
|  |  | 293.5 | 40 |  | 0.9897 | 0.9897 | 0.9897 |  |
|  |  | 322.31 | 40 |  | 0.8104 | 0.8104 | 0.8104 |  |
|  |  | 352.31 | 40 |  | 0.6864 | 0.6864 | 0.6864 |  |
|  |  | 373.34 | 40 |  | 0.6321 | 0.6321 | 0.6321 |  |
|  |  | 391.32 | 40 |  | 0.6048 | 0.6048 | 0.6048 |  |
|  |  | 414.93 | 40 |  | 0.6085 | 0.6085 | 0.6085 |  |
| [C_4_mpyrro][NTf_2_] | REF 57 | 273.15 | 0.82-80.52 | for  T = 348.15 K  and  p = 102.92 MPa  0.33 % | 0.1328 | 0.2393 | -0.1328 | Isothermal Compressibility from REF 45 |
|  |  | 298.15 | 0.86-100.23 |  | 0.0356 | 0.0920 | -0.0356 |  |
|  |  | 283.15 | 10.14-100.39 |  | 0.1202 | 0.2187 | -0.1202 |  |
|  |  | 323.15 | 10.52-100.4 |  | 0.0855 | 0.1189 | 0.0855 |  |
|  |  | 348.15 | 10.85-102.92 |  | 0.1826 | 0.3269 | 0.1826 |  |
| [C_4_mpyrro][NTf_2_] | REF 58 | 278.15 | 0.1-120 | for  T = 398.15 K  and  p = 120 MPa  0.33 % | 0.0413 | 0.0787 | -0.0413 | Isothermal Compressibility from REF 45 |
|  |  | 298.15 | 0.1-120 |  | 0.0929 | 0.1345 | 0.0929 |  |
|  |  | 313.15 | 0.1-120 |  | 0.0786 | 0.1240 | 0.0786 |  |
|  |  | 333.15 | 0.1-120 |  | 0.0810 | 0.1410 | 0.0810 |  |
|  |  | 348.15 | 0.1-120 |  | 0.0732 | 0.1530 | 0.0697 |  |
|  |  | 373.15 | 0.1-120 |  | 0.0865 | 0.1832 | 0.0536 |  |
|  |  | 398.15 | 0.1-120 |  | 0.1815 | 0.3261 | 0.1815 |  |
| [C_4_mpyrro][OTf] | REF 74 | 278.15 | 0.1-120 | for  T = 278.15 K  and  p = 120 MPa  0.15 % | 0.0583 | 0.1537 | -0.0537 | Isothermal Compressibility calculated from REF 74 |
|  |  | 298.15 | 0.1-120 |  | 0.0520 | 0.1330 | -0.0173 |  |
|  |  | 313.15 | 0.1-120 |  | 0.0378 | 0.1064 | -0.0224 |  |
|  |  | 333.15 | 0.1-120 |  | 0.0409 | 0.1229 | -0.0396 |  |
|  |  | 348.15 | 0.1-120 |  | 0.0516 | 0.1199 | -0.0516 |  |
|  |  | 373.15 | 0.1-120 |  | 0.0290 | 0.0760 | -0.0259 |  |
|  |  | 398.15 | 0.1-120 |  | 0.0170 | 0.0369 | -0.0150 |  |
| [C_4_py][BF_4_] | REF 19 | 298.2 | 0.1 | for  T = 323.2 K  and  p = 172.47 MPa  0.43 % | 0.0165 | 0.0165 | 0.0165 | Isothermal Compressibility from REF 75 |
|  |  | 303.2 | 0.1 |  | 0.0218 | 0.0218 | -0.0218 |  |
|  |  | 313.2 | 0.1 |  | 0.0015 | 0.0015 | -0.0015 |  |
|  |  | 323.2 | 0.1 |  | 0.0054 | 0.0054 | 0.0054 |  |
|  |  | 333.2 | 0.1 |  | 0.0069 | 0.0069 | 0.0069 |  |
|  |  | 343.2 | 0.1 |  | 0.0054 | 0.0054 | -0.0054 |  |
|  |  | 298.2 | 23.54-202.81 |  | 0.1458 | 0.3022 | 0.1180 |  |
|  |  | 323.2 | 71.46-204.18 |  | 0.2635 | 0.4249 | 0.2635 |  |
| [C_4_py][BF_4_] | REF 20 | 293.15 | 0.1-20 | for  T = 343.15 K  and  p = 20 MPa  0.03 % | 0.0192 | 0.0270 | -0.0192 | Isothermal Compressibility from REF 75 |
|  |  | 303.15 | 0.1-20 |  | 0.0091 | 0.0158 | -0.0072 |  |
|  |  | 313.15 | 0.1-20 |  | 0.0128 | 0.0213 | -0.0107 |  |
|  |  | 323.15 | 0.1-20 |  | 0.0198 | 0.0241 | -0.0198 |  |
|  |  | 333.15 | 0.1-20 |  | 0.0037 | 0.0048 | 0.0006 |  |
|  |  | 343.15 | 0.1-20 |  | 0.0207 | 0.0281 | 0.0207 |  |
|  |  | 353.15 | 0.1-20 |  | 0.0052 | 0.0083 | -0.0001 |  |
| [C_4_py][BF_4_] | REF 75 | 283.15 | 0.1-65 | for  T = 328.15 K  and  p = 60 MPa  0.07 % | 0.0196 | 0.0606 | 0.0185 | Isothermal Compressibility from REF 75 |
|  |  | 288.15 | 0.1-65 |  | 0.0256 | 0.0646 | 0.0255 |  |
|  |  | 293.15 | 0.1-65 |  | 0.0278 | 0.0612 | 0.0278 |  |
|  |  | 298.15 | 0.1-65 |  | 0.0207 | 0.0640 | 0.0207 |  |
|  |  | 303.15 | 0.1-65 |  | 0.0188 | 0.0595 | 0.0146 |  |
|  |  | 308.15 | 0.1-65 |  | 0.0295 | 0.0593 | 0.0295 |  |
|  |  | 313.15 | 0.1-65 |  | 0.0143 | 0.0499 | 0.0110 |  |
|  |  | 318.15 | 0.1-65 |  | 0.0290 | 0.0612 | 0.0282 |  |
|  |  | 323.15 | 0.1-65 |  | 0.0195 | 0.0588 | 0.0161 |  |
|  |  | 328.15 | 0.1-65 |  | 0.0358 | 0.0709 | 0.0358 |  |
|  |  | 333.15 | 0.1-65 |  | 0.0218 | 0.0557 | 0.0211 |  |
| [C_4_py][OTf] | REF 75 | 303.15 | 0.1-65 | for  T = 303.15 K  and  p = 65 MPa  0.88 % | 0.4334 | 0.8756 | 0.4333 | Isothermal Compressibility from REF 75 |
|  |  | 308.15 | 0.1-65 |  | 0.4205 | 0.8676 | 0.4202 |  |
|  |  | 313.15 | 0.1-65 |  | 0.4183 | 0.8443 | 0.4183 |  |
|  |  | 318.15 | 0.1-65 |  | 0.4181 | 0.8353 | 0.4181 |  |
|  |  | 323.15 | 0.1-65 |  | 0.3969 | 0.8185 | 0.3951 |  |
|  |  | 328.15 | 0.1-65 |  | 0.3954 | 0.7937 | 0.3954 |  |
|  |  | 333.15 | 0.1-65 |  | 0.3713 | 0.7609 | 0.3712 |  |
| [C_5_mim][NTf_2_] | REF 54 | 298.15 | 0.1-59.59 | for  T = 298.15 K  and  p = 59.59 MPa  0.61 % | 0.1941 | 0.6035 | 0.1933 | Isothermal Compressibility from REF 54 |
|  |  | 299.15 | 0.1-59.59 |  | 0.1791 | 0.5552 | 0.1791 |  |
|  |  | 300.15 | 0.1-59.59 |  | 0.1642 | 0.5043 | 0.1642 |  |
|  |  | 301.15 | 0.1-59.59 |  | 0.1508 | 0.4638 | 0.1497 |  |
|  |  | 302.15 | 0.1-59.59 |  | 0.1322 | 0.4136 | 0.1315 |  |
|  |  | 303.15 | 0.1-59.59 |  | 0.1218 | 0.3742 | 0.1218 |  |
|  |  | 308.15 | 0.1-59.59 |  | 0.0701 | 0.2080 | 0.0701 |  |
|  |  | 313.15 | 0.1-59.59 |  | 0.0357 | 0.0790 | 0.0357 |  |
|  |  | 318.15 | 0.1-59.59 |  | 0.0112 | 0.0264 | 0.0036 |  |
|  |  | 323.15 | 0.1-59.59 |  | 0.0374 | 0.1075 | -0.0374 |  |
|  |  | 325.15 | 0.1-59.59 |  | 0.0676 | 0.1613 | -0.0676 |  |
|  |  | 327.15 | 0.1-59.59 |  | 0.0729 | 0.1927 | -0.0729 |  |
|  |  | 329.15 | 0.1-59.59 |  | 0.0710 | 0.2066 | -0.0710 |  |
|  |  | 331.15 | 0.1-59.59 |  | 0.0707 | 0.2160 | -0.0689 |  |
|  |  | 333.15 | 0.1-59.59 |  | 0.0707 | 0.2231 | -0.0610 |  |
| [C_6_mim][(C_2_F_5_)_3_PF_3_] | REF 68 | 293.16 | 0.103-25 | for  T = 343.13 K  and  p = 25 MPa  0.20 % | 0.1308 | 0.1363 | 0.1308 | density, Isothermal Compressibility calculated using the GCM method proposed in REF 53 |
|  |  | 293.17 | 0.253-1 |  | 0.1275 | 0.1283 | 0.1275 |  |
|  |  | 293.18 | 2.5 |  | 0.1416 | 0.1416 | 0.1416 |  |
|  |  | 303.17 | 0.5 |  | 0.1488 | 0.1488 | 0.1488 |  |
|  |  | 303.18 | 0.103-20 |  | 0.1485 | 0.1544 | 0.1485 |  |
|  |  | 303.19 | 25 |  | 0.1616 | 0.1616 | 0.1616 |  |
|  |  | 313.17 | 0.103 |  | 0.1496 | 0.1496 | 0.1496 |  |
|  |  | 313.18 | 0.253-25 |  | 0.1622 | 0.1724 | 0.1622 |  |
|  |  | 313.19 | 0.5-20 |  | 0.1570 | 0.1667 | 0.1570 |  |
|  |  | 323.14 | 0.5 |  | 0.1715 | 0.1715 | 0.1715 |  |
|  |  | 323.15 | 1-2.5 |  | 0.1656 | 0.1663 | 0.1656 |  |
|  |  | 323.16 | 0.253 |  | 0.1732 | 0.1732 | 0.1732 |  |
|  |  | 323.17 | 0.103 |  | 0.1690 | 0.1690 | 0.1690 |  |
|  |  | 323.19 | 5-25 |  | 0.1826 | 0.1970 | 0.1826 |  |
|  |  | 333.18 | 25 |  | 0.1926 | 0.1926 | 0.1926 |  |
|  |  | 333.19 | 0.103-20 |  | 0.1686 | 0.1853 | 0.1686 |  |
|  |  | 343.13 | 20-25 |  | 0.1936 | 0.1991 | 0.1936 |  |
|  |  | 343.14 | 15 |  | 0.1791 | 0.1791 | 0.1791 |  |
|  |  | 343.15 | 10 |  | 0.1724 | 0.1724 | 0.1724 |  |
|  |  | 343.16 | 5 |  | 0.1672 | 0.1672 | 0.1672 |  |
|  |  | 343.18 | 0.253-2.5 |  | 0.1684 | 0.1721 | 0.1684 |  |
|  |  | 343.19 | 0.103 |  | 0.1687 | 0.1687 | 0.1687 |  |
| [C_6_mim][BF_4_] | REF 12 | 293.15 | 0.1 | for  T = 472.5 K  and  p = 80 MPa  0.71 % | 0.0009 | 0.0009 | 0.0009 | Isothermal Compressibility calculated from Tait equation from REF 13 |
|  |  | 313.15 | 0.1 |  | 0.0026 | 0.0026 | -0.0026 |  |
|  |  | 333.15 | 0.1 |  | 0.0027 | 0.0027 | 0.0027 |  |
|  |  | 353.15 | 0.1 |  | 0.0009 | 0.0009 | -0.0009 |  |
|  |  | 313.3 | 10-200 |  | 0.1783 | 0.4519 | -0.1683 |  |
|  |  | 332.5 | 10-200 |  | 0.1342 | 0.3480 | -0.0954 |  |
|  |  | 352.6 | 10-200 |  | 0.1148 | 0.3334 | -0.0385 |  |
|  |  | 372.7 | 10-200 |  | 0.1142 | 0.2011 | 0.0215 |  |
|  |  | 393 | 10-200 |  | 0.1372 | 0.2284 | 0.0806 |  |
|  |  | 413 | 10-200 |  | 0.1819 | 0.2885 | 0.1616 |  |
|  |  | 432.9 | 10-200 |  | 0.2576 | 0.4140 | 0.2547 |  |
|  |  | 452.6 | 10-200 |  | 0.3960 | 0.5654 | 0.3960 |  |
|  |  | 472.5 | 10-200 |  | 0.5230 | 0.7064 | 0.5230 |  |
| [C_6_mim][BF_4_] | REF 13 | 283.15 | 0.1-60 | for  T = 283.15 K  and  p = 60 MPa  0.06 % | 0.0128 | 0.0591 | -0.0118 | Isothermal Compressibility calculated from Tait equation from REF 13 |
|  |  | 288.15 | 0.1-60 |  | 0.0137 | 0.0521 | -0.0121 |  |
|  |  | 293.15 | 0.1-60 |  | 0.0120 | 0.0453 | -0.0117 |  |
|  |  | 298.15 | 0.1-60 |  | 0.0099 | 0.0413 | -0.0099 |  |
|  |  | 303.15 | 0.1-60 |  | 0.0081 | 0.0359 | -0.0077 |  |
|  |  | 308.15 | 0.1-60 |  | 0.0054 | 0.0280 | -0.0028 |  |
|  |  | 313.15 | 0.1-60 |  | 0.0036 | 0.0220 | -0.0026 |  |
|  |  | 318.15 | 0.1-60 |  | 0.0032 | 0.0119 | 0.0002 |  |
|  |  | 323.15 | 0.1-60 |  | 0.0095 | 0.0565 | 0.0091 |  |
| [C_6_mim][BF_4_] | REF 14 | 298.155 | 0.104 | for  T = 242.307 K  and  p = 19.417 MPa  0.44 % | 0.0602 | 0.0602 | -0.0602 | Density (p = 0.1 MPa) from REF 13, Isothermal Compressibility calculated from Tait equation from REF 13 |
|  |  | 299.113 | 0.7125 |  | 0.0160 | 0.0160 | -0.0160 |  |
|  |  | 230.793 | 1.0128 |  | 0.1543 | 0.1543 | -0.1543 |  |
|  |  | 333.472 | 1.0898 |  | 0.0189 | 0.0189 | 0.0189 |  |
|  |  | 315.842 | 1.2342 |  | 0.0065 | 0.0065 | -0.0065 |  |
|  |  | 349.189 | 1.3743 |  | 0.0001 | 0.0001 | -0.0001 |  |
|  |  | 256.483 | 1.7425 |  | 0.1332 | 0.1332 | -0.1332 |  |
|  |  | 243.645 | 1.9416 |  | 0.1476 | 0.1476 | -0.1476 |  |
|  |  | 269.899 | 2.1192 |  | 0.0104 | 0.0104 | 0.0104 |  |
|  |  | 284.269 | 2.1899 |  | 0.0200 | 0.0200 | -0.0200 |  |
|  |  | 219.916 | 2.5017 |  | 0.1718 | 0.1718 | -0.1718 |  |
|  |  | 357.125 | 9.5951 |  | 0.0259 | 0.0259 | 0.0259 |  |
|  |  | 341.606 | 9.7798 |  | 0.0255 | 0.0255 | 0.0255 |  |
|  |  | 323.554 | 9.9644 |  | 0.0101 | 0.0101 | -0.0101 |  |
|  |  | 306.824 | 10.007 |  | 0.0242 | 0.0242 | -0.0242 |  |
|  |  | 275.799 | 10.046 |  | 0.0034 | 0.0034 | -0.0034 |  |
|  |  | 249.02 | 10.085 |  | 0.1562 | 0.1562 | -0.1562 |  |
|  |  | 236.445 | 10.093 |  | 0.1605 | 0.1605 | -0.1605 |  |
|  |  | 262.309 | 10.14 |  | 0.1426 | 0.1426 | -0.1426 |  |
|  |  | 290.725 | 10.282 |  | 0.0380 | 0.0380 | -0.0380 |  |
|  |  | 225.139 | 11.244 |  | 0.1784 | 0.1784 | -0.1784 |  |
|  |  | 242.307 | 19.417 |  | 0.1684 | 0.1684 | -0.1684 |  |
|  |  | 269.118 | 19.855 |  | 0.1533 | 0.1533 | -0.1533 |  |
|  |  | 332.357 | 19.953 |  | 0.0037 | 0.0037 | -0.0037 |  |
|  |  | 298.522 | 20.112 |  | 0.0512 | 0.0512 | -0.0512 |  |
|  |  | 255.694 | 20.148 |  | 0.1663 | 0.1663 | -0.1663 |  |
|  |  | 315.252 | 20.171 |  | 0.0257 | 0.0257 | -0.0257 |  |
|  |  | 351.2 | 20.298 |  | 0.0593 | 0.0593 | 0.0593 |  |
|  |  | 283.499 | 20.904 |  | 0.0034 | 0.0034 | 0.0034 |  |
|  |  | 231.393 | 21.744 |  | 0.1845 | 0.1845 | -0.1845 |  |
|  |  | 357.115 | 26.381 |  | 0.0668 | 0.0668 | 0.0668 |  |
|  |  | 275.797 | 29.402 |  | 0.1595 | 0.1595 | -0.1595 |  |
|  |  | 323.548 | 29.837 |  | 0.0343 | 0.0343 | -0.0343 |  |
|  |  | 290.726 | 29.904 |  | 0.0318 | 0.0318 | -0.0318 |  |
|  |  | 341.626 | 29.941 |  | 0.0113 | 0.0113 | -0.0113 |  |
|  |  | 249.02 | 30.013 |  | 0.1774 | 0.1774 | -0.1774 |  |
|  |  | 262.309 | 30.102 |  | 0.1745 | 0.1745 | -0.1745 |  |
|  |  | 236.457 | 30.111 |  | 0.1921 | 0.1921 | -0.1921 |  |
|  |  | 290.664 | 30.442 |  | 0.0078 | 0.0078 | -0.0078 |  |
|  |  | 290.666 | 30.768 |  | 0.0640 | 0.0640 | -0.0640 |  |
|  |  | 306.83 | 30.854 |  | 0.0471 | 0.0471 | -0.0471 |  |
|  |  | 350.487 | 39.672 |  | 0.0008 | 0.0008 | 0.0008 |  |
|  |  | 298.152 | 39.688 |  | 0.0563 | 0.0563 | -0.0563 |  |
|  |  | 298.165 | 39.707 |  | 0.0409 | 0.0409 | -0.0409 |  |
|  |  | 283.605 | 39.9 |  | 0.1861 | 0.1861 | -0.1861 |  |
|  |  | 332.158 | 39.991 |  | 0.0287 | 0.0287 | -0.0287 |  |
|  |  | 269.393 | 40.201 |  | 0.1982 | 0.1982 | -0.1982 |  |
|  |  | 298.174 | 40.239 |  | 0.0215 | 0.0215 | -0.0215 |  |
|  |  | 298.15 | 40.557 |  | 0.0767 | 0.0767 | -0.0767 |  |
|  |  | 255.994 | 40.643 |  | 0.1969 | 0.1969 | -0.1969 |  |
|  |  | 315.376 | 41.468 |  | 0.0524 | 0.0524 | -0.0524 |  |
|  |  | 243.677 | 41.792 |  | 0.2085 | 0.2085 | -0.2085 |  |
|  |  | 357.118 | 46.792 |  | 0.0082 | 0.0082 | 0.0082 |  |
|  |  | 305.929 | 49.286 |  | 0.0846 | 0.0846 | -0.0846 |  |
|  |  | 305.934 | 49.685 |  | 0.0537 | 0.0537 | -0.0537 |  |
|  |  | 248.82 | 49.885 |  | 0.2256 | 0.2256 | -0.2256 |  |
|  |  | 290.943 | 49.928 |  | 0.1997 | 0.1997 | -0.1997 |  |
|  |  | 340.92 | 49.956 |  | 0.0297 | 0.0297 | -0.0297 |  |
|  |  | 276.43 | 50.285 |  | 0.2156 | 0.2156 | -0.2156 |  |
|  |  | 322.713 | 50.376 |  | 0.0597 | 0.0597 | -0.0597 |  |
|  |  | 263.018 | 51.186 |  | 0.2176 | 0.2176 | -0.2176 |  |
|  |  | 330.586 | 59.794 |  | 0.0651 | 0.0651 | -0.0651 |  |
|  |  | 268.898 | 59.865 |  | 0.2374 | 0.2374 | -0.2374 |  |
|  |  | 349.884 | 60.027 |  | 0.0267 | 0.0267 | -0.0267 |  |
|  |  | 283.571 | 60.349 |  | 0.2351 | 0.2351 | -0.2351 |  |
|  |  | 314.421 | 60.539 |  | 0.0634 | 0.0634 | -0.0634 |  |
|  |  | 299.377 | 61.164 |  | 0.2190 | 0.2190 | -0.2190 |  |
| [C_6_mim][BF_4_] | REF 44 | 283.11 | 5.172 | for  T = 363.15 K  and  p = 0.1 MPa  0.67 % | 0.0049 | 0.0049 | 0.0049 | Density (p = 0.1 MPa) from REF 13, Isothermal Compressibility calculated from Tait equation from REF 13 |
|  |  | 283.13 | 1.008-10.574 |  | 0.0025 | 0.0040 | 0.0025 |  |
|  |  | 283.15 | 0.1-99.916 |  | 0.1549 | 0.2460 | -0.1537 |  |
|  |  | 283.16 | 19.82-60.112 |  | 0.0569 | 0.1094 | -0.0569 |  |
|  |  | 283.17 | 39.703 |  | 0.0516 | 0.0516 | -0.0516 |  |
|  |  | 293.12 | 79.946-89.985 |  | 0.1694 | 0.1904 | -0.1694 |  |
|  |  | 293.13 | 59.98-69.992 |  | 0.0950 | 0.1102 | -0.0950 |  |
|  |  | 293.14 | 99.711 |  | 0.2305 | 0.2305 | -0.2305 |  |
|  |  | 293.15 | 0.1-49.987 |  | 0.0289 | 0.0488 | -0.0036 |  |
|  |  | 293.17 | 30.004-39.55 |  | 0.0190 | 0.0306 | -0.0190 |  |
|  |  | 293.18 | 1.59-20.119 |  | 0.0093 | 0.0110 | 0.0093 |  |
|  |  | 298.14 | 5.026 |  | 0.0358 | 0.0358 | 0.0358 |  |
|  |  | 298.15 | 0.1-80.026 |  | 0.0371 | 0.1162 | -0.0085 |  |
|  |  | 298.16 | 10.325-99.412 |  | 0.0907 | 0.1850 | -0.0747 |  |
|  |  | 298.17 | 59.974 |  | 0.0544 | 0.0544 | -0.0544 |  |
|  |  | 313.11 | 5.26 |  | 0.0591 | 0.0591 | 0.0591 |  |
|  |  | 313.12 | 99.779 |  | 0.1153 | 0.1153 | -0.1153 |  |
|  |  | 313.13 | 60.061-89.987 |  | 0.0349 | 0.0747 | -0.0293 |  |
|  |  | 313.15 | 0.1-49.997 |  | 0.0392 | 0.0414 | 0.0392 |  |
|  |  | 313.16 | 20.356 |  | 0.0666 | 0.0666 | 0.0666 |  |
|  |  | 313.17 | 29.988 |  | 0.0557 | 0.0557 | 0.0557 |  |
|  |  | 313.18 | 1.626-39.88 |  | 0.0586 | 0.0704 | 0.0586 |  |
|  |  | 333.15 | 0.1-29.988 |  | 0.0516 | 0.0588 | 0.0516 |  |
|  |  | 333.16 | 5.212-100.083 |  | 0.0486 | 0.0596 | 0.0200 |  |
|  |  | 333.17 | 1.52-80.202 |  | 0.0253 | 0.0424 | 0.0253 |  |
|  |  | 353.13 | 80.219 |  | 0.0821 | 0.0821 | 0.0821 |  |
|  |  | 353.14 | 40.213-49.986 |  | 0.1295 | 0.1320 | 0.1295 |  |
|  |  | 353.15 | 0.1-89.982 |  | 0.0813 | 0.1265 | 0.0813 |  |
|  |  | 353.16 | 5.232-100.024 |  | 0.0708 | 0.1116 | 0.0708 |  |
|  |  | 363.15 | 0.1 |  | 0.6671 | 0.6671 | 0.6671 |  |
|  |  | 373.13 | 5.131-100.038 |  | 0.1030 | 0.1044 | 0.1030 |  |
|  |  | 373.14 | 1.516-89.993 |  | 0.1136 | 0.1385 | 0.1136 |  |
|  |  | 373.15 | 10.181-69.984 |  | 0.1613 | 0.1815 | 0.1613 |  |
|  |  | 373.16 | 20.124-60.059 |  | 0.1721 | 0.1847 | 0.1721 |  |
| [C_6_mim]Cl | REF 62 | 311.5 | 0.1-200 | for  T = 311.5 K  and  p = 200 MPa  0.31 % | 0.1323 | 0.3110 | -0.1323 | Isothermal Compressibility from REF 62 |
|  |  | 331.2 | 0.1-200 |  | 0.1057 | 0.2836 | -0.1047 |  |
|  |  | 351.2 | 0.1-200 |  | 0.0924 | 0.2303 | -0.0853 |  |
|  |  | 371.5 | 0.1-200 |  | 0.0856 | 0.2058 | -0.0848 |  |
|  |  | 391.6 | 0.1-200 |  | 0.0930 | 0.2108 | -0.0930 |  |
|  |  | 411.6 | 0.1-200 |  | 0.0841 | 0.1832 | -0.0841 |  |
|  |  | 431.4 | 0.1-200 |  | 0.0608 | 0.1481 | -0.0608 |  |
|  |  | 451.2 | 0.1-200 |  | 0.0290 | 0.0938 | -0.0135 |  |
| [C_6_mim][NTf_2_] | REF 56 | 298.15 | 0.1-59.59 | for  T = 298.15 K  and  p = 59.59 MPa  0.46 % | 0.1491 | 0.4589 | 0.1482 | Isothermal Compressibility from REF 56 |
|  |  | 299.15 | 0.1-59.59 |  | 0.1372 | 0.4219 | 0.1352 |  |
|  |  | 300.15 | 0.1-59.59 |  | 0.1266 | 0.3918 | 0.1266 |  |
|  |  | 301.15 | 0.1-59.59 |  | 0.1164 | 0.3593 | 0.1156 |  |
|  |  | 302.15 | 0.1-59.59 |  | 0.1050 | 0.3265 | 0.1050 |  |
|  |  | 303.15 | 0.1-59.59 |  | 0.0951 | 0.3011 | 0.0951 |  |
|  |  | 308.15 | 0.1-59.59 |  | 0.0593 | 0.1752 | 0.0593 |  |
|  |  | 313.15 | 0.1-59.59 |  | 0.0313 | 0.0734 | 0.0313 |  |
|  |  | 318.15 | 0.1-59.59 |  | 0.0094 | 0.0186 | 0.0076 |  |
|  |  | 323.15 | 0.1-59.59 |  | 0.0203 | 0.0692 | -0.0203 |  |
|  |  | 325.15 | 0.1-59.59 |  | 0.0582 | 0.1201 | -0.0582 |  |
|  |  | 327.15 | 0.1-59.59 |  | 0.0598 | 0.1522 | -0.0598 |  |
|  |  | 329.15 | 0.1-59.59 |  | 0.0571 | 0.1611 | -0.0571 |  |
|  |  | 331.15 | 0.1-59.59 |  | 0.0518 | 0.1644 | -0.0507 |  |
|  |  | 333.15 | 0.1-59.59 |  | 0.0540 | 0.1692 | -0.0455 |  |
| [C_6_mim][NTf_2_] | REF 60 | 298.15 | 0.1-40 | for  T = 423.15 K  and  p = 20 MPa  1.25 % | 0.0665 | 0.1919 | 0.0609 | Isothermal Compressibility from REF 56 |
|  |  | 323.15 | 0.1-40 |  | 0.0953 | 0.1572 | -0.0953 |  |
|  |  | 348.15 | 0.1-40 |  | 0.1335 | 0.2862 | -0.1117 |  |
|  |  | 373.15 | 0.1-40 |  | 0.0788 | 0.1546 | -0.0170 |  |
|  |  | 398.15 | 10-40 |  | 0.3660 | 0.4837 | 0.3660 |  |
|  |  | 423.15 | 10-40 |  | 0.9962 | 1.2323 | 0.9962 |  |
| [C_6_mim][NTf_2_] | REF 61 | 293.15 | 0.1-65.02 | for  T = 293.15 K  and  p = 65.02 MPa  0.76 % | 0.2756 | 0.7551 | 0.2710 | Isothermal Compressibility from REF 56 |
|  |  | 298.15 | 0.1-65.02 |  | 0.1934 | 0.5585 | 0.1934 |  |
|  |  | 303.15 | 0.1-65.02 |  | 0.1223 | 0.3707 | 0.1218 |  |
|  |  | 308.15 | 0.1-65.01 |  | 0.0683 | 0.2273 | 0.0683 |  |
|  |  | 313.15 | 0.1-65.02 |  | 0.0275 | 0.0969 | 0.0068 |  |
|  |  | 318.15 | 0.1-65 |  | 0.0459 | 0.0756 | -0.0439 |  |
|  |  | 323.15 | 0.1-65 |  | 0.1093 | 0.1672 | -0.1093 |  |
|  |  | 328.15 | 0.1-65.02 |  | 0.1516 | 0.2342 | -0.1516 |  |
|  |  | 333.15 | 0.1-65.02 |  | 0.1629 | 0.2871 | -0.1629 |  |
|  |  | 338.15 | 0.1-65.02 |  | 0.1541 | 0.3251 | -0.1500 |  |
| [C_6_mim][NTf_2_] | REF 62 | 312.6 | 0.1-200 | for  T = 312.6 K  and  p = 200 MPa  0.30 % | 0.1106 | 0.2973 | -0.1106 | Isothermal Compressibility from REF 62 |
|  |  | 332.4 | 0.1-200 |  | 0.0811 | 0.2262 | -0.0782 |  |
|  |  | 352.3 | 0.1-200 |  | 0.0665 | 0.1738 | -0.0648 |  |
|  |  | 372.5 | 0.1-200 |  | 0.0404 | 0.1202 | -0.0399 |  |
|  |  | 392.7 | 0.1-200 |  | 0.0321 | 0.0953 | -0.0321 |  |
|  |  | 412.7 | 0.1-200 |  | 0.0202 | 0.0454 | -0.0119 |  |
|  |  | 432.6 | 0.1-200 |  | 0.0626 | 0.1032 | 0.0622 |  |
|  |  | 452.3 | 0.1-200 |  | 0.1152 | 0.1768 | 0.1152 |  |
| [C_6_mim][NTf_2_] | REF 63 | 273.13 | 50.004-99.924 | for  T = 413.15 K  and  p = 139.957 MPa  0.51 % | 0.0612 | 0.0672 | -0.0612 | Isothermal Compressibility from REF 62 |
|  |  | 273.14 | 20.001-120.002 |  | 0.0530 | 0.0678 | -0.0530 |  |
|  |  | 273.15 | 0.1-139.957 |  | 0.0424 | 0.0697 | -0.0404 |  |
|  |  | 273.16 | 5.032 |  | 0.0198 | 0.0198 | -0.0198 |  |
|  |  | 273.17 | 89.925 |  | 0.0622 | 0.0622 | -0.0622 |  |
|  |  | 283.12 | 1.145 |  | 0.0068 | 0.0068 | -0.0068 |  |
|  |  | 283.13 | 5.278 |  | 0.0147 | 0.0147 | -0.0147 |  |
|  |  | 283.14 | 19.635-50.099 |  | 0.0535 | 0.0663 | -0.0535 |  |
|  |  | 283.15 | 0.1-139.945 |  | 0.0474 | 0.0700 | -0.0474 |  |
|  |  | 283.16 | 5.245-80.499 |  | 0.0608 | 0.0705 | -0.0608 |  |
|  |  | 283.18 | 1.894 |  | 0.0121 | 0.0121 | -0.0121 |  |
|  |  | 293.14 | 79.829-99.324 |  | 0.0649 | 0.0685 | -0.0649 |  |
|  |  | 293.15 | 0.1-139.456 |  | 0.0391 | 0.0702 | -0.0383 |  |
|  |  | 293.16 | 10.023-129.586 |  | 0.0601 | 0.0698 | -0.0601 |  |
|  |  | 293.17 | 1.593-29.922 |  | 0.0290 | 0.0498 | -0.0290 |  |
|  |  | 293.18 | 5.133 |  | 0.0147 | 0.0147 | -0.0147 |  |
|  |  | 293.19 | 20.053 |  | 0.0383 | 0.0383 | -0.0383 |  |
|  |  | 293.2 | 10.122 |  | 0.0242 | 0.0242 | -0.0242 |  |
|  |  | 298.13 | 29.975 |  | 0.0472 | 0.0472 | -0.0472 |  |
|  |  | 298.14 | 10.002-89.928 |  | 0.0507 | 0.0703 | -0.0507 |  |
|  |  | 298.15 | 0.1-139.964 |  | 0.0419 | 0.0699 | -0.0411 |  |
|  |  | 298.16 | 5.044-89.921 |  | 0.0530 | 0.0666 | -0.0530 |  |
|  |  | 298.17 | 69.856 |  | 0.0715 | 0.0715 | -0.0715 |  |
|  |  | 313.12 | 19.686 |  | 0.0299 | 0.0299 | -0.0299 |  |
|  |  | 313.13 | 1.688-9.595 |  | 0.0086 | 0.0152 | -0.0086 |  |
|  |  | 313.14 | 69.895-89.696 |  | 0.0656 | 0.0684 | -0.0656 |  |
|  |  | 313.15 | 0.1-139.957 |  | 0.0477 | 0.0695 | -0.0462 |  |
|  |  | 313.16 | 4.693-129.937 |  | 0.0345 | 0.0674 | -0.0345 |  |
|  |  | 313.18 | 1.996-10.212 |  | 0.0121 | 0.0188 | -0.0121 |  |
|  |  | 313.22 | 29.205 |  | 0.0490 | 0.0490 | -0.0490 |  |
|  |  | 333.14 | 10.35-20.181 |  | 0.0252 | 0.0306 | -0.0252 |  |
|  |  | 333.15 | 0.1-139.984 |  | 0.0362 | 0.0656 | -0.0306 |  |
|  |  | 333.16 | 1.276-69.539 |  | 0.0350 | 0.0657 | -0.0350 |  |
|  |  | 333.17 | 39.844 |  | 0.0571 | 0.0571 | -0.0571 |  |
|  |  | 353.13 | 59.694 |  | 0.0584 | 0.0584 | -0.0584 |  |
|  |  | 353.14 | 69.979 |  | 0.0526 | 0.0526 | -0.0526 |  |
|  |  | 353.15 | 0.1-129.957 |  | 0.0355 | 0.0770 | -0.0142 |  |
|  |  | 353.16 | 2.199-139.6 |  | 0.0356 | 0.1044 | -0.0164 |  |
|  |  | 353.17 | 19.921 |  | 0.0339 | 0.0339 | -0.0339 |  |
|  |  | 353.18 | 0.939 |  | 0.0006 | 0.0006 | -0.0006 |  |
|  |  | 373.13 | 19.682-49.98 |  | 0.0453 | 0.0564 | -0.0453 |  |
|  |  | 373.14 | 10.099-130.002 |  | 0.0442 | 0.1665 | 0.0155 |  |
|  |  | 373.15 | 0.1-139.974 |  | 0.0530 | 0.2103 | 0.0238 |  |
|  |  | 373.16 | 1.688-60.001 |  | 0.0393 | 0.0573 | -0.0393 |  |
|  |  | 373.17 | 40.202-98.843 |  | 0.0381 | 0.0585 | -0.0182 |  |
|  |  | 373.18 | 90.239 |  | 0.0110 | 0.0110 | 0.0110 |  |
|  |  | 393.12 | 9.618 |  | 0.0199 | 0.0199 | -0.0199 |  |
|  |  | 393.13 | 19.936 |  | 0.0601 | 0.1021 | 0.0080 |  |
|  |  | 393.14 | 98.655 |  | 0.0366 | 0.1070 | 0.0098 |  |
|  |  | 393.15 | 1.318-99.655 |  | 0.1074 | 0.3424 | 0.0533 |  |
|  |  | 393.16 | 0.1-139.923 |  | 0.0409 | 0.0571 | -0.0124 |  |
|  |  | 393.17 | 5.148-89.747 |  | 0.1522 | 0.2797 | 0.1274 |  |
|  |  | 393.18 | 10.147-180.003 |  | 0.0115 | 0.0154 | -0.0115 |  |
|  |  | 413.13 | 2.367-70.205 |  | 0.2233 | 0.4225 | 0.1843 |  |
|  |  | 413.14 | 39.958-129.958 |  | 0.1953 | 0.3408 | 0.1455 |  |
|  |  | 413.15 | 20.001-120.002 |  | 0.1092 | 0.5047 | 0.0715 |  |
|  |  | 413.16 | 0.1-139.957 |  | 0.0406 | 0.0634 | 0.0228 |  |
|  |  | 413.17 | 5.032-80.001 |  | 0.0222 | 0.0222 | -0.0222 |  |
| [C_6_mim][NTf_2_] | REF 81 | 273.15 | 0.1 | for  T = 288.15 K  and  p = 174.6 MPa  0.38 % | 0.0005 | 0.0005 | -0.0005 | Isothermal Compressibility from REF 62 |
|  |  | 278.15 | 0.1 |  | 0.0014 | 0.0014 | -0.0014 |  |
|  |  | 283.15 | 0.1 |  | 0.0010 | 0.0010 | -0.0010 |  |
|  |  | 288.15 | 0.1 |  | 0.0001 | 0.0001 | 0.0001 |  |
|  |  | 293.15 | 0.1 |  | 0.0004 | 0.0004 | 0.0004 |  |
|  |  | 293.15 | 0.1 |  | 0.0018 | 0.0018 | 0.0018 |  |
|  |  | 298.15 | 0.1 |  | 0.0020 | 0.0020 | 0.0020 |  |
|  |  | 303.15 | 0.1 |  | 0.0013 | 0.0013 | 0.0013 |  |
|  |  | 313.15 | 0.1 |  | 0.0003 | 0.0003 | 0.0003 |  |
|  |  | 323.15 | 0.1 |  | 0.0011 | 0.0011 | -0.0011 |  |
|  |  | 333.15 | 0.1 |  | 0.0021 | 0.0021 | -0.0021 |  |
|  |  | 343.15 | 0.1 |  | 0.0014 | 0.0014 | -0.0014 |  |
|  |  | 353.15 | 0.1 |  | 0.0003 | 0.0003 | -0.0003 |  |
|  |  | 363.15 | 0.1 |  | 0.0019 | 0.0019 | 0.0019 |  |
|  |  | 288.15 | 14.95-174.6 |  | 0.2096 | 0.3862 | -0.2096 |  |
| [C_6_py][BF_4_] | REF 20 | 293.15 | 0.1-20 | for  T = 313.15 K  and  p = 20 MPa  0.09 % | 0.0343 | 0.0635 | 0.0343 | Isothermal Compressibility calculated from Tait equation from REF 20 |
|  |  | 303.15 | 0.1-20 |  | 0.0366 | 0.0835 | 0.0359 |  |
|  |  | 313.15 | 0.1-20 |  | 0.0394 | 0.0879 | 0.0394 |  |
|  |  | 323.15 | 0.1-20 |  | 0.0361 | 0.0841 | 0.0268 |  |
|  |  | 333.15 | 0.1-20 |  | 0.0419 | 0.0800 | 0.0419 |  |
|  |  | 343.15 | 0.1-20 |  | 0.0280 | 0.0576 | 0.0280 |  |
|  |  | 353.15 | 0.1-20 |  | 0.0081 | 0.0148 | -0.0081 |  |
| [C_7_mim][NTf_2_] | REF 11 | 293.15 | 0.1-30 | for  T = 393.15 K  and  p = 30 MPa  0.20 % | 0.0299 | 0.1110 | -0.0299 | Isothermal Compressibility from REF 11 |
|  |  | 303.15 | 0.1-30 |  | 0.0244 | 0.1118 | -0.0224 |  |
|  |  | 313.15 | 0.1-30 |  | 0.0255 | 0.1094 | -0.0168 |  |
|  |  | 323.15 | 0.1-30 |  | 0.0253 | 0.1038 | -0.0127 |  |
|  |  | 333.15 | 0.1-30 |  | 0.0560 | 0.1549 | -0.0560 |  |
|  |  | 343.15 | 0.1-30 |  | 0.0209 | 0.0907 | -0.0108 |  |
|  |  | 353.15 | 0.1-30 |  | 0.0316 | 0.1439 | -0.0271 |  |
|  |  | 393.15 | 0.1-30 |  | 0.0444 | 0.2020 | -0.0429 |  |
| [C_8_py][BF_4_] | REF 20 | 293.15 | 0.1-20 | for  T = 313.15 K  and  p = 20 MPa  0.08 % | 0.0275 | 0.0615 | 0.0275 | Isothermal Compressibility calculated from Tait equation from REF 20 |
|  |  | 303.15 | 0.1-20 |  | 0.0255 | 0.0646 | 0.0250 |  |
|  |  | 313.15 | 0.1-20 |  | 0.0344 | 0.0787 | 0.0344 |  |
|  |  | 323.15 | 0.1-20 |  | 0.0268 | 0.0670 | 0.0209 |  |
|  |  | 333.15 | 0.1-20 |  | 0.0281 | 0.0561 | 0.0281 |  |
|  |  | 343.15 | 0.1-20 |  | 0.0192 | 0.0362 | 0.0192 |  |
|  |  | 353.15 | 0.1-20 |  | 0.0022 | 0.0034 | -0.0008 |  |
| [N_1112OH_][Lactate] | REF 8 | 293.15 | 0.1 | for  T = 392.4 K  and  p = 10 MPa  0.25 % | 0.0016 | 0.0016 | 0.0016 | Isothermal Compressibility from REF 8 |
|  |  | 298.15 | 0.1 |  | 0.0060 | 0.0060 | 0.0060 |  |
|  |  | 303.15 | 0.1 |  | 0.0044 | 0.0044 | -0.0044 |  |
|  |  | 308.15 | 0.1 |  | 0.0030 | 0.0030 | -0.0030 |  |
|  |  | 313.15 | 0.1 |  | 0.0076 | 0.0076 | -0.0076 |  |
|  |  | 318.15 | 0.1 |  | 0.0005 | 0.0005 | -0.0005 |  |
|  |  | 323.15 | 0.1 |  | 0.0007 | 0.0007 | 0.0007 |  |
|  |  | 328.15 | 0.1 |  | 0.0049 | 0.0049 | 0.0049 |  |
|  |  | 333.15 | 0.1 |  | 0.0031 | 0.0031 | 0.0031 |  |
|  |  | 338.15 | 0.1 |  | 0.0042 | 0.0042 | 0.0042 |  |
|  |  | 343.15 | 0.1 |  | 0.0007 | 0.0007 | -0.0007 |  |
|  |  | 348.15 | 0.1 |  | 0.0027 | 0.0027 | -0.0027 |  |
|  |  | 353.15 | 0.1 |  | 0.0017 | 0.0017 | -0.0017 |  |
|  |  | 312.4 | 10-200 |  | 0.0475 | 0.1073 | 0.0404 |  |
|  |  | 332.3 | 10-200 |  | 0.0679 | 0.1342 | 0.0582 |  |
|  |  | 352.2 | 10-200 |  | 0.0817 | 0.1377 | 0.0762 |  |
|  |  | 372.3 | 10-200 |  | 0.0957 | 0.1844 | 0.0925 |  |
|  |  | 392.4 | 10-200 |  | 0.1345 | 0.2520 | 0.1302 |  |
| [N_1114_][NTf_2_] | REF 53 | 293.48 | 0.1 | for  T = 414.93 K  and  p = 40 MPa  0.09 % | 0.0055 | 0.0055 | -0.0055 | Isothermal Compressibility from REF 53 |
|  |  | 302.84 | 0.1 |  | 0.0004 | 0.0004 | -0.0004 |  |
|  |  | 312.82 | 0.1 |  | 0.0016 | 0.0016 | 0.0016 |  |
|  |  | 322.33 | 0.1 |  | 0.0054 | 0.0054 | 0.0054 |  |
|  |  | 332.75 | 0.1 |  | 0.0020 | 0.0020 | 0.0020 |  |
|  |  | 342.67 | 0.1 |  | 0.0024 | 0.0024 | 0.0024 |  |
|  |  | 352.36 | 0.1 |  | 0.0036 | 0.0036 | 0.0036 |  |
|  |  | 373.31 | 0.1 |  | 0.0062 | 0.0062 | -0.0062 |  |
|  |  | 391.28 | 0.1 |  | 0.0114 | 0.0114 | -0.0114 |  |
|  |  | 414.93 | 0.1 |  | 0.0084 | 0.0084 | 0.0084 |  |
|  |  | 293.51 | 1 |  | 0.0070 | 0.0070 | -0.0070 |  |
|  |  | 322.33 | 1 |  | 0.0001 | 0.0001 | -0.0001 |  |
|  |  | 352.35 | 1 |  | 0.0043 | 0.0043 | -0.0043 |  |
|  |  | 373.32 | 1 |  | 0.0122 | 0.0122 | -0.0122 |  |
|  |  | 391.26 | 1 |  | 0.0201 | 0.0201 | -0.0201 |  |
|  |  | 414.94 | 1 |  | 0.0018 | 0.0018 | 0.0018 |  |
|  |  | 293.51 | 5 |  | 0.0004 | 0.0004 | -0.0004 |  |
|  |  | 322.32 | 5 |  | 0.0012 | 0.0012 | -0.0012 |  |
|  |  | 352.33 | 5 |  | 0.0072 | 0.0072 | -0.0072 |  |
|  |  | 373.31 | 5 |  | 0.0011 | 0.0011 | -0.0011 |  |
|  |  | 391.3 | 5 |  | 0.0061 | 0.0061 | 0.0061 |  |
|  |  | 414.93 | 5 |  | 0.0112 | 0.0112 | 0.0112 |  |
|  |  | 293.5 | 10 |  | 0.0033 | 0.0033 | -0.0033 |  |
|  |  | 322.31 | 10 |  | 0.0053 | 0.0053 | -0.0053 |  |
|  |  | 352.32 | 10 |  | 0.0071 | 0.0071 | -0.0071 |  |
|  |  | 373.33 | 10 |  | 0.0076 | 0.0076 | -0.0076 |  |
|  |  | 391.29 | 10 |  | 0.0028 | 0.0028 | -0.0028 |  |
|  |  | 414.93 | 10 |  | 0.0022 | 0.0022 | -0.0022 |  |
|  |  | 293.5 | 20 |  | 0.0177 | 0.0177 | -0.0177 |  |
|  |  | 322.31 | 20 |  | 0.0224 | 0.0224 | -0.0224 |  |
|  |  | 352.31 | 20 |  | 0.0226 | 0.0226 | -0.0226 |  |
|  |  | 373.34 | 20 |  | 0.0206 | 0.0206 | -0.0206 |  |
|  |  | 391.32 | 20 |  | 0.0247 | 0.0247 | -0.0247 |  |
|  |  | 414.93 | 20 |  | 0.0273 | 0.0273 | -0.0273 |  |
|  |  | 293.5 | 30 |  | 0.0389 | 0.0389 | -0.0389 |  |
|  |  | 322.32 | 30 |  | 0.0358 | 0.0358 | -0.0358 |  |
|  |  | 352.33 | 30 |  | 0.0406 | 0.0406 | -0.0406 |  |
|  |  | 373.31 | 30 |  | 0.0407 | 0.0407 | -0.0407 |  |
|  |  | 391.29 | 30 |  | 0.0442 | 0.0442 | -0.0442 |  |
|  |  | 414.93 | 30 |  | 0.0527 | 0.0527 | -0.0527 |  |
|  |  | 293.5 | 40 |  | 0.0650 | 0.0650 | -0.0650 |  |
|  |  | 322.31 | 40 |  | 0.0624 | 0.0624 | -0.0624 |  |
|  |  | 352.31 | 40 |  | 0.0707 | 0.0707 | -0.0707 |  |
|  |  | 373.34 | 40 |  | 0.0745 | 0.0745 | -0.0745 |  |
|  |  | 391.32 | 40 |  | 0.0831 | 0.0831 | -0.0831 |  |
|  |  | 414.93 | 40 |  | 0.0917 | 0.0917 | -0.0917 |  |
| [o3mpy][BF_4_] | REF 75 | 283.15 | 0.1-65 | for  T = 328.15 K  and  p = 60 MPa  0.06 % | 0.0184 | 0.0492 | 0.0184 | Isothermal Compressibility from REF 75 |
|  |  | 288.15 | 0.1-65 |  | 0.0217 | 0.0531 | 0.0216 |  |
|  |  | 293.15 | 0.1-65 |  | 0.0141 | 0.0412 | 0.0065 |  |
|  |  | 298.15 | 0.1-65 |  | 0.0195 | 0.0453 | 0.0195 |  |
|  |  | 303.15 | 0.1-65 |  | 0.0137 | 0.0409 | 0.0103 |  |
|  |  | 308.15 | 0.1-65 |  | 0.0236 | 0.0445 | 0.0236 |  |
|  |  | 313.15 | 0.1-65 |  | 0.0204 | 0.0485 | 0.0204 |  |
|  |  | 318.15 | 0.1-65 |  | 0.0248 | 0.0553 | 0.0248 |  |
|  |  | 323.15 | 0.1-65 |  | 0.0216 | 0.0551 | 0.0206 |  |
|  |  | 328.15 | 0.1-65 |  | 0.0281 | 0.0578 | 0.0279 |  |
|  |  | 333.15 | 0.1-65 |  | 0.0218 | 0.0519 | 0.0213 |  |
| [P_666 14_][DCA] | REF 66 | 273.15 | 0.1-35 | for  T = 273.15 K  and  p = 35 MPa  0.07 % | 0.0550 | 0.0720 | -0.0550 | Isothermal Compressibility from REF 66 |
|  |  | 278.15 | 0.1-35 |  | 0.0510 | 0.0698 | -0.0370 |  |
|  |  | 283.15 | 0.1-35 |  | 0.0269 | 0.0454 | -0.0269 |  |
|  |  | 288.15 | 0.1-35 |  | 0.0221 | 0.0463 | -0.0215 |  |
|  |  | 293.15 | 0.1-35 |  | 0.0322 | 0.0635 | -0.0298 |  |
|  |  | 298.15 | 0.1-35 |  | 0.0156 | 0.0332 | -0.0143 |  |
|  |  | 303.15 | 0.1-35 |  | 0.0227 | 0.0422 | -0.0220 |  |
|  |  | 308.15 | 0.1-35 |  | 0.0259 | 0.0425 | -0.0218 |  |
|  |  | 313.15 | 0.1-35 |  | 0.0182 | 0.0363 | -0.0182 |  |
|  |  | 318.15 | 0.1-35 |  | 0.0254 | 0.0467 | -0.0254 |  |
| [P_666 14_][DCA] | REF 67 | 283.15 | 0.1-45 | for  T = 283.15 K  and  p = 45 MPa  0.03 % | 0.0128 | 0.0285 | -0.0128 | Isothermal Compressibility from REF 67 |
|  |  | 293.15 | 0.1-45 |  | 0.0054 | 0.0237 | -0.0037 |  |
|  |  | 303.15 | 0.1-45 |  | 0.0066 | 0.0153 | -0.0061 |  |
|  |  | 313.15 | 0.1-45 |  | 0.0081 | 0.0250 | -0.0076 |  |
|  |  | 323.15 | 0.1-45 |  | 0.0070 | 0.0251 | -0.0055 |  |
|  |  | 333.15 | 0.1-45 |  | 0.0095 | 0.0202 | -0.0095 |  |
| [P_666 14_]Br | REF 67 | 283.15 | 0.1-45 | for  T = 323.15 K  and  p = 45 MPa  0.14 % | 0.0387 | 0.1054 | -0.0386 | Isothermal Compressibility from REF 67 |
|  |  | 293.15 | 0.1-45 |  | 0.0431 | 0.1203 | -0.0431 |  |
|  |  | 303.15 | 0.1-45 |  | 0.0493 | 0.1296 | -0.0493 |  |
|  |  | 313.15 | 0.1-45 |  | 0.0460 | 0.1225 | -0.0460 |  |
|  |  | 323.15 | 0.1-45 |  | 0.0507 | 0.1400 | -0.0492 |  |
|  |  | 333.15 | 0.1-45 |  | 0.0523 | 0.1299 | -0.0523 |  |
| [P_666 14_][C_1_SO_3_] | REF 67 | 283.15 | 0.1-45 | for  T = 283.15 K  and  p = 45 MPa  0.03 % | 0.0091 | 0.0344 | -0.0047 |  |
|  |  | 293.15 | 0.1-45 |  | 0.0085 | 0.0264 | -0.0006 |  |
|  |  | 303.15 | 0.1-45 |  | 0.0060 | 0.0143 | -0.0019 |  |
|  |  | 313.15 | 0.1-45 |  | 0.0038 | 0.0192 | -0.0016 |  |
|  |  | 323.15 | 0.1-45 |  | 0.0058 | 0.0202 | -0.0029 |  |
|  |  | 333.15 | 0.1-45 |  | 0.0068 | 0.0149 | -0.0044 |  |
| [P_666 14_][(C_2_F_5_)_3_PF_3_] | REF 69 | 293.2 | 0.1 | for  T = 323.15 K  and  p = 20 MPa  0.12 % | 0.0056 | 0.0056 | 0.0056 | Isothermal Compressibility from REF 74 |
|  |  | 303.19 | 0.1 |  | 0.0065 | 0.0065 | -0.0065 |  |
|  |  | 313.15 | 0.1 |  | 0.0087 | 0.0087 | -0.0087 |  |
|  |  | 323.16 | 0.1 |  | 0.0110 | 0.0110 | 0.0110 |  |
|  |  | 333.11 | 0.1 |  | 0.0000 | 0.0000 | 0.0000 |  |
|  |  | 343.13 | 0.1 |  | 0.0008 | 0.0008 | 0.0008 |  |
|  |  | 353.2 | 0.1 |  | 0.0022 | 0.0022 | -0.0022 |  |
|  |  | 293.21 | 0.5 |  | 0.0125 | 0.0125 | 0.0125 |  |
|  |  | 303.19 | 0.5 |  | 0.0062 | 0.0062 | -0.0062 |  |
|  |  | 313.17 | 0.5 |  | 0.0086 | 0.0086 | -0.0086 |  |
|  |  | 323.15 | 0.5 |  | 0.0058 | 0.0058 | 0.0058 |  |
|  |  | 333.12 | 0.5 |  | 0.0033 | 0.0033 | 0.0033 |  |
|  |  | 343.07 | 0.5 |  | 0.0070 | 0.0070 | -0.0070 |  |
|  |  | 353.22 | 0.5 |  | 0.0143 | 0.0143 | -0.0143 |  |
|  |  | 293.22 | 1 |  | 0.0423 | 0.0423 | 0.0423 |  |
|  |  | 303.19 | 1 |  | 0.0259 | 0.0259 | 0.0259 |  |
|  |  | 313.17 | 1 |  | 0.0253 | 0.0253 | 0.0253 |  |
|  |  | 323.15 | 1 |  | 0.0328 | 0.0328 | 0.0328 |  |
|  |  | 333.12 | 1 |  | 0.0321 | 0.0321 | 0.0321 |  |
|  |  | 343.07 | 1 |  | 0.0236 | 0.0236 | 0.0236 |  |
|  |  | 353.23 | 1 |  | 0.0176 | 0.0176 | 0.0176 |  |
|  |  | 293.22 | 2.5 |  | 0.0658 | 0.0658 | 0.0658 |  |
|  |  | 303.21 | 2.5 |  | 0.0523 | 0.0523 | 0.0523 |  |
|  |  | 313.17 | 2.5 |  | 0.0490 | 0.0490 | 0.0490 |  |
|  |  | 323.16 | 2.5 |  | 0.0605 | 0.0605 | 0.0605 |  |
|  |  | 333.12 | 2.5 |  | 0.0567 | 0.0567 | 0.0567 |  |
|  |  | 343.08 | 2.5 |  | 0.0526 | 0.0526 | 0.0526 |  |
|  |  | 353.22 | 2.5 |  | 0.0446 | 0.0446 | 0.0446 |  |
|  |  | 293.22 | 5 |  | 0.0805 | 0.0805 | 0.0805 |  |
|  |  | 303.22 | 5 |  | 0.0731 | 0.0731 | 0.0731 |  |
|  |  | 313.17 | 5 |  | 0.0691 | 0.0691 | 0.0691 |  |
|  |  | 323.15 | 5 |  | 0.0801 | 0.0801 | 0.0801 |  |
|  |  | 333.13 | 5 |  | 0.0038 | 0.0038 | -0.0038 |  |
|  |  | 343.09 | 5 |  | 0.0692 | 0.0692 | 0.0692 |  |
|  |  | 353.22 | 5 |  | 0.0703 | 0.0703 | 0.0703 |  |
|  |  | 293.22 | 10 |  | 0.1193 | 0.1193 | 0.1193 |  |
|  |  | 303.22 | 10 |  | 0.1081 | 0.1081 | 0.1081 |  |
|  |  | 313.17 | 10 |  | 0.1005 | 0.1005 | 0.1005 |  |
|  |  | 323.15 | 10 |  | 0.1170 | 0.1170 | 0.1170 |  |
|  |  | 333.12 | 10 |  | 0.1175 | 0.1175 | 0.1175 |  |
|  |  | 343.08 | 10 |  | 0.1105 | 0.1105 | 0.1105 |  |
|  |  | 353.23 | 10 |  | 0.1089 | 0.1089 | 0.1089 |  |
|  |  | 293.23 | 15 |  | 0.1148 | 0.1148 | 0.1148 |  |
|  |  | 303.23 | 15 |  | 0.1071 | 0.1071 | 0.1071 |  |
|  |  | 313.17 | 15 |  | 0.0957 | 0.0957 | 0.0957 |  |
|  |  | 323.14 | 15 |  | 0.1171 | 0.1171 | 0.1171 |  |
|  |  | 333.13 | 15 |  | 0.1211 | 0.1211 | 0.1211 |  |
|  |  | 343.08 | 15 |  | 0.1113 | 0.1113 | 0.1113 |  |
|  |  | 353.23 | 15 |  | 0.1155 | 0.1155 | 0.1155 |  |
|  |  | 293.23 | 20 |  | 0.1109 | 0.1109 | 0.1109 |  |
|  |  | 303.23 | 20 |  | 0.1061 | 0.1061 | 0.1061 |  |
|  |  | 313.17 | 20 |  | 0.0979 | 0.0979 | 0.0979 |  |
|  |  | 323.15 | 20 |  | 0.1221 | 0.1221 | 0.1221 |  |
|  |  | 333.14 | 20 |  | 0.1215 | 0.1215 | 0.1215 |  |
|  |  | 343.1 | 20 |  | 0.1154 | 0.1154 | 0.1154 |  |
|  |  | 353.25 | 20 |  | 0.1159 | 0.1159 | 0.1159 |  |
|  |  | 293.22 | 25 |  | 0.1080 | 0.1080 | 0.1080 |  |
|  |  | 303.25 | 25 |  | 0.1036 | 0.1036 | 0.1036 |  |
|  |  | 313.17 | 25 |  | 0.0992 | 0.0992 | 0.0992 |  |
|  |  | 323.16 | 25 |  | 0.1171 | 0.1171 | 0.1171 |  |
|  |  | 333.13 | 25 |  | 0.1124 | 0.1124 | 0.1124 |  |
|  |  | 343.09 | 25 |  | 0.1182 | 0.1182 | 0.1182 |  |
|  |  | 353.25 | 25 |  | 0.1220 | 0.1220 | 0.1220 |  |
| [P_666 14_][(C_2_F_5_)_3_PF_3_] | REF 70 | 293.15 | 0.1-25 | for  T = 313.15 K  and  p = 25 MPa  0.04 % | 0.0123 | 0.0340 | -0.0119 | Isothermal Compressibility from REF 70 |
|  |  | 298.15 | 0.1-25 |  | 0.0141 | 0.0352 | -0.0141 |  |
|  |  | 303.15 | 0.1-25 |  | 0.0163 | 0.0371 | -0.0163 |  |
|  |  | 308.15 | 0.1-25 |  | 0.0156 | 0.0398 | -0.0153 |  |
|  |  | 313.15 | 0.1-25 |  | 0.0157 | 0.0433 | -0.0154 |  |
|  |  | 318.15 | 0.1-25 |  | 0.0161 | 0.0392 | -0.0155 |  |
|  |  | 323.15 | 0.1-25 |  | 0.0145 | 0.0358 | -0.0135 |  |
|  |  | 328.15 | 0.1-25 |  | 0.0141 | 0.0332 | -0.0126 |  |
|  |  | 333.15 | 0.1-25 |  | 0.0139 | 0.0313 | -0.0116 |  |
|  |  | 338.15 | 0.1-25 |  | 0.0105 | 0.0231 | -0.0096 |  |
|  |  | 343.15 | 0.1-25 |  | 0.0099 | 0.0213 | -0.0065 |  |
| [P_666 14_][(C_2_F_5_)_3_PF_3_] | REF 74 | 278.15 | 0.1-120 | for  T = 278.15 K  and  p = 120 MPa  0.17 % | 0.0631 | 0.1751 | -0.0405 | Isothermal Compressibility from REF 74 |
|  |  | 298.15 | 0.1-120 |  | 0.0571 | 0.1717 | -0.0486 |  |
|  |  | 313.15 | 0.1-120 |  | 0.0451 | 0.1320 | -0.0419 |  |
|  |  | 333.15 | 0.1-120 |  | 0.0220 | 0.0664 | -0.0108 |  |
|  |  | 348.15 | 0.1-120 |  | 0.0090 | 0.0238 | -0.0036 |  |
|  |  | 373.15 | 0.1-120 |  | 0.0383 | 0.0896 | 0.0316 |  |
|  |  | 398.15 | 0.1-120 |  | 0.0705 | 0.1711 | 0.0386 |  |
| [P_666 14_][OAc] | REF 65 | 298.15 | 0.21-65.01 | for  T = 323.96 K  and  p = 25.01 MPa  0.65 % | 0.5955 | 0.6132 | -0.5955 | Density and Isothermal Compressibility calculated using the GCM method proposed in REF 53 |
|  |  | 303.15 | 0.21-65.01 |  | 0.6016 | 0.6207 | -0.6016 |  |
|  |  | 307.97 | 0.21-65.01 |  | 0.6089 | 0.6313 | -0.6089 |  |
|  |  | 313.07 | 0.21-65.01 |  | 0.6135 | 0.6435 | -0.6135 |  |
|  |  | 317.74 | 0.21-65.01 |  | 0.6206 | 0.6469 | -0.6206 |  |
|  |  | 323.96 | 0.21-65.01 |  | 0.6273 | 0.6508 | -0.6273 |  |
|  |  | 329.14 | 0.21-65.01 |  | 0.6230 | 0.6420 | -0.6230 |  |
|  |  | 334.11 | 0.21-65.01 |  | 0.6184 | 0.6333 | -0.6184 |  |
| [P_666 14_][NTf_2_] | REF 65 | 298.15 | 0.21-65.01 | for  T = 318.27 K  and  p = 65.01 MPa  0.11 % | 0.0083 | 0.0184 | -0.0062 | Density (p = 0.1 MPa), Isothermal Compressibility from REF 67 |
|  |  | 303.25 | 0.21-65.01 |  | 0.0169 | 0.0272 | 0.0169 |  |
|  |  | 308.15 | 0.21-65.01 |  | 0.0310 | 0.0404 | 0.0310 |  |
|  |  | 318.27 | 0.21-65.01 |  | 0.0531 | 0.1049 | 0.0531 |  |
|  |  | 323.33 | 0.21-65.01 |  | 0.0475 | 0.0746 | 0.0475 |  |
|  |  | 328.39 | 0.21-65.01 |  | 0.0389 | 0.0786 | 0.0311 |  |
|  |  | 333.43 | 0.21-65.01 |  | 0.0416 | 0.0795 | 0.0116 |  |
| [P_666 14_][NTf_2_] | REF 67 | 283.15 | 0.1-45 | for  T = 283.15 K  and  p = 45 MPa  0.17 % | 0.0643 | 0.1660 | 0.0642 | Isothermal Compressibility from REF 67 |
|  |  | 293.15 | 0.1-45 |  | 0.0620 | 0.1550 | 0.0620 |  |
|  |  | 303.15 | 0.1-45 |  | 0.0535 | 0.1400 | 0.0531 |  |
|  |  | 313.15 | 0.1-45 |  | 0.0544 | 0.1486 | 0.0537 |  |
|  |  | 323.15 | 0.1-45 |  | 0.0572 | 0.1441 | 0.0572 |  |
|  |  | 333.15 | 0.1-45 |  | 0.0535 | 0.1453 | 0.0528 |  |
| [P_666 14_]Cl | REF 65 | 298.13 | 0.19-65 | for  T = 333.14 K  and  p = 65 MPa  0.21 % | 0.0740 | 0.0891 | -0.0740 | Density (p = 0.1 MPa), Isothermal Compressibility from REF 66 |
|  |  | 303.15 | 0.19-65 |  | 0.0872 | 0.1064 | -0.0872 |  |
|  |  | 308.13 | 0.19-65 |  | 0.1156 | 0.1389 | -0.1156 |  |
|  |  | 313.15 | 0.19-55 |  | 0.1392 | 0.1680 | -0.1392 |  |
|  |  | 318.15 | 0.19-65 |  | 0.1595 | 0.1904 | -0.1595 |  |
|  |  | 323.14 | 0.19-65 |  | 0.1459 | 0.1850 | -0.1459 |  |
|  |  | 328.16 | 0.19-65 |  | 0.1612 | 0.2079 | -0.1612 |  |
|  |  | 333.14 | 0.19-65 |  | 0.1460 | 0.2148 | -0.1460 |  |
| [P_666 14_]Cl | REF 66 | 273.15 | 0.1-25 | for  T = 288.15 K  and  p = 25 MPa  0.06 % | 0.0224 | 0.0534 | -0.0082 | Isothermal Compressibility from REF 66 |
|  |  | 278.15 | 0.1-25 |  | 0.0238 | 0.0539 | -0.0060 |  |
|  |  | 283.15 | 0.1-25 |  | 0.0230 | 0.0432 | -0.0007 |  |
|  |  | 288.15 | 0.1-25 |  | 0.0234 | 0.0627 | 0.0234 |  |
|  |  | 293.15 | 0.1-25 |  | 0.0189 | 0.0535 | 0.0101 |  |
|  |  | 298.15 | 0.1-25 |  | 0.0184 | 0.0452 | 0.0184 |  |
|  |  | 308.15 | 0.1-25 |  | 0.0235 | 0.0414 | 0.0235 |  |
|  |  | 318.15 | 0.1-25 |  | 0.0097 | 0.0266 | -0.0028 |  |
| [P_666 14_]Cl | REF 67 | 283.15 | 0.1-45 | for  T = 333.15 K  and  p = 45 MPa  0.10 % | 0.0095 | 0.0159 | -0.0094 | Isothermal Compressibility from REF 66 |
|  |  | 293.15 | 0.1-45 |  | 0.0165 | 0.0364 | -0.0161 |  |
|  |  | 303.15 | 0.1-45 |  | 0.0236 | 0.0547 | -0.0236 |  |
|  |  | 313.15 | 0.1-45 |  | 0.0319 | 0.0756 | -0.0317 |  |
|  |  | 323.15 | 0.1-45 |  | 0.0364 | 0.0878 | -0.0364 |  |
|  |  | 333.15 | 0.1-45 |  | 0.0368 | 0.1021 | -0.0358 |  |
| [C_6_mim][PF_6_] | REF 12 | 293.15 | 0.1 | for  T = 472.3 K  and  p = 30 MPa  1.46 % | 0.0015 | 0.0015 | -0.0015 | Isothermal Compressibility calculated from Tait equation from REF 24 |
|  |  | 313.15 | 0.1 |  | 0.0047 | 0.0047 | 0.0047 |  |
|  |  | 333.15 | 0.1 |  | 0.0047 | 0.0047 | -0.0047 |  |
|  |  | 353.15 | 0.1 |  | 0.0016 | 0.0016 | 0.0016 |  |
|  |  | 312.9 | 10-200 |  | 0.1198 | 0.3039 | -0.1183 |  |
|  |  | 332.6 | 10-200 |  | 0.1830 | 0.4586 | -0.1758 |  |
|  |  | 352.7 | 10-200 |  | 0.2298 | 0.5762 | -0.2077 |  |
|  |  | 372.9 | 10-200 |  | 0.2478 | 0.7058 | -0.1917 |  |
|  |  | 393 | 10-200 |  | 0.2684 | 0.6952 | -0.1031 |  |
|  |  | 412.9 | 10-200 |  | 0.3392 | 0.6984 | 0.0128 |  |
|  |  | 432.8 | 10-200 |  | 0.4430 | 0.7338 | 0.1736 |  |
|  |  | 452.5 | 10-200 |  | 0.5917 | 1.0231 | 0.3898 |  |
|  |  | 472.3 | 10-200 |  | 0.7947 | 1.4371 | 0.6363 |  |
| [C_6_mim][PF_6_] | REF 24 | 293.15 | 0.1-10 | for  T = 353.15 K  and  p = 10 MPa  0.04 % | 0.0117 | 0.0332 | 0.0117 | Isothermal Compressibility calculated from Tait equation from REF 24 |
|  |  | 303.15 | 0.1-10 |  | 0.0082 | 0.0242 | 0.0022 |  |
|  |  | 313.15 | 0.1-10 |  | 0.0047 | 0.0162 | 0.0037 |  |
|  |  | 323.15 | 0.1-10 |  | 0.0043 | 0.0091 | 0.0028 |  |
|  |  | 333.15 | 0.1-10 |  | 0.0031 | 0.0064 | 0.0030 |  |
|  |  | 343.15 | 0.1-10 |  | 0.0062 | 0.0302 | 0.0033 |  |
|  |  | 353.15 | 0.1-10 |  | 0.0231 | 0.0432 | 0.0231 |  |
|  |  | 363.15 | 0.1-10 |  | 0.0050 | 0.0180 | 0.0031 |  |
|  |  | 373.15 | 0.1-10 |  | 0.0119 | 0.0166 | -0.0088 |  |
|  |  | 383.15 | 0.1-10 |  | 0.0115 | 0.0186 | -0.0077 |  |
|  |  | 393.15 | 0.1-10 |  | 0.0196 | 0.0350 | 0.0196 |  |
| [C_6_mim][PF_6_] | REF 49 | 293.15 | 0.1-20 | for  T = 293.15 K  and  p = 20 MPa  0.11 % | 0.0540 | 0.1056 | -0.0540 | Isothermal Compressibility calculated from Tait equation from REF 24 |
|  |  | 313.15 | 0.1-20 |  | 0.0177 | 0.0362 | -0.0029 |  |
|  |  | 333.15 | 0.1-20 |  | 0.0260 | 0.0487 | -0.0065 |  |
|  |  | 353.15 | 0.1-20 |  | 0.0462 | 0.0722 | 0.0462 |  |
| [C_6_mim][PF_6_] | REF 50 | 273.15 | 0.1 | for  T = 348.15 K  and  p = 238.5 MPa  0.37 % | 0.0012 | 0.0012 | 0.0012 | Isothermal Compressibility calculated from Tait equation from REF 24 |
|  |  | 278.15 | 0.1 |  | 0.0002 | 0.0002 | 0.0002 |  |
|  |  | 283.15 | 0.1 |  | 0.0011 | 0.0011 | -0.0011 |  |
|  |  | 288.15 | 0.1 |  | 0.0019 | 0.0019 | -0.0019 |  |
|  |  | 293.15 | 0.1 |  | 0.0021 | 0.0021 | -0.0021 |  |
|  |  | 298.15 | 0.1-100.1 |  | 0.0912 | 0.2238 | 0.0909 |  |
|  |  | 303.15 | 0.1 |  | 0.0006 | 0.0006 | 0.0006 |  |
|  |  | 313.15 | 0.1-150 |  | 0.1125 | 0.3324 | 0.1125 |  |
|  |  | 323.15 | 0.1-174.8 |  | 0.1066 | 0.3370 | 0.1049 |  |
|  |  | 333.15 | 0.1-200.1 |  | 0.1222 | 0.3522 | 0.1212 |  |
|  |  | 343.15 | 0.1 |  | 0.0031 | 0.0031 | -0.0031 |  |
|  |  | 348.15 | 0.1-238.5 |  | 0.1108 | 0.3679 | 0.1096 |  |
|  |  | 353.15 | 0.1 |  | 0.0011 | 0.0011 | -0.0011 |  |
|  |  | 363.15 | 0.1 |  | 0.0033 | 0.0033 | 0.0033 |  |
| [C_6_mim][PF_6_] | REF 79 | 294.1 | 0.1-20 | for  T = 294.1 K  and  p = 20 MPa  0.10 % | 0.0470 | 0.0982 | -0.0470 | Isothermal Compressibility calculated from Tait equation from REF 24 |
|  |  | 315.1 | 0.1-20 |  | 0.0249 | 0.0548 | -0.0249 |  |
|  |  | 335.2 | 0.1-20 |  | 0.0042 | 0.0105 | 0.0042 |  |
| [C_8_mim][PF_6_] | REF 12 | 293.15 | 0.1 | for  T = 312.8 K  and  p = 200 MPa  0.91 % | 0.0024 | 0.0024 | -0.0024 | Isothermal Compressibility from REF 19 |
|  |  | 313.15 | 0.1 |  | 0.0073 | 0.0073 | 0.0073 |  |
|  |  | 333.15 | 0.1 |  | 0.0074 | 0.0074 | -0.0074 |  |
|  |  | 353.15 | 0.1 |  | 0.0025 | 0.0025 | 0.0025 |  |
|  |  | 312.8 | 10-200 |  | 0.4329 | 0.9020 | 0.4329 |  |
|  |  | 332.6 | 10-200 |  | 0.2909 | 0.6936 | 0.2909 |  |
|  |  | 352.6 | 10-200 |  | 0.1422 | 0.4272 | 0.1336 |  |
|  |  | 372.8 | 10-200 |  | 0.0809 | 0.1810 | -0.0180 |  |
|  |  | 392.8 | 10-200 |  | 0.1734 | 0.2741 | -0.1480 |  |
|  |  | 413 | 10-200 |  | 0.2834 | 0.4023 | -0.2455 |  |
|  |  | 432.7 | 10-200 |  | 0.3899 | 0.5572 | -0.3382 |  |
|  |  | 452.5 | 10-200 |  | 0.4710 | 0.6959 | -0.3743 |  |
|  |  | 472.3 | 10-200 |  | 0.5619 | 0.8623 | -0.4113 |  |
| [C_8_mim][PF_6_] | REF 19 | 298.2 | 0.1-204.18 | for  T = 298.2 K  and  p = 173.16 MPa  1.22 % | 0.5549 | 1.2027 | 0.5513 | Isothermal Compressibility from REF 19 |
|  |  | 303.2 | 0.1 |  | 0.0200 | 0.0200 | 0.0200 |  |
|  |  | 313.2 | 0.1 |  | 0.0042 | 0.0042 | -0.0042 |  |
|  |  | 323.2 | 0.1-202.81 |  | 0.4068 | 0.8217 | 0.4068 |  |
|  |  | 333.2 | 0.1 |  | 0.0149 | 0.0149 | -0.0149 |  |
|  |  | 343.2 | 0.1 |  | 0.0074 | 0.0074 | 0.0074 |  |
| [C_8_mim][PF_6_] | REF 24 | 293.15 | 0.1-10 | for  T = 393.15 K  and  p = 10 MPa  0.39 % | 0.0269 | 0.0585 | 0.0269 | Isothermal Compressibility from REF 19 |
|  |  | 303.15 | 0.1-10 |  | 0.0062 | 0.0219 | 0.0044 |  |
|  |  | 313.15 | 0.1-10 |  | 0.0039 | 0.0127 | 0.0001 |  |
|  |  | 323.15 | 0.1-10 |  | 0.0465 | 0.0497 | -0.0465 |  |
|  |  | 333.15 | 0.1-10 |  | 0.0136 | 0.0186 | -0.0136 |  |
|  |  | 343.15 | 0.1-10 |  | 0.0104 | 0.0200 | 0.0018 |  |
|  |  | 353.15 | 0.1-10 |  | 0.0232 | 0.0564 | -0.0209 |  |
|  |  | 363.15 | 0.1-10 |  | 0.0208 | 0.0568 | -0.0151 |  |
|  |  | 373.15 | 0.1-10 |  | 0.0247 | 0.0705 | -0.0215 |  |
|  |  | 383.15 | 0.1-10 |  | 0.0572 | 0.1146 | -0.0572 |  |
|  |  | 393.15 | 0.1-10 |  | 0.0579 | 0.1385 | -0.0579 |  |
| [C_8_mim][PF_6_] | REF 46 | 273.15 | 0.1 | for  T = 308.15 K  and  p = 74.9 MPa  0.38 % | 0.0043 | 0.0055 | 0.0043 | Isothermal Compressibility from REF 19 |
|  |  | 278.15 | 0.1 |  | 0.0031 | 0.0051 | 0.0031 |  |
|  |  | 283.15 | 0.1 |  | 0.0013 | 0.0021 | -0.0013 |  |
|  |  | 288.15 | 0.1 |  | 0.0017 | 0.0017 | -0.0017 |  |
|  |  | 293.15 | 0.1 |  | 0.0041 | 0.0049 | -0.0041 |  |
|  |  | 298.15 | 0.1-39.9 |  | 0.0738 | 0.2377 | 0.0724 |  |
|  |  | 303.15 | 0.1 |  | 0.0034 | 0.0038 | -0.0034 |  |
|  |  | 308.15 | 0.1-74.9 |  | 0.1324 | 0.3782 | 0.1324 |  |
|  |  | 313.15 | 0.1 |  | 0.0020 | 0.0037 | 0.0020 |  |
|  |  | 323.15 | 0.1-99.7 |  | 0.1352 | 0.3295 | 0.1352 |  |
|  |  | 333.15 | 0.1-125.2 |  | 0.0823 | 0.2681 | 0.0820 |  |
|  |  | 343.15 | 0.1-175.9 |  | 0.0544 | 0.2186 | 0.0399 |  |
|  |  | 353.15 | 0.1 |  | 0.0025 | 0.0029 | -0.0025 |  |
|  |  | 363.15 | 0.1 |  | 0.0010 | 0.0010 | 0.0010 |  |
| [C_8_mim][PF_6_] | REF 47 | 298.15 | 0.1-98.1 | for  T = 323.15 K  and  p = 196.1 MPa  0.82 % | 0.3067 | 0.6441 | 0.3060 | Isothermal Compressibility from REF 19 |
|  |  | 323.15 | 0.1-196.1 |  | 0.3568 | 0.8136 | 0.3568 |  |
|  |  | 348.15 | 195.8 |  | 0.0505 | 0.1429 | 0.0059 |  |
| [C_8_mim][PF_6_] | REF 49 | 293.15 | 0.1-20 | for  T = 333.15 K  and  p = 15 MPa  0.12 % | 0.0810 | 0.1088 | -0.0810 | Isothermal Compressibility from REF 19 |
|  |  | 313.15 | 0.1-20 |  | 0.0158 | 0.0245 | 0.0158 |  |
|  |  | 333.15 | 0.1-20 |  | 0.0825 | 0.1184 | -0.0825 |  |
|  |  | 353.15 | 0.1-20 |  | 0.0670 | 0.0878 | 0.0670 |  |
| [C_8_mim][PF_6_] | REF 79 | 295.1 | 0.1-20 | for  T = 295.1 K  and  p = 20 MPa  0.10 % | 0.0638 | 0.1036 | -0.0638 | Isothermal Compressibility from REF 19 |
|  |  | 315.1 | 0.1-20 |  | 0.0090 | 0.0205 | -0.0090 |  |
|  |  | 335.2 | 0.1-20 |  | 0.0081 | 0.0183 | -0.0081 |  |
| [C_6_mim][OTf] | REF 14 | 303.139 | 0.1038 | for  T = 351.54 K  and  p = 29.91 MPa  1.21 % | 0.9099 | 0.9099 | -0.9099 | Density and Isothermal Compressibility calculated using the GCM method proposed in REF 53 |
|  |  | 303.148 | 9.9485 |  | 0.9996 | 0.9996 | -0.9996 |  |
|  |  | 303.149 | 29.54 |  | 0.9534 | 0.9534 | -0.9534 |  |
|  |  | 303.151 | 50.077 |  | 0.9401 | 0.9401 | -0.9401 |  |
|  |  | 303.154 | 18.778 |  | 0.9551 | 1.0020 | -0.9551 |  |
|  |  | 303.156 | 19.876 |  | 0.9038 | 0.9038 | -0.9038 |  |
|  |  | 303.158 | 40.749 |  | 0.9391 | 0.9391 | -0.9391 |  |
|  |  | 303.16 | 30.131 |  | 0.9260 | 0.9260 | -0.9260 |  |
|  |  | 303.163 | 40.525 |  | 0.9490 | 0.9490 | -0.9490 |  |
|  |  | 303.928 | 1.1695 |  | 1.0038 | 1.0038 | -1.0038 |  |
|  |  | 311.103 | 19.363 |  | 0.9474 | 0.9474 | -0.9474 |  |
|  |  | 311.104 | 50.109 |  | 0.9747 | 0.9747 | -0.9747 |  |
|  |  | 311.402 | 59.971 |  | 0.9761 | 0.9761 | -0.9761 |  |
|  |  | 311.642 | 29.371 |  | 0.9503 | 0.9503 | -0.9503 |  |
|  |  | 312.124 | 10.064 |  | 1.0482 | 1.0482 | -1.0482 |  |
|  |  | 312.129 | 19.984 |  | 1.0419 | 1.0419 | -1.0419 |  |
|  |  | 312.35 | 40.711 |  | 0.9708 | 0.9708 | -0.9708 |  |
|  |  | 312.667 | 1.1378 |  | 1.0464 | 1.0464 | -1.0464 |  |
|  |  | 320.012 | 60.231 |  | 1.0238 | 1.0238 | -1.0238 |  |
|  |  | 320.693 | 29.908 |  | 0.9928 | 0.9928 | -0.9928 |  |
|  |  | 321.26 | 40.148 |  | 0.9933 | 0.9933 | -0.9933 |  |
|  |  | 321.321 | 10.225 |  | 1.0917 | 1.0917 | -1.0917 |  |
|  |  | 321.343 | 19.896 |  | 1.0976 | 1.0976 | -1.0976 |  |
|  |  | 321.701 | 51.336 |  | 1.0132 | 1.0132 | -1.0132 |  |
|  |  | 322.861 | 1.202 |  | 1.0955 | 1.0955 | -1.0955 |  |
|  |  | 330.072 | 40.113 |  | 1.0321 | 1.0321 | -1.0321 |  |
|  |  | 330.332 | 60.665 |  | 1.0651 | 1.0651 | -1.0651 |  |
|  |  | 330.451 | 29.706 |  | 1.1319 | 1.1319 | -1.1319 |  |
|  |  | 330.662 | 50.504 |  | 1.0339 | 1.0339 | -1.0339 |  |
|  |  | 331.614 | 20.974 |  | 1.1386 | 1.1386 | -1.1386 |  |
|  |  | 331.615 | 10.028 |  | 1.1421 | 1.1421 | -1.1421 |  |
|  |  | 332.502 | 1.1534 |  | 1.1345 | 1.1345 | -1.1345 |  |
|  |  | 339.162 | 49.826 |  | 1.0711 | 1.0711 | -1.0711 |  |
|  |  | 339.455 | 59.226 |  | 1.1081 | 1.1081 | -1.1081 |  |
|  |  | 340.067 | 39.808 |  | 1.1713 | 1.1713 | -1.1713 |  |
|  |  | 340.306 | 30.074 |  | 1.1675 | 1.1675 | -1.1675 |  |
|  |  | 341.511 | 9.9335 |  | 1.1812 | 1.1812 | -1.1812 |  |
|  |  | 341.516 | 20.038 |  | 1.1837 | 1.1837 | -1.1837 |  |
|  |  | 342.34 | 0.9772 |  | 1.1834 | 1.1834 | -1.1834 |  |
|  |  | 348.25 | 58.008 |  | 1.1717 | 1.1717 | -1.1717 |  |
|  |  | 349.94 | 49.966 |  | 1.2127 | 1.2127 | -1.2127 |  |
|  |  | 350.06 | 39.798 |  | 1.2165 | 1.2165 | -1.2165 |  |
|  |  | 351.537 | 29.91 |  | 1.2282 | 1.2282 | -1.2282 |  |
|  |  | 351.766 | 10.055 |  | 1.2188 | 1.2188 | -1.2188 |  |
|  |  | 351.769 | 19.878 |  | 1.2269 | 1.2269 | -1.2269 |  |
| [C_8_mim][NTf_2_] | REF 11 | 293.15 | 0.1-30 | for  T = 393.15 K  and  p = 30 MPa  0.23 % | 0.0955 | 0.1686 | -0.0955 | Isothermal Compressibility from REF 19 |
|  |  | 303.15 | 0.1-30 |  | 0.0476 | 0.0647 | 0.0347 |  |
|  |  | 313.15 | 0.1-30 |  | 0.0416 | 0.0660 | 0.0251 |  |
|  |  | 323.15 | 0.1-30 |  | 0.0264 | 0.1026 | -0.0087 |  |
|  |  | 333.15 | 0.1-30 |  | 0.0318 | 0.1435 | -0.0299 |  |
|  |  | 343.15 | 0.1-30 |  | 0.0693 | 0.2042 | -0.0693 |  |
|  |  | 353.15 | 0.1-30 |  | 0.0805 | 0.2079 | -0.0805 |  |
|  |  | 393.15 | 0.1-30 |  | 0.0537 | 0.2270 | -0.0356 |  |
| [C_8_mim][NTf_2_] | REF 64 | 293.22 | 0.103-2.5 | for  T = 343.15 K  and  p = 25 MPa  0.29 % | 0.2054 | 0.2120 | 0.2054 | Density (p = 0.1 MPa), Isothermal Compressibility from REF 11 |
|  |  | 293.23 | 0.247-25 |  | 0.1736 | 0.2110 | 0.1736 |  |
|  |  | 303.19 | 0.101-0.25 |  | 0.0893 | 0.0895 | 0.0893 |  |
|  |  | 303.21 | 0.5 |  | 0.0862 | 0.0862 | 0.0862 |  |
|  |  | 303.25 | 1-15 |  | 0.0640 | 0.0871 | 0.0640 |  |
|  |  | 303.3 | 20-25 |  | 0.0313 | 0.0349 | 0.0313 |  |
|  |  | 313.25 | 0.102 |  | 0.0091 | 0.0091 | -0.0091 |  |
|  |  | 313.29 | 0.246 |  | 0.0041 | 0.0041 | -0.0041 |  |
|  |  | 313.33 | 0.499 |  | 0.0005 | 0.0005 | -0.0005 |  |
|  |  | 313.37 | 1 |  | 0.0059 | 0.0059 | -0.0059 |  |
|  |  | 313.38 | 2.5 |  | 0.0067 | 0.0067 | -0.0067 |  |
|  |  | 313.41 | 5 |  | 0.0232 | 0.0232 | -0.0232 |  |
|  |  | 313.43 | 10-20 |  | 0.0516 | 0.0676 | -0.0516 |  |
|  |  | 313.44 | 25 |  | 0.0746 | 0.0746 | -0.0746 |  |
|  |  | 323.1 | 5-20 |  | 0.1276 | 0.1501 | -0.1276 |  |
|  |  | 323.11 | 2.5-25 |  | 0.1262 | 0.1565 | -0.1262 |  |
|  |  | 323.12 | 0.502-1 |  | 0.0838 | 0.0845 | -0.0838 |  |
|  |  | 323.13 | 0.103-0.249 |  | 0.0838 | 0.0843 | -0.0838 |  |
|  |  | 333.11 | 0.497 |  | 0.1721 | 0.1721 | -0.1721 |  |
|  |  | 333.12 | 0.103-25 |  | 0.1915 | 0.2372 | -0.1915 |  |
|  |  | 333.13 | 0.25-20 |  | 0.2038 | 0.2255 | -0.2038 |  |
|  |  | 343.12 | 0.101-1 |  | 0.2257 | 0.2279 | -0.2257 |  |
|  |  | 343.13 | 0.249-25 |  | 0.2531 | 0.2920 | -0.2531 |  |
| [C_8_mim][BF_4_] | REF 13 | 283.15 | 0.1-60 | for  T = 323.15 K  and  p = 60 MPa  0.16 % | 0.0242 | 0.1217 | -0.0217 | Isothermal Compressibility from REF 19 |
|  |  | 288.15 | 0.1-60 |  | 0.0182 | 0.0948 | -0.0131 |  |
|  |  | 293.15 | 0.1-60 |  | 0.0153 | 0.0609 | 0.0001 |  |
|  |  | 298.15 | 0.1-60 |  | 0.0177 | 0.0324 | 0.0112 |  |
|  |  | 303.15 | 0.1-60 |  | 0.0246 | 0.0452 | 0.0238 |  |
|  |  | 308.15 | 0.1-60 |  | 0.0382 | 0.0701 | 0.0382 |  |
|  |  | 313.15 | 0.1-60 |  | 0.0524 | 0.0922 | 0.0524 |  |
|  |  | 318.15 | 0.1-60 |  | 0.0665 | 0.1209 | 0.0665 |  |
|  |  | 323.15 | 0.1-60 |  | 0.0861 | 0.1567 | 0.0860 |  |
| [C_8_mim][BF_4_] | REF 19 | 298.2 | 0.1-202.94 | for  T = 298.2 K  and  p = 202.81 MPa  0.54 % | 0.1930 | 0.5428 | -0.1539 | Isothermal Compressibility from REF 19 |
|  |  | 303.2 | 0.1 |  | 0.0317 | 0.0317 | -0.0317 |  |
|  |  | 313.2 | 0.1 |  | 0.0417 | 0.0417 | -0.0417 |  |
|  |  | 323.2 | 206.94 |  | 0.0862 | 0.1552 | -0.0077 |  |
|  |  | 333.2 | 0.1 |  | 0.0012 | 0.0012 | 0.0012 |  |
|  |  | 343.2 | 0.1 |  | 0.0112 | 0.0112 | -0.0112 |  |
| [C_8_mim][BF_4_] | REF 24 | 293.15 | 0.1-10 | for  T = 293.15 K  and  p = 10 MPa  0.38 % | 0.0200 | 0.0606 | 0.0190 | Isothermal Compressibility from REF 19 |
|  |  | 303.15 | 0.1-10 |  | 0.0213 | 0.0624 | 0.0201 |  |
|  |  | 313.15 | 0.1-10 |  | 0.0280 | 0.0739 | 0.0280 |  |
|  |  | 323.15 | 0.1-10 |  | 0.0389 | 0.0957 | 0.0389 |  |
|  |  | 333.15 | 0.1-10 |  | 0.0518 | 0.1282 | 0.0518 |  |
|  |  | 343.15 | 0.1-10 |  | 0.0496 | 0.1442 | 0.0494 |  |
|  |  | 353.15 | 0.1-10 |  | 0.0732 | 0.1901 | 0.0732 |  |
|  |  | 363.15 | 0.1-10 |  | 0.0667 | 0.2012 | 0.0618 |  |
|  |  | 373.15 | 0.1-10 |  | 0.0868 | 0.2431 | 0.0852 |  |
|  |  | 383.15 | 0.1-10 |  | 0.1062 | 0.2880 | 0.1047 |  |
|  |  | 393.15 | 0.1-10 |  | 0.1518 | 0.3742 | 0.1518 |  |
| [C_8_mim][BF_4_] | REF 46 | 273.15 | 0.1 | for  T = 323.15 K  and  p = 199.8 MPa  1.14 % | 0.0035 | 0.0035 | 0.0035 | Isothermal Compressibility from REF 19 |
|  |  | 278.15 | 0.1 |  | 0.0017 | 0.0017 | 0.0017 |  |
|  |  | 283.15 | 0.1 |  | 0.0002 | 0.0002 | 0.0002 |  |
|  |  | 288.15 | 0.1 |  | 0.0010 | 0.0010 | -0.0010 |  |
|  |  | 293.15 | 0.1 |  | 0.0029 | 0.0029 | -0.0029 |  |
|  |  | 298.15 | 0.1-117.5 |  | 0.0859 | 0.1905 | -0.0859 |  |
|  |  | 303.15 | 0.1 |  | 0.0038 | 0.0038 | -0.0038 |  |
|  |  | 308.15 | 0.1-200.1 |  | 0.0711 | 0.2345 | -0.0683 |  |
|  |  | 313.15 | 0.1 |  | 0.0001 | 0.0001 | 0.0001 |  |
|  |  | 323.15 | 0.1-199.8 |  | 0.3266 | 1.1571 | -0.2812 |  |
|  |  | 333.15 | 0.1-200 |  | 0.0337 | 0.0590 | 0.0002 |  |
|  |  | 343.15 | 0.1 |  | 0.0030 | 0.0030 | 0.0030 |  |
|  |  | 348.15 | 0.1-224.2 |  | 0.0640 | 0.1296 | 0.0639 |  |
|  |  | 353.15 | 0.1 |  | 0.0001 | 0.0001 | -0.0001 |  |
|  |  | 363.15 | 0.1 |  | 0.0038 | 0.0038 | -0.0038 |  |
| [C_8_mim][BF_4_] | REF 47 | 298.15 | 0.1-196.1 | for  T = 298.15 K  and  p = 196.1 MPa  0.28 % | 0.1375 | 0.2803 | -0.1375 | Isothermal Compressibility from REF 19 |
|  |  | 323.15 | 0.1-196.1 |  | 0.0607 | 0.1192 | -0.0508 |  |
|  |  | 348.15 | 0.1-196.1 |  | 0.0747 | 0.1085 | 0.0747 |  |
| [C_4_mim][PF_6_] | REF 19 | 298.2 | 0.1-202.11 | for  T = 298.2 K  and  p = 172.47 MPa  0.37 % | 0.1923 | 0.3741 | -0.1294 | Isothermal Compressibility from REF 19 |
|  |  | 303.2 | 0.1 |  | 0.0032 | 0.0032 | 0.0032 |  |
|  |  | 313.2 | 0.1 |  | 0.0288 | 0.0288 | -0.0288 |  |
|  |  | 323.2 | 0.1-200.74 |  | 0.1248 | 0.2518 | -0.0237 |  |
|  |  | 333.2 | 0.1 |  | 0.0120 | 0.0120 | -0.0120 |  |
|  |  | 343.2 | 0.1 |  | 0.0002 | 0.0002 | 0.0002 |  |
| [C_4_mim][PF_6_] | REF 22 | 298.15 | 0.1-100 | for  T = 298.15 K  and  p = 100 MPa  0.09 % | 0.0284 | 0.0925 | -0.0284 | Isothermal Compressibility Calculated from Speed of Sound Data from REF 22 |
|  |  | 303.15 | 0.1-100 |  | 0.0264 | 0.0868 | -0.0264 |  |
|  |  | 308.15 | 0.1-100 |  | 0.0246 | 0.0814 | -0.0245 |  |
|  |  | 313.15 | 0.1-100 |  | 0.0229 | 0.0762 | -0.0228 |  |
|  |  | 318.15 | 0.1-100 |  | 0.0211 | 0.0714 | -0.0211 |  |
|  |  | 323.15 | 0.1-100 |  | 0.0196 | 0.0677 | -0.0195 |  |
| [C_4_mim][PF_6_] | REF 23 | 293.15 | 0.1-20 | for  T = 293.15 K  and  p = 20 MPa  0.13 % | 0.0796 | 0.1311 | 0.07961 | Isothermal Compressibility Calculated from Speed of Sound Data from REF 22 |
|  |  | 313.15 | 0.1-20 |  | 0.0381 | 0.0797 | 0.01736 |  |
|  |  | 333.15 | 0.1-20 |  | 0.0283 | 0.0322 | 0.02828 |  |
|  |  | 353.15 | 0.1-20 |  | 0.0422 | 0.1235 | 0.03919 |  |
| [C_4_mim][PF_6_] | REF 25 | 298.15 | 1.22-39.51 | for  T = 373.15 K  and  p = 0.87 MPa  0.49 % | 0.0614 | 0.0883 | 0.0614 | Density (p = 0.1 MPa) from REF 22, Isothermal Compressibility Calculated from Speed of Sound Data from REF 22 |
|  |  | 323.15 | 1.01-39.93 |  | 0.2459 | 0.3195 | 0.2459 |  |
|  |  | 348.15 | 1.36-39.67 |  | 0.3183 | 0.4105 | 0.3183 |  |
|  |  | 373.15 | 0.87-39.98 |  | 0.3781 | 0.4879 | 0.3781 |  |
|  |  | 398.15 | 0.73-40.07 |  | 0.3589 | 0.4724 | 0.3589 |  |
| [C_4_mim][PF_6_] | REF 26 | 293.48 | 1 | for  T = 414.92 K  and  p = 40 MPa  0.61 % | 0.0219 | 0.0219 | 0.0219 | Isothermal Compressibility Calculated from Speed of Sound Data from REF 22 |
|  |  | 293.49 | 0.1-20 |  | 0.0809 | 0.1535 | 0.0726 |  |
|  |  | 293.5 | 10-40 |  | 0.2015 | 0.3208 | 0.2015 |  |
|  |  | 293.51 | 5-30 |  | 0.1436 | 0.2363 | 0.1436 |  |
|  |  | 302.73 | 0.1 |  | 0.0032 | 0.0032 | -0.0032 |  |
|  |  | 312.86 | 0.1 |  | 0.0005 | 0.0005 | 0.0005 |  |
|  |  | 322.28 | 0.1 |  | 0.0129 | 0.0129 | 0.0129 |  |
|  |  | 322.3 | 10-20 |  | 0.1165 | 0.1590 | 0.1165 |  |
|  |  | 322.31 | 1-40 |  | 0.1787 | 0.3574 | 0.1787 |  |
|  |  | 322.33 | 5-30 |  | 0.1457 | 0.2613 | 0.1457 |  |
|  |  | 332.73 | 0.1 |  | 0.0008 | 0.0008 | 0.0008 |  |
|  |  | 342.5 | 0.1 |  | 0.0178 | 0.0178 | 0.0178 |  |
|  |  | 352.29 | 0.1 |  | 0.0022 | 0.0022 | -0.0022 |  |
|  |  | 352.3 | 40 |  | 0.4127 | 0.4127 | 0.4127 |  |
|  |  | 352.31 | 1-20 |  | 0.0884 | 0.1776 | 0.0810 |  |
|  |  | 352.32 | 5 |  | 0.0286 | 0.0286 | 0.0286 |  |
|  |  | 352.34 | 30 |  | 0.2948 | 0.2948 | 0.2948 |  |
|  |  | 373.3 | 5 |  | 0.0350 | 0.0350 | 0.0350 |  |
|  |  | 373.31 | 10 |  | 0.0805 | 0.0805 | 0.0805 |  |
|  |  | 373.32 | 30 |  | 0.3313 | 0.3313 | 0.3313 |  |
|  |  | 373.33 | 0.1-40 |  | 0.1656 | 0.4641 | 0.1439 |  |
|  |  | 373.34 | 20 |  | 0.2074 | 0.2074 | 0.2074 |  |
|  |  | 391.27 | 10 |  | 0.1098 | 0.1098 | 0.1098 |  |
|  |  | 391.28 | 30 |  | 0.3763 | 0.3763 | 0.3763 |  |
|  |  | 391.3 | 0.1-5 |  | 0.0256 | 0.0419 | 0.0163 |  |
|  |  | 391.31 | 20-40 |  | 0.3756 | 0.5221 | 0.3756 |  |
|  |  | 391.32 | 1 |  | 0.0096 | 0.0096 | 0.0096 |  |
|  |  | 414.91 | 10 |  | 0.1390 | 0.1390 | 0.1390 |  |
|  |  | 414.92 | 20-40 |  | 0.4438 | 0.6043 | 0.4438 |  |
|  |  | 414.93 | 0.1-30 |  | 0.1780 | 0.4426 | 0.1780 |  |
|  |  | 414.94 | 1 |  | 0.0270 | 0.0270 | 0.0270 |  |
| [C_4_mim][PF_6_] | REF 29 | 312.8 | 0.1-200 | for  T = 472.3 K  and  p = 200 MPa  0.66 % | 0.0768 | 0.2420 | -0.0761 | Isothermal Compressibility Calculated from Speed of Sound Data from REF 22 |
|  |  | 332.6 | 0.1-200 |  | 0.0612 | 0.1103 | 0.0409 |  |
|  |  | 353.2 | 0.1-200 |  | 0.1327 | 0.1850 | 0.1327 |  |
|  |  | 372.7 | 0.1-200 |  | 0.2519 | 0.3005 | 0.2519 |  |
|  |  | 392.8 | 0.1-200 |  | 0.1328 | 0.2110 | 0.1127 |  |
|  |  | 412.8 | 0.1-200 |  | 0.2010 | 0.3215 | 0.1929 |  |
|  |  | 432.6 | 0.1-200 |  | 0.2455 | 0.4255 | 0.2426 |  |
|  |  | 452.5 | 0.1-200 |  | 0.2938 | 0.5364 | 0.2938 |  |
|  |  | 472.3 | 0.1-200 |  | 0.3288 | 0.6582 | 0.3288 |  |
| [C_4_mim][PF_6_] | REF 79 | 294.9 | 0.1-20 | for  T = 294.9 K  and  p = 20 MPa  0.13 % | 0.0794 | 0.1305 | 0.0794 | Isothermal Compressibility Calculated from Speed of Sound Data from REF 22 |
|  |  | 315 | 0.1-20 |  | 0.0555 | 0.1063 | 0.0555 |  |
|  |  | 335.1 | 0.1-20 |  | 0.0560 | 0.0863 | 0.0560 |  |

$$RAAD/\%=\frac{100}{N}\sum_{i=1}^{N} \left|\frac{\rho_{i}^{calc}-\rho_{i}^{\exp}}{\rho_{i}^{calc}}\right|$$

$$D\max/\%=Max\left(100\left|\frac{\rho_{i}^{calc}-\rho_{i}^{\exp}}{\rho_{i}^{calc}}\right|\right)\text{ }$$

$$bias/\%=\frac{100}{N}\sum_{i=1}^{N} \frac{\rho_{i}^{calc}-\rho_{i}^{\exp}}{\rho_{i}^{calc}}$$

**Table S3. Comparison of calculated isothermal compressibility data (*κ*_T_ in Pa^-1^) of the [C_4_mim][PF_6_] at atmospheric pressure using the thermodynamics formalism (Eq. (3)) and data from Gomes de Azevedo *et al.* ^22^ and the GCM proposed by Jacquemin *et al.*^53^**

| *T* / K | 298.15 | 303.15 | 308.15 | 313.15 | 318.15 | 323.15 |
| --- | --- | --- | --- | --- | --- | --- |
| From Eq. (3) and REF [22] | 4.179·10^-10^ | 4.256·10^-10^ | 4.335·10^-10^ | 4.414·10^-10^ | 4.493·10^-10^ | 4.573·10^-10^ |
| Using the GCM from REF [53] | 4.248·10^-10^ | 4.324·10^-10^ | 4.400·10^-10^ | 4.476·10^-10^ | 4.552·10^-10^ | 4.629·10^-10^ |
| RAD /% | 1.66 | 1.60 | 1.50 | 1.40 | 1.32 | 1.21 |
| RAAD /% | 1.45 |  |  |  |  |  |

**Figure S1. Comparison between high-pressure density data (*ρ* in kg·m-3) of the [C4mim][PF6] reported by Gomes de Azevedo *et al.* 22 with those predicted by the FT-EOS using calculated isothermal compressibility data at atmospheric pressure determined by: a) the thermodynamics formalism (Eq. (3))22; and b) by the GCM proposed by Jacquemin *et al.*53**

| a) | b) |
| --- | --- |
| 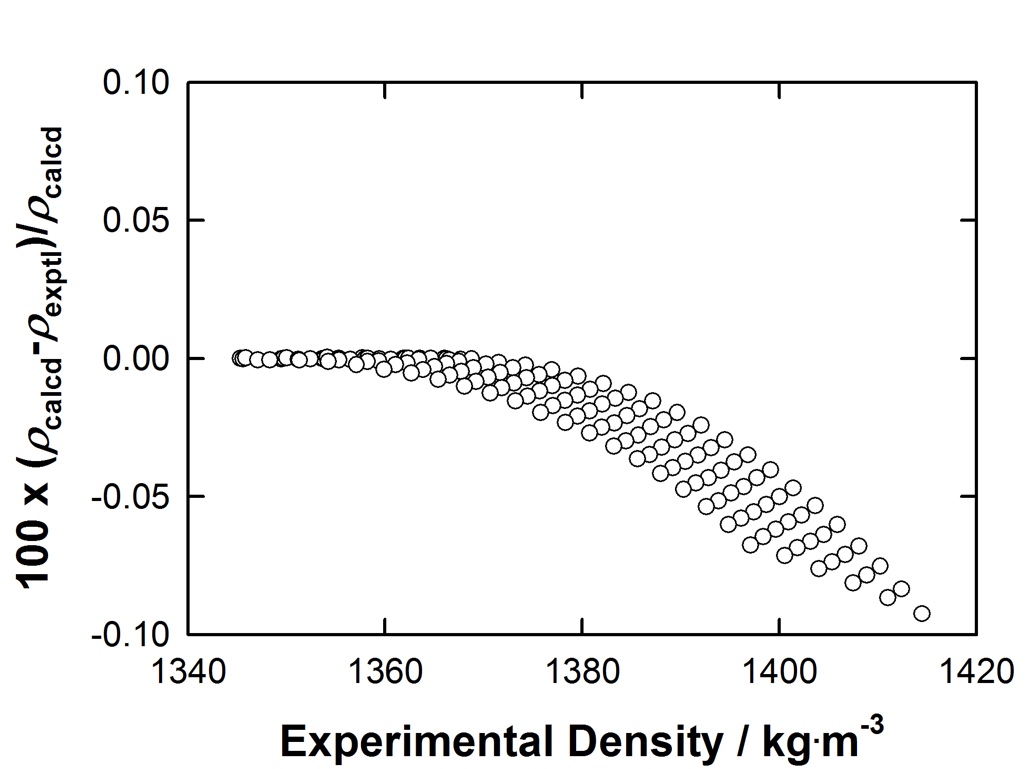 | 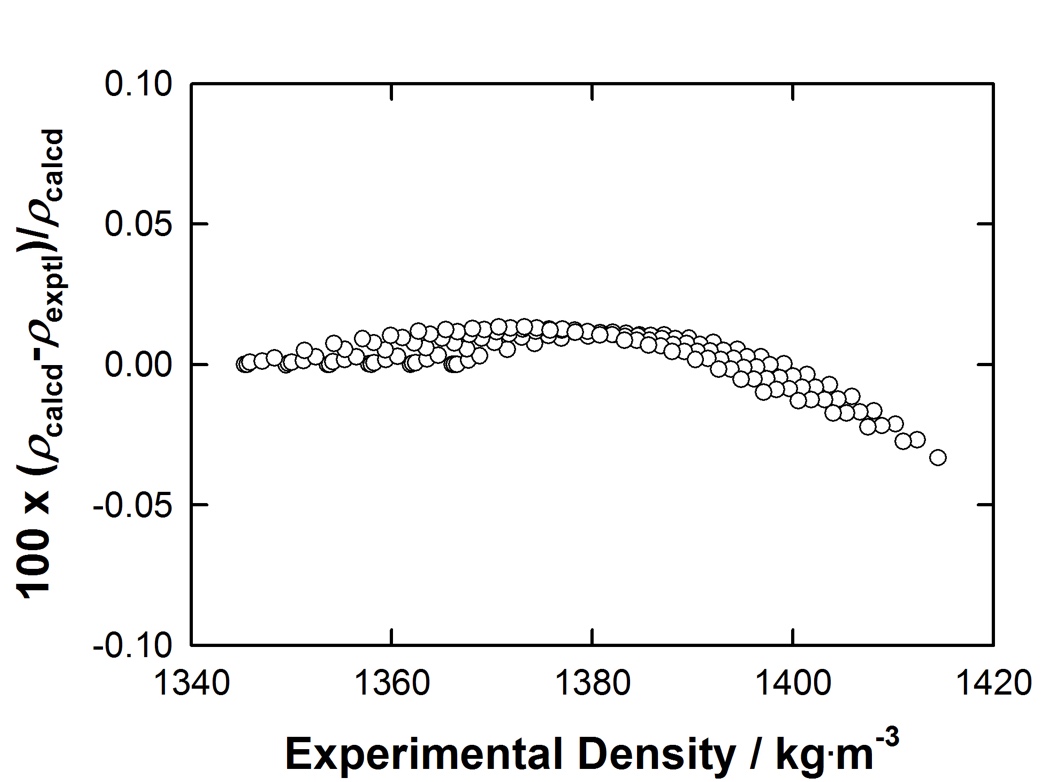 |

**References cited in Supplementary Information (*keeping their original numbers used in the main article*)**

6. Królikowska, M. &Hofman, T. Densities, Isobaric Expansivities and Isothermal Compressibilities of the Thiocyanate-Based Ionic Liquids at Temperatures (298.15-338.15 K) and Pressures up to 10 MPa. *Thermochim. Acta* **530**, 1-6 (2012).

7. Stevanovic, S., Podgoršek, A., Pádua, A. A. H. & Costa Gomes, M. F. Effect of Water on the Carbon Dioxide Absorption by 1-Alkyl-3-Methylimidazolium Acetate Ionic Liquids. *J. Phys. Chem. B* **116**, 14416-14425 (2012).

8. Machida, H., Ryosuke, T., Sato, Y. & Smith, R. L. J. Measurement and Correlation of High Pressure Densities of Ionic Liquids, 1-Ethyl-3-Methylimidazolium L-Lactate ([emim][Lactate]), 2-Hydroxyethyl-Trimethylammonium L-Lactate ([(C_2_H_4_OH)(CH_3_)_3_N][Lactate]), and 1-Butyl-3-Methylimidazolium Chloride ([bmim][Cl]). *J. Chem. Eng. Data* **56**, 923-928 (2011).

9. Klomfar, J., Součková, M. &Pátek, J. P–ρ–T Measurements for 1-Ethyl and 1-Butyl-3-Methylimidazolium Dicyanamides from Their Melting Temperature to 353 K and up to 60 MPa in Pressure. *J. Chem. Eng. Data* **57**, 1213-1221 (2012).

10. Freire, M. G. *et al*. Thermophysical Characterization of Ionic Liquids Able to Dissolve Biomass. *J. Chem. Eng. Data* **56**, 4813-4822 (2011).

11. Gardas, R. L. *et al*. PρT Measurements of Imidazolium-Based Ionic Liquids. *J. Chem. Eng. Data* **52**, 1881-1888 (2007).

12. Taguchi, R., Machida, H., Sato, Y. & Smith, R. L. High-Pressure Densities of 1-Alkyl-3-Methylimidazolium Hexafluorophosphates and 1-Alkyl-3-Methylimidazolium Tetrafluoroborates at Temperatures from (313 to 473) K and at Pressures up to 200 MPa. *J. Chem. Eng. Data* **54**, 22-27 (2009).

13. Sanmamed, Y. A. *et al*. Experimental Methodology for Precise Determination of Density of RTILs as a Function of Temperature and Pressure Using Vibrating Tube Densimeters. *J. Chem. Thermodyn.* **42**, 553-563 (2010).

14. Klomfar, J., Součková, M. &Pátek, J. P–ρ–T Measurements for 1-Alkyl-3-Methylimidazolium-Based Ionic Liquids with Tetrafluoroborate and a Trifluoromethanesulfonate Anion. *J. Chem. Eng. Data* **57**, 708-720 (2012).

15. Nieto de Castro *et al*. Studies on the Density, Heat Capacity, Surface Tension and Infinite Dilution Diffusion with the Ionic Liquids [C_4_mim][NTf_2_], [C_4_mim][dca], [C_2_mim][EtOSO_3_] and [Aliquat][dca]. *Fluid Phase Equilib.* **294**, 157-179 (2010).

16. Gołdon, A., Dąbrowska, K. &Hofman, T. Densities and Excess Volumes of the 1,3-Dimethylimidazolium Methylsulfate + Methanol System at Temperatures from (313.15 to 333.15) K and Pressures from (0.1 to 25) MPa. *J. Chem. Eng. Data* **52**, 1830-1837 (2007).

17. Aparicio, S., Alcalde, R., García, B. & Leal, J. M. High-Pressure Study of the Methylsulfate and Tosylate Imidazolium Ionic Liquids. *J. Phys. Chem. B* **113**, 5593-5606 (2009).

18. Tomé, L. I. N. *et al*. Measurements and Correlation of High-Pressure Densities of Imidazolium-Based Ionic Liquids. *J. Chem. Eng. Data* **53**, 1914-1921 (2008).

19. Gu, Z. &Brennecke, J. F. Volume Expansivities and Isothermal Compressibilities of Imidazolium and Pyridinium-Based Ionic Liquids. *J. Chem. Eng. Data* **47**,339-345(2002).

20. Tomida, D., Kenmochi, S., Qiao, K., Tsukada, T. & Yokoyama, C. Densities and Thermal Conductivities of N-AlkylpyridiniumTetrafluoroborates at High Pressure. *Fluid Phase Equilib.* **340**, 31-36 (2013).

21. Rebelo, L. P. N. *et al*. A Detailed Thermodynamic Analysis of [C_4_mim][BF_4_] + Water as a Case Study to Model Ionic Liquid Aqueous Solutions. *Green Chem.* **6**, 369-381 (2004).

22. Gomes de Azevedo, R. *et al*. Thermophysical and Thermodynamic Properties of 1-Butyl-3-Methylimidazolium Tetrafluoroborate and 1-Butyl-3-Methylimidazolium Hexafluorophosphate over an Extended Pressure Range. *J. Chem. Eng. Data* **50**, 997-1008 (2005).

23. Tomida, D., Kumagai, A., Qiao, K. & Yokoyama, C. Viscosity of [bmim][PF_6_] and [bmim][BF_4_] at High Pressure. *Int. J. Thermophys.* **27**, 39-47 (2006).

24. Gardas, R. L. *et al*. High-Pressure Densities and Derived Thermodynamic Properties of Imidazolium-Based Ionic Liquids. *J. Chem. Eng. Data* **52**, 80-88 (2007).

25. Tekin, A., Safarov, J., Shahverdiyev, A. & Hassel, E. (*P, , T*) Properties of 1-Butyl-3-Methylimidazolium Tetrafluoroborate and 1-Butyl-3-Methylimidazolium Hexafluorophosphate at *T* = (298.15 to 398.15) K and Pressures up to *p* = 40 MPa. *J. Mol. Liq*. **136**, 177-182 (2007).

26. Jacquemin, J., Husson, P., Mayer, V. &Cibulka, I. High-Pressure Volumetric Properties of Imidazolium-Based Ionic Liquids: Effect of the Anion. *J. Chem. Eng. Data* **52**, 2204-2211 (2007).

27. Harris, K. R., Kanakubo, M. & Woolf, L. A. Temperature and Pressure Dependence of the Viscosity of the Ionic Liquid 1-Butyl-3-Methylimidazolium Tetrafluoroborate: Viscosity and Density Relationships in Ionic Liquids. *J. Chem. Eng. Data* **52**, 2425-2430 (2007).

28. Abdulagatov, I. M., Tekin, A., Safarov, J., Shahverdiyev, A. & Hassel, E. Densities and Excess, Apparent, and Partial Molar Volumes of Binary Mixtures of BMIMBF_4_ + Ethanol as a Function of Temperature, Pressure, and Concentration. *Int. J. Thermophys.* **29**, 505-533 (2008).

29. Machida, H., Sato, Y. & Smith, R. L. Pressure-Volume-Temperature (PVT) Measurements of Ionic Liquids ([bmim^+^][PF_6_^-^], [bmim^+^][BF_4_^-^], [bmim^+^][OcSO_4_^-^]) and Analysis with the Sanchez-Lacombe Equation of State. *Fluid Phase Equilib.* **264**, 147-155(2008).

30. Han, C., Xia, S., Ma, P. & Zeng, F. Densities of Ionic Liquid [BMIM][BF_4_] + Ethanol, + Benzene, and + Acetonitrile at Different Temperature and Pressure. *J. Chem. Eng. Data* **54**, 2971-2977 (2009).

31. Rilo, E., Ferreira, A. G. M., Fonseca, I. M. A. &Cabeza, O. Densities and Derived Thermodynamic Properties of Ternary Mixtures 1-Butyl-3-Methyl-Imidazolium Tetrafluoroborate+ethanol+water at Seven Pressures and Two Temperatures. *Fluid Phase Equilib.* **296**, 53-59 (2010).

32. Currás, M. R. *et al*. Behavior of the Environmentally Compatible Absorbent 1-Butyl-3-Methylimidazolium Tetrafluoroborate with 2,2,2-Trifluoroethanol: Experimental Densities at High Pressures and Modeling of PVT and Phase Equilibria Behavior with PC-SAFT EoS. *Ind. Eng. Chem. Res.* **50**, 4065-4076 (2011).

33. Klomfar, J., Součková, M. &Pátek, J. Experimental P-ρ-T Data for 1-Butyl-3-Methylimidazolium Tetrafluoroborate at Temperatures from (240 to 353) K and at Pressures up to 60 MPa. *J. Chem. Eng. Data* **56**, 426-436 (2011).

34. Matkowska, D. &Hofman, T. High-Pressure Volumetric Properties of Ionic Liquids: 1-Butyl-3-Methylimidazolium Tetrafluoroborate, [C_4_mim][BF_4_], 1-Butyl-3-Methylimidazolium Methylsulfate [C_4_mim][MeSO_4_] and 1-Ethyl-3-Methylimidazolium Ethylsulfate, [C_2_mim][EtSO_4_]. *J. Mol. Liq.* **165**, 161-167 (2012).

35. Hofman, T., Gołdon, A., Nevines, A. &Letcher, T. M. Densities, Excess Volumes, Isobaric Expansivity, and Isothermal Compressibility of the (1-Ethyl-3-Methylimidazolium Ethylsulfate+methanol) System at Temperatures (283.15 to 333.15) K and Pressures from (0.1 to 35) MPa. *J. Chem. Thermodyn.* **40**, 580-591 (2008).

36. Matkowska, D., Gol̷don, A. &Hofman, T. Densities, Excess Volumes, Isobaric Expansivities, and Isothermal Compressibilities of the 1-Ethyl-3-Methylimidazolium Ethylsulfate + Ethanol System at Temperatures (283.15 to 343.15) K and Pressures from (0.1 to 35) MPa. *J. Chem. Eng. Data* **55**, 685-693 (2010).

37. Regueira, T., Lugo, L. &Fernández, J. High Pressure Volumetric Properties of 1-Ethyl-3-Methylimidazolium Ethylsulfate and 1-(2-Methoxyethyl)-1-Methyl-Pyrrolidinium Bis(trifluoromethylsulfonyl)imide. *J. Chem. Thermodyn.* **48**, 213-220 (2012).

38. Schmidt, H. *et al*. Experimental Study of the Density and Viscosity of 1-Ethyl-3-Methylimidazolium Ethyl Sulfate. *J. Chem. Thermodyn.* **47**, 68-75 (2012).

39. Jacquemin, J. &Husson, P. Comments and Additional Work on ‘High-Pressure Volumetric Properties of Imidazolium-Based Ionic Liquids: Effect of the Anion’. *J. Chem. Eng. Data* **57**, 2409-2414 (2012).

40. Carvalho, P. J. *et al*. High Pressure Density and Solubility for the CO_2_ + 1-Ethyl-3-Methylimidazolium Ethylsulfate System. *J. Supercrit. Fluids***88**, 46-55 (2014).

41. Guerrero, H., García-Mardones, M., Cea, P., Lafuente, C. &Bandrés, I. Correlation of the Volumetric Behaviour of Pyridinium-Based Ionic Liquids with Two Different Equations. *Thermochim. Acta* **531**, 21-27 (2012).

42. Safarov, J., Kul, I., El-Awady, W. A., Shahverdiyev, A. & Hassel, E. Thermodynamic Properties of 1-Butyl-3-Methylpyridinium Tetrafluoroborate. *J. Chem. Thermodyn.* **43**, 1315-1322 (2011).

43. Safarov, J. *et al*. Thermophysical Properties of 1-Butyl-4-Methylpyridinium Tetrafluoroborate. *J. Chem. Thermodyn.* **51**, 82-87 (2012).

44. Safarov, J. & Hassel, E. Thermodynamic Properties of 1-Hexyl-3-Methylimidazolium Tetrafluoroborate. *J. Mol. Liq.* **153**, 153-158 (2010).

45. Gardas, R. L. *et al*. Densities and Derived Thermodynamic Properties of Imidazolium-, Pyridinium-, Pyrrolidinium-, and Piperidinium-Based Ionic Liquids. *J. Chem. Eng. Data* **53**, 805-811 (2008).

46. Harris, K. R., Kanakubo, M. & Woolf, L. A. Temperature and Pressure Dependence of the Viscosity of the Ionic Liquids 1-Methyl-3-Octylimidazolium Hexafluorophosphate and 1-Methyl-3-Octylimidazolium Tetrafluoroborate. *J. Chem. Eng. Data* **51**, 1161-1167 (2006).

47. Kanakubo, M., Harris, K. R., Tsuchihashi, N., Ibuki, K. & Ueno, M. Temperature and Pressure Dependence of the Electrical Conductivity of the Ionic Liquids 1-Methyl-3-Octylimidazolium Hexafluorophosphate and 1-Methyl-3-Octylimidazolium Tetrafluoroborate. *Fluid Phase Equilib.* **261**, 414-420 (2007).

48. Harris, K. R., Woolf, L. A. &Kanakubo, M. Temperature and Pressure Dependence of the Viscosity of the Ionic Liquids 1-Butyl-3-methylimidazolium Hexafluorophosphate. *J. Chem. Eng. Data* **50**, 1777-1782 (2005).

49. Tomida, D., Kumagai, A., Kenmochi, S., Qiao, K. & Yokoyama, C. Viscosity of 1-Hexyl-3-Methylimidazolium Hexafluorophosphate and 1-Octyl-3-Methylimidazolium Hexafluorophosphate at High Pressure. *J. Chem. Eng. Data* **52**, 577-579 (2007).

50. Harris, K. R., Kanakubo, M. & Woolf, L. A. Temperature and Pressure Dependence of the Viscosity of the Ionic Liquids 1-Hexyl-3-Methylimidazolium Hexafluorophosphate and 1-Butyl-3-Methylimidazolium Bis(trifluoromethylsulfonyl)imide. *J. Chem. Eng. Data* **52**, 1080-1085 (2007).

51. Dávila, M. J., Aparicio, S., Alcalde, R., García, B. & Leal, J. M. On the Properties of 1-Butyl-3-Methylimidazolium Octylsulfate Ionic Liquid. *Green Chem.* **9**, 221-232 (2007).

52. Safarov, J., El-Awady, W. A., Shahverdiyev, A. & Hassel, E. Thermodynamic Properties of 1-Ethyl-3-Methylimidazolium Bis(trifluoromethylsulfonyl)imide. *J. Chem. Eng. Data* **56**, 106-112(2011).

53. Jacquemin, J. *et al*. Prediction of Ionic Liquid Properties. II. Volumetric Properties as a Function of Temperature and Pressure. *J. Chem. Eng. Data* **53**, 2133-2143 (2008).

54. Esperança, J. M. S. S. *et al*. Density, Speed of Sound, and Derived Thermodynamic Properties of Ionic Liquids over an Extended Pressure Range. 4. [C_3_mim][NTf_2_] and [C_5_mim][NTf_2_]. *J. Chem. Eng. Data* **51**, 2009-2015 (2006).

55. Widowati, E. & Lee, M.-J. P–V–T Properties of Binary Mixtures of the Ionic Liquid 1-Butyl-3-Methylimidazolium Bis(trifluoromethylsulfonyl)imide with Anisole or Acetophenone at Elevated Pressures. *J. Chem. Thermodyn.* **63**, 95-101 (2013).

56. Gomes de Azevedo, R. *et al*. Thermophysical and Thermodynamic Properties of Ionic Liquids over an Extended Pressure Range: [bmim][NTf_2_] and [hmim][NTf_2_]. *J. Chem. Thermodyn.* **37**, 888-899 (2005).

57. Harris, K. R., Woolf, L. A., Kanakubo, M. &Rüther, T. Transport Properties of N-Butyl-N-MethylpyrrolidiniumBis(trifluoromethylsulfonyl)amide. *J. Chem. Eng. Data* **56**, 4672-4685 (2011).

58. Regueira, T., Lugo, L. &Fernández, J. Influence of the Pressure, Temperature, Cation and Anion on the Volumetric Properties of Ionic Liquids: New Experimental Values for Two Salts. *J. Chem. Thermodyn.* **58**, 440-448 (2013).

59. Widowati, E. & Lee, M.-J. PVT Properties for Binary Ionic Liquids of 1-Methyl-1-Propylpiperidinium Bis(trifluoromethylsulfonyl)imide with Anisole or Acetophenone at Pressures up to 50MPa. *J. Chem. Thermodyn.* **49**, 54-61 (2012).

60. Kandil, M. E., Marsh, K. N. & Goodwin, A. R. H. Measurement of the Viscosity, Density, and Electrical Conductivity of 1-Hexyl-3-Methylimidazolium Bis(trifluorosulfonyl)imide at Temperatures between (288 and 433) K and Pressures below 50 MPa. *J. Chem. Eng. Data* **52**, 2382-2387 (2007).

61. Esperança, J. M. S. S., Guedes, H. J. R., Lopes, J. N. C. &Rebelo, L. P. N. Pressure−Density−Temperature (P−ρ−T) Surface of [C_6_mim][NTf_2_]. *J. Chem. Eng. Data* **53**, 867-870 (2008).

62. Iguchi, M. *et al*. Measurement of High-Pressure Densities and Atmospheric Viscosities of Ionic Liquids: 1-Hexyl-3-Methylimidazolium Bis(trifluoromethylsulfonyl)imide and 1-Hexyl-3-Methylimidazolium Chloride. *J. Chem. Eng. Data* **59**, 709-717 (2014).

63. Safarov, J. *et al*. Thermophysical Properties of 1-Hexyl-3-Methylimidazolium Bis(trifluoromethylsulfonyl)imide at High Temperatures and Pressures. *J. Mol. Liq.* **187**, 137-156 (2013).

64. Almantariotis, D., Fandiño, O., Coxam, J.-Y. & Costa Gomes, M.F. Direct Measurement of the Heat of Solution and Solubility of Carbon Dioxide in 1-Hexyl-3-Methylimidazolium Bis[trifluoromethylsulfonyl]amide and 1-Octyl-3-Methylimidazolium Bis[trifluoromethylsulfonyl]amide. *Int. J. Greenh. Gas Control* **10**, 329-340 (2012).

65. Esperança, J. M. S. S., Guedes, H. J. R., Blesic, M. &Rebelo, L. P. N. Densities and Derived Thermodynamic Properties of Ionic Liquids. 3. Phosphonium-Based Ionic Liquids over an Extended PressureRange. *J. Chem. Eng. Data* **51**, 237-242 (2006).

66. Gonçalves, F. A. M. M. *et al*. Pressure-Volume-Temperature Measurements of Phosphonium-Based Ionic Liquids and Analysis with Simple Equations of State. *J. Chem. Thermodyn.* **43**, 914-929 (2011).

67. Tomé, L. I. N. *et al*. Measurements and Correlation of High-Pressure Densities of Phosphonium Based Ionic Liquids. *J. Chem. Eng. Data* **56**, 2205-2217 (2011).

68. Almantariotis, D. *et al*. Absorption of Carbon Dioxide, Nitrous Oxide, Ethane and Nitrogen by 1-Alkyl-3-Methylimidazolium (C_n_mim, n = 2,4,6) Tris(pentafluoroethyl)trifluorophosphate Ionic Liquids (eFAP). *J. Phys. Chem. B* **116**, 7728-7738 (2012).

69. Stevanovic, S. & Costa Gomes, M. F. Solubility of Carbon Dioxide, Nitrous Oxide, Ethane, and Nitrogen in 1-Butyl-1-Methylpyrrolidinium and Trihexyl(tetradecyl)phosphoniumTris(pentafluoroethyl)-trifluorophosphate (eFAP) Ionic Liquids. *J. Chem. Thermodyn.* **59**, 65-71 (2013).

70. Ferreira, C. E., Talavera-Prieto, N. M. C., Fonseca, I. M. A., Portugal, A. T. G. & Ferreira, A. G. M. Measurements of pVT, Viscosity, and Surface Tension of TrihexyltetradecylphosphoniumTris(pentafluoroethyl)trifluorophosphate Ionic Liquid and Modelling with Equations of State. *J. Chem. Thermodyn.* **47**, 183-196 (2012).

71. Klomfar, J., Součková, M. &Pátek, J. Temperature Dependence Measurements of the Density at 0.1 MPa for 1-Alkyl-3-Methylimidazolium-Based Ionic Liquids with the Trifluoromethanesulfonate and Tetrafluoroborate Anion. *J. Chem. Eng. Data* **55**, 4054-4057 (2010).

72. Gaciño, F. M., Regueira, T., Comuñas, M. J. P., Lugo, L. &Fernández, J. Density and Isothermal Compressibility for Two Trialkylimidazolium-Based Ionic Liquids at Temperatures from (278 to 398)K and up to 120MPa. *J. Chem. Thermodyn.* **81**, 124-130 (2015).

73. Talavera-Prieto, N. M. C. *et al*. Thermophysical Characterization of N-Methyl-2-Hydroxyethylammonium Carboxilate Ionic Liquids. *J. Chem. Thermodyn.* **68**, 221-234 (2014).

74. Gaciño, F. M. *et al*. Volumetric Behaviour of Six Ionic Liquids from T=(278 to 398)K and up to 120MPa. *J. Chem. Thermodyn.* **93**, 24-33 (2016).

75. Guerrero, H., Martín, S., Pérez-Gregorio, V., Lafuente, C. &Bandrés, I. Volumetric Characterization of Pyridinium-Based Ionic Liquids. *Fluid Phase Equilib.* **317**, 102-109 (2012).

76. Engelmann, M., Schmidt, H., Safarov, J., Nocke, J. & Hassel, E. Thermal Properties of 1-Butyl-3-Methylimidazolium Dicyanamide at High Pressures and Temperatures. *ActaChim. Slovaca* **5**, 86-94(2012).

77. Hiraga, Y. *et al*. Separation Factors for [amim]Cl–CO_2_ Biphasic Systems from High Pressure Density and Partition Coefficient Measurements. *Sep. Purif. Technol.* **155**, 139-148 (2015).

78. Matkowska, D. &Hofman, T. Volumetric Properties of the {x_1_[C_4_mim][MeSO_4_] + (1 - x_1_)MeOH} System at Temperatures from (283.15 to 333.15) K and Pressures from (0.1 to 35) MPa. *J. Solution Chem.* **42**, 979-990 (2013).

79. Tomida, D., Kenmochi, S., Tsukada, T., Qiao, K. & Yokoyama, C. Thermal Conductivities of [bmim][PF_6_], [hmim][PF_6_], and [omim][PF_6_] from 294 to 335 K at Pressures up to 20 MPa. *Int. J. Thermophys.* **28**, 1147-1160 (2007).

80. Abdulagatov, I. M., Safarov, J., Guliyev, T., Shahverdiyev, A. & Hassel, E. High Temperature and High Pressure Volumetric Properties of (Methanol + [BMIM^+^][OcSO_4_^−^]) Mixtures. *Phys. Chem. Liq.* **47**, 9-34 (2009).

81. Kanakubo, M. & Harris, K. R. Density of 1-Butyl-3-Methylimidazolium Bis(trifluoromethanesulfonyl)amide and 1-Hexyl-3-Methylimidazolium Bis(trifluoromethanesulfonyl)amide over an Extended Pressure Range up to 250 MPa. *J. Chem. Eng. Data* **60**, 1408-1418 (2015).

82. Hiraga, Y., Kato, A., Sato, Y. & Smith, R. L. Densities at Pressures up to 200 MPa and Atmospheric Pressure Viscosities of Ionic Liquids 1-Ethyl-3-methylimidazolium Methylphosphate, 1-Ethyl-3-methylimidazolium Diethylphosphate, 1-Butyl-3-methylimidazolium Acetate, and 1-Butyl-3-methylimidazolium Bis(trifluoromethylsulfonyl)imide. *J. Chem. Eng. Data* **60**, 876-885 (2015).
